# Comparative analysis of the complete mitochondrial genomes of four cordyceps fungi

**DOI:** 10.1002/ece3.8818

**Published:** 2022-04-25

**Authors:** Can Zhong, Jian Jin, Rongrong Zhou, Hao Liu, Jing Xie, Dan Wan, Shengen Xiao, Shuihan Zhang

**Affiliations:** ^1^ 12575 Horticulture and Landscape College Hunan Agricultural University Changsha China; ^2^ 118393 Institute of Chinese Materia Medica Hunan Academy of Chinese Medicine Changsha China; ^3^ Changchun University of Chinese Medicine Changchun China

**Keywords:** cordyceps, introns, mitochondrial genome, phylogenetic analysis

## Abstract

Cordyceps is a large group of entomogenous, medicinally important fungi. In this study, we sequenced, assembled, and annotated the entire mitochondrial genome of *Ophiocordyceps xuefengensis*, in addition to comparing it against other three complete cordyceps mitogenomes that were previously published. Comparative analysis indicated that the four complete mitogenomes are all composed of circular DNA molecules, although their sizes significantly differ due to high variability in intron and intergenic region sizes in the *Ophiocordyceps sinensis* and *O*. *xuefengensis* mitogenomes. All mitogenomes contain 14 conserved genes and two ribosomal RNA genes, but varying numbers of tRNA introns. The Ka/Ks ratios for all 14 PCGs and *rps3* were all less than 1, indicating that these genes have been subject to purifying selection. Phylogenetic analysis was conducted using concatenated amino acid and nucleotide sequences of the 14 PCGs and *rps3* using two different methods (Maximum Likelihood and Bayesian analysis), revealing highly supported relationships between *O*. *xuefengensis* and other *Ophiocordyceps* species, in addition to a close relationship with *O*. *sinensis*. Further, the analyses indicated that *cox1* and *rps3* play important roles in population differentiation. These mitogenomes will allow further study of the population genetics, taxonomy, and evolutionary biology of medicinally important cordyceps species.

## Graphical abstract

O*phiocordyceps* xuefengensis is a newly identified cordyceps resource that we previously discovered on Xuefeng mountain in the Hunan province of China. The novel taxa have been widely used as an ethnopharmacological invigorant by the Yao group for a long time. And further molecular phylogenetic and species identification studies are critically needed.

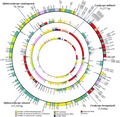

## 1 INTRODUCTION

Mitochondrial genomes are powerful tools that can be used in phylogenetic and evolutionary studies (Cameron et al., 2007; Liu et al., 2018; Saccone et al., 2000; Song et al., 2019), owing to their small size, high copy numbers, conserved orthologous genes, rare instances of recombination, and high evolutionary rates (Curole & Kocher, 1999). The rapid development of new genome sequencing technologies in recent years has led to the production of more than 180 fungal mitogenomes. Fungal mitochondria range in length from approximately 11 kbp (*Hanseniaspora uvarum*) to 272.2 kbp (*Morchella importuna*), depending on the species, but usually contain 14 conserved genes (Pramateftaki et al., 2010). In addition, mitogenome characteristics, including genome size, genome structure, gene content, gene arrangement, tRNA number, intron number, codon usage, and repeat content, can provide useful information to understand the origin, evolution, and systematics of eukaryotes (Poliseno et al., 2017; Qiang et al., 2018; Sankoff et al., 1992).

‘Cordyceps’ comprises four genera including *Cordyceps*, *Ophiocordyceps*, *Metacordyceps*, and *Elaphocordyceps*. The fungi have been used in traditional Chinese medicine, as dietary supplements, or as tonic edibles with broad pharmacological properties including antibacterial, antiviral, and antitumor activities, in addition to the ability to enhance human immunity (Yue et al., 2013; Zhong et al., 2019). Although *Cordyceps* have considerable economic and medicinal significance, wild cordyceps resources are on the verge of exhaustion, leading to many investigators seeking alternatives. Among these, *O*. *xuefengensis* is a newly identified cordyceps resource that we previously discovered on Xuefeng mountain in Hunan province of China (Wen et al., 2013). The novel taxa has been widely used as an ethnopharmacological invigorant by the Yao group for a long time (Jin et al., 2019). Further, we have successfully cultivated this cordyceps fungi and also characterized some of its chemical metabolites (Zhong et al., 2019). *Cordyceps* and *Ophiocordyceps* are the two most widely used groups of cordyceps fungi. In particular, *O*. *sinensis* is popularly used to treat many diseases in China. Consequently, the cultivation, novel metabolite compounds, and pharmacology of cordyceps fungi have been extensively researched, although further molecular phylogenetic and species identification studies are critically needed.

In this study, the complete mitogenome from *O*. *xuefengensis* was sequenced, assembled, and compared to other three complete cordyceps mitogenomes that were previously reported. This study thereby represents the first comparative analysis of the genomic structure, base composition, substitution, and evolutionary rates among four cordyceps species, in addition to the comprehensive molecular phylogenetic analysis of cordyceps in the Hypocreales order. This study aimed to expand our knowledge of the diversity of mitochondrial genomes and phylogenetic relationships of cordyceps.

## 2 MATERIALS AND METHODS

### 2.1 Fungal materials and DNA extraction

*Ophiocordyceps xuefengensis* strain HCMA001 was used in this study for mitogenomic analysis. *In vitro* mycelial fermentation in liquid culture was conducted in Erlenmeyer flasks with shaking at 150 rpm in an incubator at 25°C for 10 days. Mycelia were collected by centrifugation and quickly frozen in liquid nitrogen. Total genomic DNA was then extracted using an improved extraction method (Chen et al., 2011). Genomic DNA was quantified using an TBS‐380 fluorometer (Turner BioSystems Inc., Sunnyvale, CA). High quality DNA samples (OD_260/280_ = 1.8–2.0, >6 μg) were used to construct fragment libraries for genomic sequencing.

### 2.2 Mitochondrial DNA sequencing and genome assembly

About 8 μg of purified DNA was sequenced using a combination of third‐generation sequencing technologies including PacBio RS and Illumina sequencing platforms. The Illumina data were used to evaluate the complexity of the genome and correct the PacBio long reads. The raw paired‐end reads were trimmed, and quality controlled using Trimmomatic with parameters including SLIDINGWINDOW: 4:15 MINLEN: 75 (version 0.36). Clean data obtained from the above quality control procedures were then used for further analyses.

### 2.3 Annotation and comparative analysis of the *Ophiocordyceps xuefengensis* mitogenome

Ab initio prediction was used to generate gene models for the *O*. *xuefengensis* mitogenome. Gene models were identified using MFannot (https://megasun.bch.umontreal.ca/cgi‐bin/dev_mfa/mfannotInterface.pl). Gene models were then compared against the non‐redundant (NR) NCBI database using BLASTp, in addition to comparison against the SwissProt, KEGG, and COG databases to establish functional annotation, also using BLASTp. tRNAs were identified using the tRNAscan‐SE program (v1.23, http://lowelab.ucsc.edu/tRNAscan‐SE) and rRNAs were identified using the RNAmmer program (v1.2, http://www.cbs.dtu.dk/services/RNAmmer/). Circular maps of the four mitochondrial genomes were drawn using OGDRAW (https://chlorobox.mpimp‐golm.mpg.de/OGDraw.htmlonline) (Conant & Wolfe, 2008).

### 2.4 Sequence analysis

Nucleotide compositional skew was calculated according to the formulae: AT‐skew = (A − T)/(A + T) and GC‐skew = (G − C)/(G + C) (Perna & Kocher, 1995). The Sequence Manipulation Suite program package (http://www.bioinformatics.org/sms2/codon_usage.html) was used to analyze codon usage based on genetic code ‘4’ (Stothard, 2000). Genetic distances between each pair of the 14 core PCGs (*atp6*, *atp8*, *atp9*, *cob*, *cox1*, *cox2*, *cox3*, *nad1*, *nad2*, *nad3*, *nad4*, *nad4L*, *nad5*, and *nad6*) were calculated with MEGA X (Joseph, 2016), using the Kimura−2‐parameter (K2P) substitution model. DnaSP v6 (Rozas et al., 2017) was used to calculate the nonsynonymous (Ka) and synonymous (Ks) substitution rates for the 14 core PCGs among the four mitogenomes. Genomic synteny among the four mitogenomes was analyzed using the Mauve v2.4.0 program (Darling et al., 2004).

### 2.5 Repetitive element analysis

Tandem repeats (>10 bp in length) in the four mitogenomes were detected using the Tandem Repeats Finder program (Benson, 1999), with default parameters. In addition, repeat sequences were identified using the REPuter program (Kurtz et al., 2001) to identify forward (direct), reverse, complemented, and palindromic (reverse complemented) repeats across the four mitogenomes, using a minimum repeat size of 30 and a hamming distance set to 3.

### 2.6 Phylogenetic analysis of Hypocrealean species

To determine the phylogenetic relationships of the cordyceps species among the Hypocreales order, 19 mitogenomes were downloaded from the NCBI database and subjected to Maximum Likelihood (ML) and Bayesian phylogenetic analysis using the aforementioned 14 PCGs and *rps3*. *Rhizopogon salebrosus* and *Rhizopogon vinicolor* were used as outgroups for the analyses (Table 1). Single mitochondrial genes were first aligned using MAFFT v7.037 (Katoh et al., 2019) and these alignments were then concatenated using SequenceMatrix v1.7.8 (http://www.softpedia.com/get/Science‐CAD/Sequence‐Matrix.shtml) (Vaidya et al., 2011). The ML tree was then calculated for the combined gene set using RAxML v8.0.0 (Stamatakis, 2014), while Bayesian inference (BI) analysis was performed with MrBayes v3.2.6 (Ronquist et al., 2012). Other analyses and evaluation methods were conducted based on previously described protocols (Qiang et al., 2018).

**TABLE 1:** The GenBank accessions for genomes used in the phylogenetic analysis

Species	Order	Family	Genus	Genome length/bp	GenBank accession number
*Rhizopogon salebrosus*	Agaricomycetes	Agaricomycetidae	*Boletales*	66,704	MH 794152
*Rhizopogon vinicolor*	Agaricomycetes	Agaricomycetidae	*Boletales*	77,109	MH 794153
*Pochonia chlamydosporia*	Hypocreales	Clavicipitaceae	*Pochonia*	25,615	NC_022835.1
*Metarhizium anisopliae*	Hypocreales	Clavicipitaceae	*Metarhizium*	24,673	NC_008068.1
*Cordyceps militaris*	Hypocreales	Cordycipitaceae	*Cordyceps*	33,277	NC_022834.1
*Cordyceps brongniartii*	Hypocreales	Cordycipitaceae	*Cordyceps*	33,926	NC_011194.1
*Beauveria bassiana*	Hypocreales	Cordycipitaceae	*Beauveria*	29,961	NC_010652.2
*Hypomyces aurantius*	Hypocreales	Hypocreaceae	*Hypomyces*	71,638	NC_030206.1
*Fusarium solani*	Hypocreales	Nectriaceae	*Fusarium*	62,978	NC_016680.1
*Fusarium oxysporum*	Hypocreales	Nectriaceae	*Fusarium*	34,477	NC_017930.1
*Fusarium graminearum*	Hypocreales	Nectriaceae	*Fusarium*	95,676	DQ364632.1
*Hirsutella thompsonii*	Hypocreales	Ophiocordycipitaceae	*Hirsutella*	62,509	NC_040165.1
*Hirsutella minnesotensis*	Hypocreales	Ophiocordycipitaceae	*Hirsutella*	52,245	NC_027660.1
*Hirsutella vermicola*	Hypocreales	Ophiocordycipitaceae	*Hirsutella*	53,793	NC_036610.1
*Hirsutella rhossiliensis*	Hypocreales	Ophiocordycipitaceae	*Hirsutella*	62,949	MG979071.1
*Ophiocordyceps sinensis*	Hypocreales	Ophiocordycipitaceae	*Ophiocordyceps*	157,539	NC_034659.1
*Tolypocladium ophioglossoides*	Hypocreales	Ophiocordycipitaceae	*Tolypocladium*	35,159	NC_031384.1
*Tolypocladium inflatum*	Hypocreales	Ophiocordycipitaceae	*Tolypocladium*	25,328	KY924880.1
*Tolypocladium cylindrosporum*	Hypocreales	Ophiocordycipitaceae	*Tolypocladium*	34,698	MN842262.1
*Ophiocordyceps xuefengensis*	Hypocreales	Ophiocordycipitaceae	*Ophiocordyceps*	78,744	This study

### 2.7 Data availability statement

The newly sequenced mitogenome of *O*. *xuefengensis* strain HCMA001 was submitted to GenBank under the accession number SAMN16236787.

## 3 RESULTS

### 3.1 Characterization of the four cordyceps mitogenomes

**FIGURE 1:**
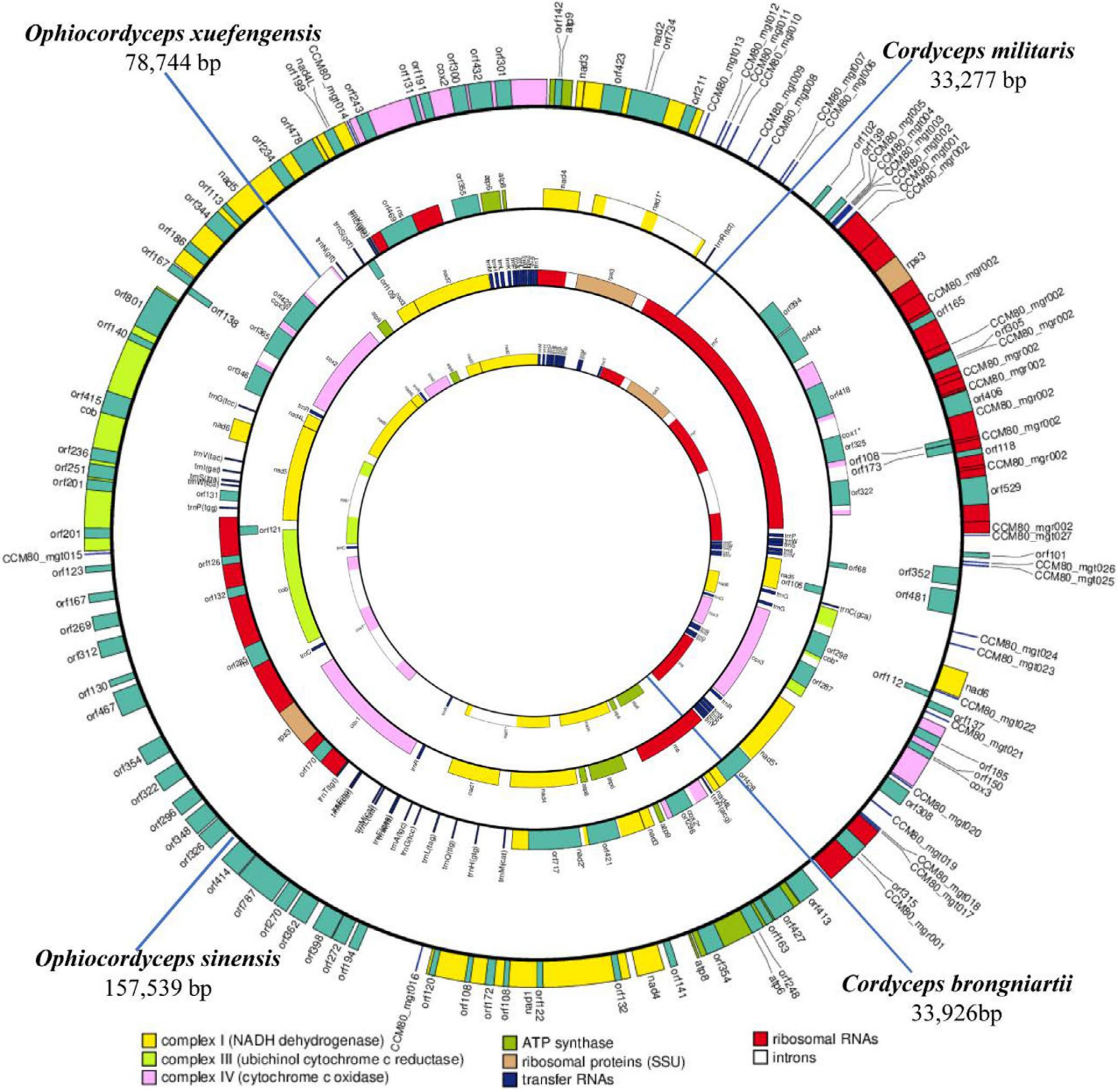
Circular maps of the mitochondrial genomes of four cordyceps species. Genes are represented by different colored blocks. Colored blocks outside each ring indicate that the genes are on the direct strand, while colored blocks within the ring indicate that the genes are located on the reverse strand

Comparative analysis indicated that the complete mitogenomes of the four cordyceps all comprised circular DNA molecules. The mitogenome length of *O*. *xuefengensis* (78,744 bp) was much smaller than that of *O*. *sinensis* (157,559 bp), but longer than those of *Cordyceps militaris* (33,277 bp) and *Cordyceps brongniartii* (33,926 bp) (Figure 1). All four mitogenomes encoded 14 core PCGs involved in energy metabolism, *rps3* involved in translation, and 2 rRNAs. The PCG lengths varied, but the protein coding regions themselves were conserved. The tRNA numbers of the four mitogenomes ranged from 25 (*C*. *brongniartii*) to 27 (*O*. *sinensis*). The tRNAs comprised those for all 20 amino acids and ranged in size from 71 to 86 nucleotides (Table S1). The nucleotide composition of the *O*. *xuefengensis* mitogenome was identical to that of *O*. *sinensis*, but not to *Cordyceps militaris* and *C*. *brongniartii*. The *O*. *sinensis* mitogenome had the highest GC content (30.20%), followed by those of *O*. *xuefengensis* (29.94%), *C*. *brongniartii* (27.34%), and *C*. *militaris* (26.79%). The high frequency of A and T usage in codons contributes to the high AT content of the four mitochondrial genomes, with AT content reaching 70.06% in *O*. *xuefengensis*, 69.80% in *O*. *sinensis*, 73.21% in *C*. *militaris*, and 72.66% in *C*. *brongniartii*. Both GC skew and AT skew were positive for all four mitogenomes (Table 2).

**TABLE 2:** Characteristics of cordyceps mitogenomes

Characteristic	*Ophiocordyceps xuefengensis*	*Ophiocordyceps sinensis*	*Cordyceps militaris*	*Cordyceps brongniartii*
Size (bp)	78,744	157,559	33,277	33,926
A (%)	36.08	36.70	36.98	36.51
T (%)	33.98	33.10	36.23	36.15
G (%)	16.33	15.70	15.20	15.13
C (%)	13.61	13.70	11.59	12.21
Rate of GC (%)	29.94	30.20	26.79	27.34
Rate of AT (%)	70.06	69.80	73.21	72.66
AT‐skew	0.03	0.05	0.01	0.005
GC‐skew	0.09	0.09	0.14	0.11
PCGs (*n*)	14	14	14	14
Introns (*n*)	18	54	8	6
Intronic ORFs (*n*)	16	45	8	5
GIY‐YIG (*n*)	4	10	4	4
LAGLIDADG (*n*)	9	27	3	1
rRNAs (*n*)	1	1	1	1
nRNAl (*n*)	1	1	1	1
tRNAs (*n*)	26	27	26	25
PCG regions (bp)	34,994	104,257	17,735	18,329
Intronic regions (bp)^a^	21,146	107,288	9,812	8,452
Exon regions (bp)^b^	13,848	13,287	12,978	13,098
tRNA genes (bp)	1,934	2,010	1,924	1,867
rRNA genes (bp)^c^	7,225	7,144	4,666	4,801
Intergenic regions (bp)	34,591	27,831	3,797	5,708

^a^ Introns in PCGs and rRNA.

^b^ Exons in PCGs.

^c^ *rnl* and *rns* including exons.

**FIGURE 2:**
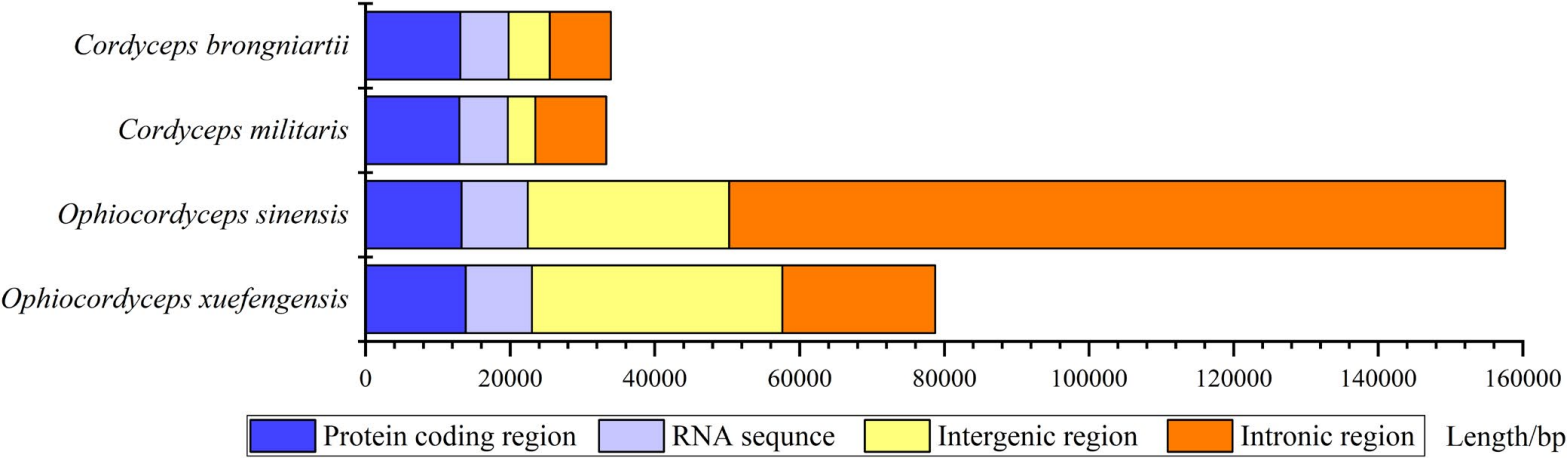
The abundances of protein‐coding, intronic, intergenic, and RNA gene regions (rRNAs and tRNAs) of the entire mitochondrial genomes from four cordyceps species

The *O*. *sinensis* mitogenome exhibited an unusually enlarged size (157,559 bp). The compositions of regions were thus analyzed for the mitogenomes, indicating that the protein coding regions among the four mitogenomes are basically identical. A significant difference was observed when comparing intronic and intergenic regions, wherein the total number of intronic nucleotides of the *O*. *sinensis* mitogenome was much higher than for those of the other cordyceps, and the total number of intergenic nucleotides of the *O*. *xuefengensis* mitogenome is the highest among the four mitogenomes (Figure 2).

The number of introns also significantly differed among the four mitogenomes. 54 introns were present in the mitogenome of *O*. *sinensis*, 18 in that of *O*. *xuefengensis*, 8 in that of *C*. *militaris*, and 6 in that of *C*. *brongniartii* (Table 2). Further, the host genes of introns also differ among the four cordyceps. Introns are distributed in the *cob* (no. of introns: 1), *cox1* (1), *cox2* (1), *cox3* (1), and *rnl* (4) genes of the *C*. *militaris* mitogenome, and in the *cob* (1), *cox1* (2), *nad1* (1), and *rnl* (2) genes of the *C*. *brongniartii* mitogenome. In *O*. *xuefengensis*, the introns are distributed in *cob* (2), *cox1* (3), *cox2* (1), *cox3* (3), *nad1* (2), *nad2* (2), *nad5* (1), *rnl* (3), and *rns* (1) genes, while in the *O*. *sinensis* mitogenome, the introns are distributed in the *cob* (6), *cox1* (14), *cox2* (6), *cox3* (2), *nad1* (3), *nad2* (2), *nad4L* (1), *nad5* (5), *nad6* (1), *atp6* (2), *atp9* (1), *rnl* (8), and *rns* (1) genes (Table 3). The most common intron type among the mitogenomes are group IB introns. Introns also harbor 0–2 homing endonuclease genes, including the LAGLIDADG homing endonuclease and the GIY‐YIG endonuclease.

**TABLE 3:** Information of introns in four cordyceps mitogenome

	*O. xuefengensis*	*O.s sinensis*	*C. militaris*	*C. brongniartii*
Intron type				
IA	1	4	1	1
IB	6	20	3	2
IC1		11	2	2
IC2	4	4		
ID	2	3	1	1
I(derived, A)	2	1	1	
I(derived, B1)		1		
I(derived, B2)		6		
II	1	4		
Unknown	2			
Conserved domain				
LAGLI‐DADG	9	25	3	1
GIY‐YIG	4	7	4	4
Hypothetical protein	2	18		
RNA‐dependent DNA polymerase		1		

### 3.2 Protein‑coding genes and codon usage among the four mitogenomes

**FIGURE 3:**
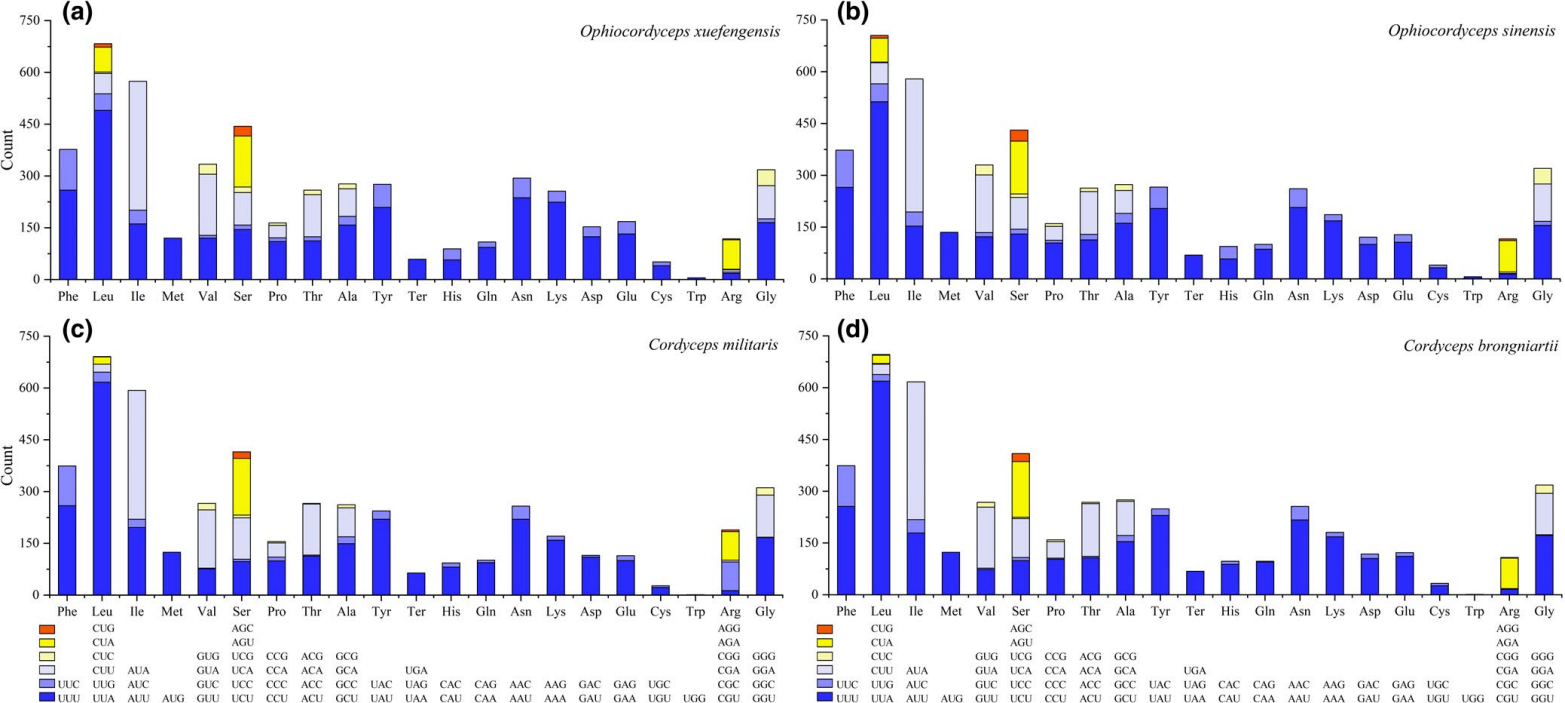
Codon usage in the mitochondrial genomes of the four cordyceps species. Codon numbers are plotted on the y‐axis for a, *O*. *xuefengensis*; b, *O*. *sinensis*; c, *C*. *militaris*; d, *C*. *brongniartii*

**FIGURE 4:**
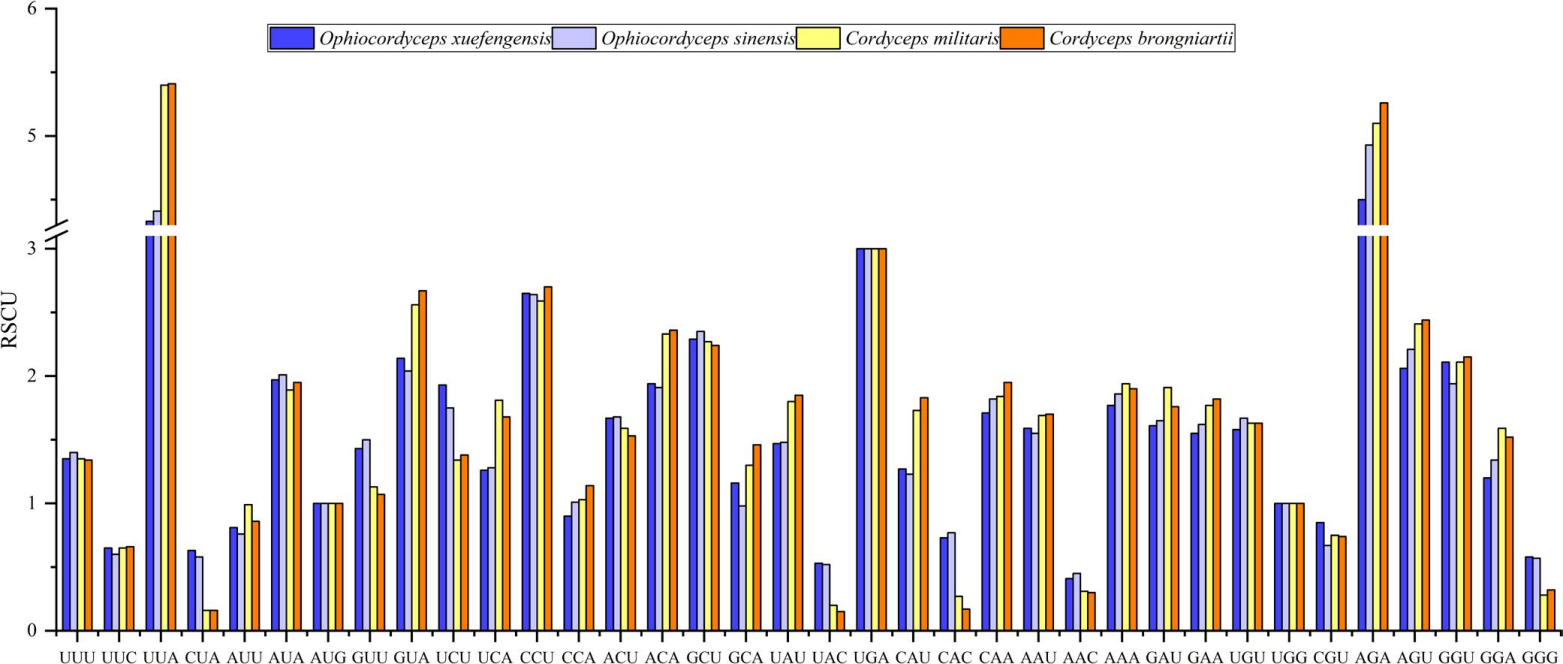
RSCU values for 14 PCGs and *rps3* in the mitochondrial genomes of four cordyceps

Most PCGs and *rps3* are initiated with the ATG codon, whereas *cox1* and *nad6* in the *O*. *xuefengensis* mitogenome are initiated with the TTG codon. Further, *cox3* in the *O*. *sinensis* mitogenome and *atp9* in both *O*. *xuefengensis* and *O*. *sinensis* mitogenomes are initiated with the GTG codon. In addition, most PCGs and *rps3* are terminated with the TAA codon, but *cox1*, *rps3*, and *cox3* in the *O*. *sinensis* mitogenome, and *cox1* and *cob* of the *O*. *xuefengensis* mitogenome are terminated with the TAG codon (Table S2). Comparative analysis indicated that all the PCGs and *rps3* encode 20 amino acids, with similar amino acid compositions among the cordyceps mitogenomes. The most overused amino acid is Leu (ranging in prevalence from 628 to 652), whereas the least used amino acid is Cys (32–48 residues). The usage of Asn, Lys, Asp, and Glu in the *O*. *xuefengensis* mitogenome is much higher than in those of the other three cordyceps (Figure 3). The relative synonymous codon usage (RSCU) of the 14 PCGs and *rps3* in the four cordyceps mitogenomes was also analyzed. A total of 4,616, 4,429, 4,326 and 4,366 codons were observed among the PCGs and *rps3* of the *O*. *xuefengensis*, *O*. *sinensis*, *C*. *militaris*, and *C*. *brongniartii* mitogenomes, respectively. The most used codon among all four mitogenomes is UUA, but the most frequently used codon is AGA in the *O*. *xuefengensis* and *O*. *sinensis* mitogenomes (Figure 4).

### 3.3 Variation, genetic distance, and evolutionary rates of common genes

**FIGURE 5:**
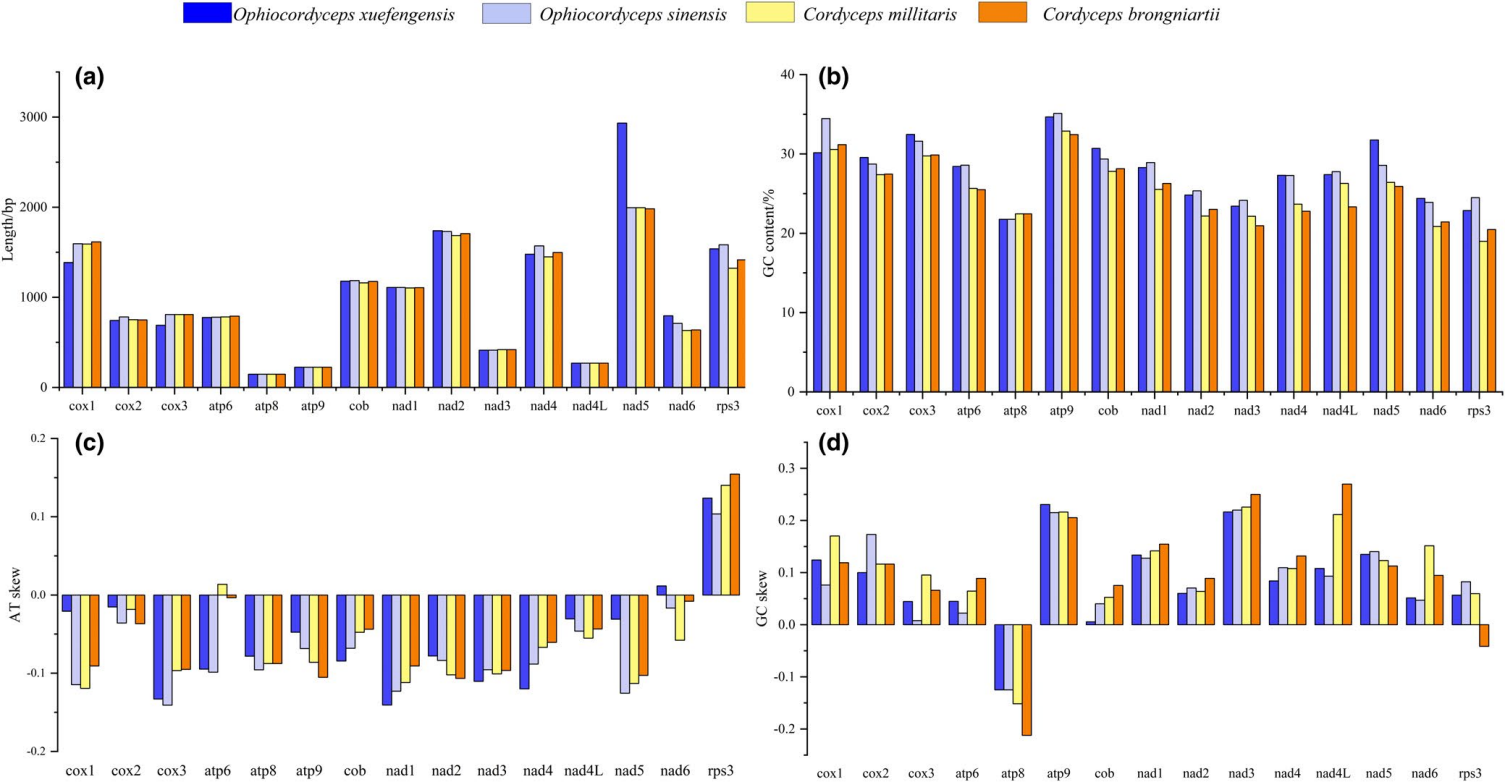
Variation in the length and base composition of each of the 14 PCGs and *rps3* among four cordyceps mitogenomes. a, PCG length variation; b, GC content across PCGs; c, AT skew; d, GC skew

The lengths and/or GC contents of the 14 core PCGs and *rps3* are not all consistent across the four mitogenomes, with the exception of *atp8*, *atp9*, and *nad4L* PCGs. In particular, the length of *nad5* in *O*. *xuefengensis* is about 1,000 bp longer than in the other three cordyceps mitogenomes. Further, the GC content of all 14 PCGs and *rps3* differ, indicating that the core PCGs and *rps3* have been modified within different cordyceps. Across all PCGs in the mitogenomes, GC content is highest in *atp9* within all four cordyceps mitogenomes, and lowest in *atp8* within the *O*. *xuefengnensis* and *O*. *sinensis* mitogenomes, but lowest in *rps3* within the *C*. *militaris* and *C*. *brongniartii* mitogenomes. AT skew is negative in most PCGs except *rps3*, while GC skew is present in most PCGs except *atp8*. In particular, the AT skew of *atp6* in the *C*. *militaris* mitogenome and *nad6* in the *O*. *xuefengnensis* mitogenome are negative, while the GC skew of only *rps3* in the *C*. *brongniartii* mitogenome is negative (Figure 5).

**FIGURE 6:**
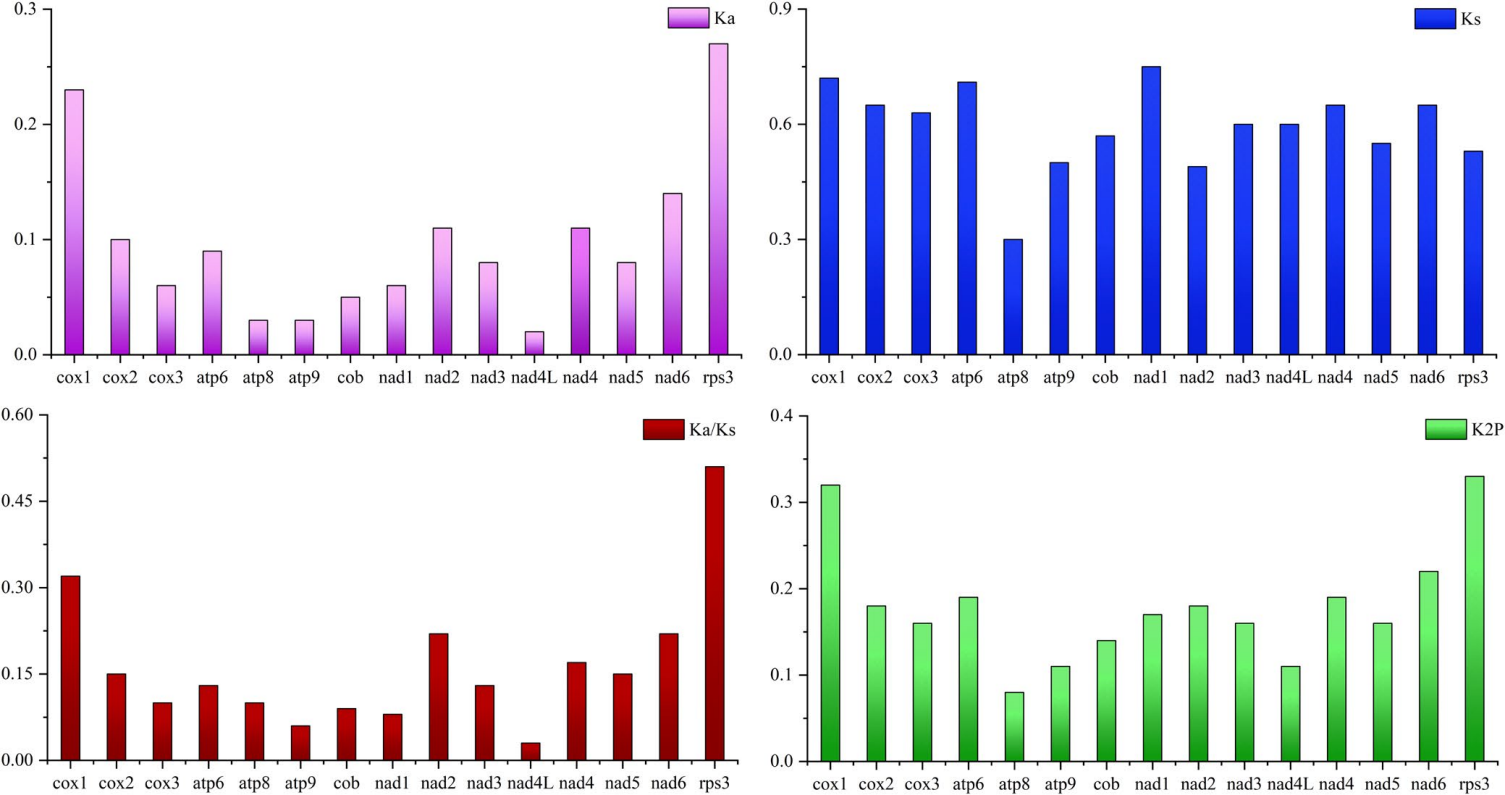
Genetic analysis of 14 PCGs and *rps3* across four cordyceps mitogenomes. K2P, the overall mean Kimura‐2‐Parameter distance; Ka, the mean number of nonsynonymous substitutions per nonsynonymous site; Ks, the mean number of synonymous substitutions per synonymous site

Across all 14 PCGs and *rps3* genes that were evaluated, *rps3* has the greatest K2P genetic distance among the mitogenomes, followed by *cox1*. *atp8* has the least genetic distance among the genes investigated, indicating a high degree of conservation. Ka analysis indicated that *rps3* and *cox1* have relatively high nonsynonymous substitution rates. Further, the Ks of *nad1* is the highest across all four mitogenomes, while that of *atp8* is the lowest. The Ka/Ks values for all 14 PCGs and *rps3* are <1, suggesting that these genes are subject to purifying selection (Figure 6).

### 3.4 Repetitive elements

A total of 18 repeat sequences were identified in the mitogenome of *O*. *xuefengensis*, 250 in that of *O*. *sinensis*, 6 in that of *C*. *militaris* and 23 in that of *C*. *brongniartii* (Table S3). The length of repeat sequences ranges from 38 bp to 372 bp, with pairwise nucleotide similarities ranging from 74.73% to 100%. The largest repeat region was observed in the *O*. *sinensis* mitogenome, within ORF322 and ORF308. The largest repeat region in the *O*. *xuefengensis* mitogenome is 220 bp long and located in the intronic region of *cox2* and ORF301, while the largest repeat region in the *C*. *militaris* mitogenome is 259 bp long and located in the intronic region of *rnl*. Lastly, the largest repeat region in the *C*. *brongniartii* mitogenome is 191 bp long and located between *cox1* and *trnR*.

Seven tandem repeats were detected in both the *O*. *xuefengensis* and *C*. *brongniartii* mitogenomes, 43 in the *O*. *sinensis* mitogenome, and 5 in the *C*. *militaris* mitogenome (Table S4). The longest tandem sequence is found in the *O*. *sinensis* mitogenome, comprises 123 bp, and is located in the in the ORF138 coding region. Of all tandem repeats in the four mitogenomes, most exist in one or two copies. REPuter identified forward (F) and palindromic (P) repeats in the mitogenome of each cordyceps (Table S5), including 69 F and 34 P repeats in the mitogenome of *O*. *xuefengensis* that account for 10.16% of the total mitogenome, along with 16 F and 1 P repeats in the *C*. *militaris* mitogenome that account for 4.95% of its total mitogenome. In addition, 38 F and 19 P repeats were identified in the *C*. *brongniartii* mitogenome that account for 14.54% of its total mitogenome, and a total of 754 F, 538 P, 31 C, and 60 R repeats were identified in the *O*. *sinensis* mitogenome that account for 65.55% of its total mitogenome.

### 3.5 Gene rearrangements and phylogenetic analysis

**FIGURE 7:**
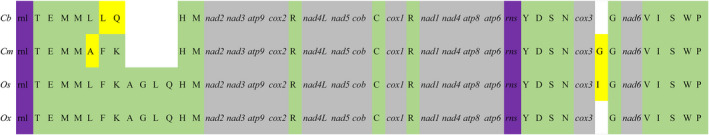
Gene order comparison among four cordyceps mitogenomes. Genes (gray), rRNA (purple), and tRNA (green) conserved across all four species are shown in the same color; and genes that varied across the four mitogenomes are indicated in yellow. *Cb*: *Cordyceps brongniartii*; *Cm*: *Cordyceps militaris*; *Os*: *Ophiocordyceps sinensis*; *Ox*: *Ophiocordyceps xuefengensi*

**FIGURE 8:**
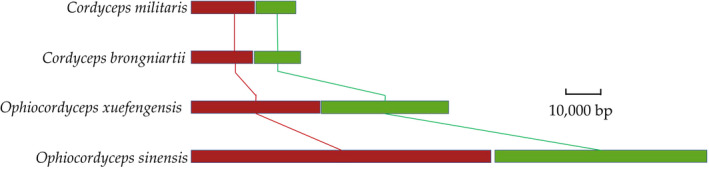
Collinearity analysis of four cordyceps mitogenomes, generated with Mauve 2.4.0. Two homologous regions covered by two different colored blocks were detected across the four mitochondrial genomes overall. The boundaries of colored blocks usually indicate the breakpoints of genome rearrangement, unless sequence has been gained or lost in the breakpoint region. The crossing “X” pattern of lines, which happen to occur in the vicinity of the predicted rearrangements in these organisms

**FIGURE 9:**
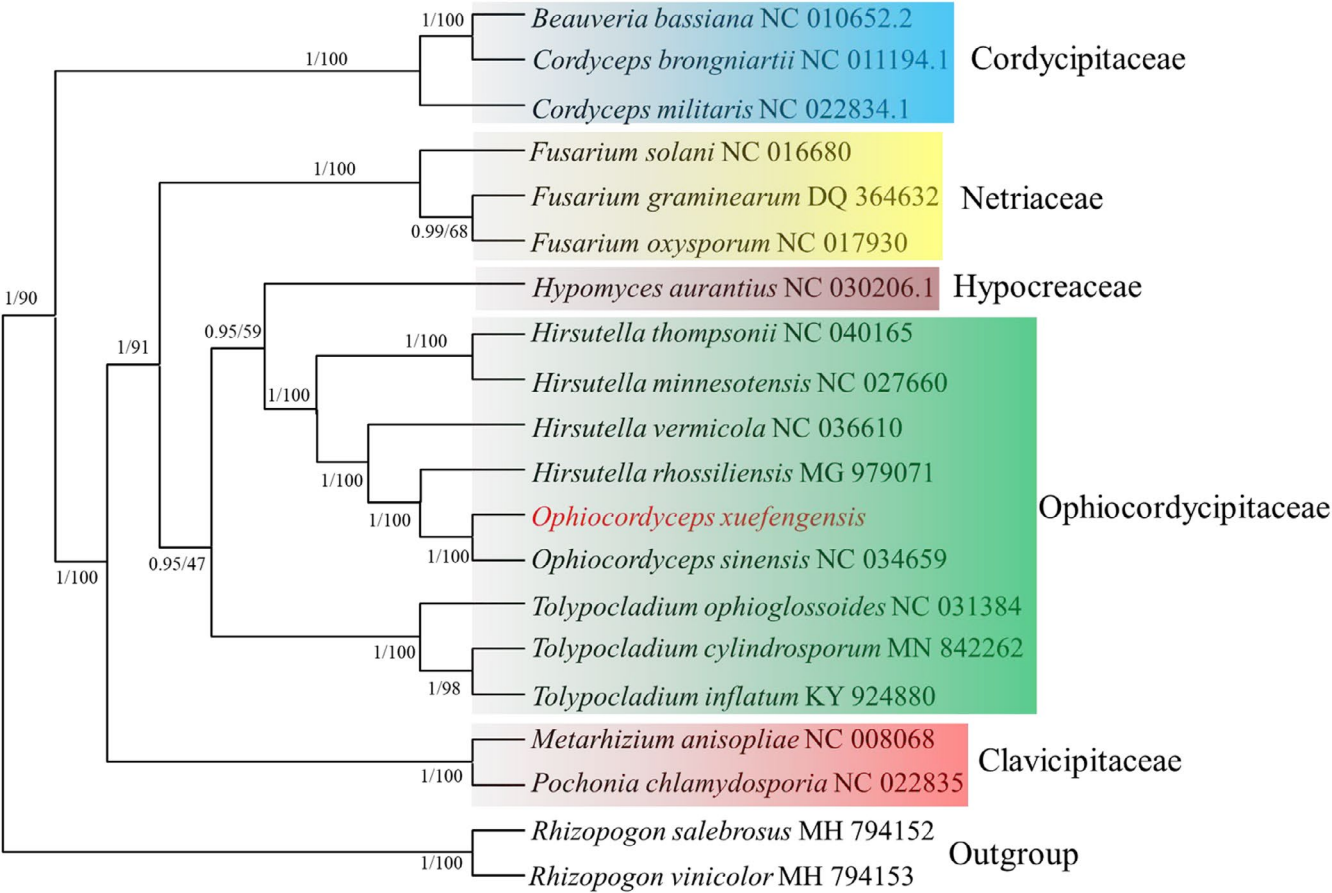
Molecular phylogeny of 20 fungal species based on Bayesian inference (BI) and Maximum likelihood (ML) analysis of 14 PCGs and *rps3* genes. Support values are Bayesian posterior probabilities (before slash) and bootstrap (BS) values (after slash). Species and NCBI accession numbers for genomes used in the phylogenetic analyses are provided in Table 1

The relative positions of the mitochondrial genes (including *rnl*, *rns*, *rps3*, the 14 core PCGs, and the tRNAs) are highly conserved in the four cordyceps mitogenomes. The *O*. *xuefengensis* mitogenome only lacks *trnI*, consistent with that of *O*. *sinensis* (Figure 7). Genome synteny analysis also identified several instances of gene rearrangement in the mitogenomes of the four cordyceps. All four cordyceps mitogenomes could be divided into two homologous regions (Figure 8), with the sizes and relative positions of these homologous regions substantially differing among the four species. Based on the arrangement of homologous regions, there was a high degree of synteny. And mitogenomic rearrangements were not observed among the four species, which indicates that gene recombination did not occur in these four Cordyceps mitochondrial genomes. A complete mitogenome was produced for *O*. *xuefengensis* in this study and used with 19 other complete mitogenome sequences from six families for phylogenetic reconstruction to further investigate the phylogenetic position of *O*. *xuefengensis* within the Hypocreales order. Species and NCBI accession numbers used in these phylogenetic analyses are listed in Table 1. Bayesian inference (BI) based on mitochondrial gene datasets using the GTR +I + G nucleotide substitution model yielded an identical and well‐supported topology in which all major clades are well supported. The 20 fungal species comprised six major clusters, corresponding to the major groups of the orders Hypocreales and Agaricomycetes. The four cordyceps species were divided into two groups, wherein the close relationship between *O*. *xuefengensis* and *O*. *sinensis* was highly supported (Figure 9).

## 4 DISCUSSION

Cordyceps are fungal species used in traditional medicine that are represented by the well‐known fungal resource, *O*. *sinensis*. However, natural populations of *O*. *sinensis* have been overharvested to the extent that it is an endangered species. It is consequently necessary to explore alternative cordyceps resources, because artificial cultivation of *O*. *sinensis* is difficult to achieve (Zhang, Wang, et al., 2015). *Ophiocordyceps xuefengensis* is a new potential cordyceps resource that was found on Xuefeng mountain in Hunan. Investigation of its artificial cultivation and biometabolite biosynthesis yielded some positive results (Zhong et al., 2019). Several previous studies have investigated the phylogenetic relationships of cordyceps, although detailed phylogenetic relationships for the species remain poorly understood, especially since increasing numbers of related species have been reported (Ghikas et al., 2010; Li et al., 2015; Zhang, Zhang, et al., 2015). It is difficult to accurately classify fungal species based on limited morphological characters and overlapping morphological features (Li, Wang, et al., 2019). Consequently, mitogenomes have been widely used in the phylogenetic analysis of eukaryotes due to many advantages, including uniparental inheritance, rapid evolutionary rates, and the presence of several molecular markers (Boore, 1999). Here, we report a new mitogenome sequence for *O*. *xuefengensis* that could provide new insights into the evolutionary histories of cordyceps fungal mitogenomes and the taxonomic relationships among cordyceps.

Mitogenome sizes significantly differ among fungi, ranging from approximately 11.09 kbp (*Hanseniaspora uvarum*) (Pramateftaki et al., 2010) to 272.2 kbp (*Morchella importuna*) (Liu et al., 2020) in length. The variation in size of mitogenomes among different fungal species primarily results from the presence and extent of intergenic regions and introns (Lang et al., 2007). The Ascomycota cordyceps fungus *O*. *sinensis* contained a mitogenome with introns accounting for 68.09% of the whole mitogenome, while introns in the mitogenomes of *O*. *xuefengensis*, *C*. *militaris*, and *C*. *brongniartii* only accounted for 26.85%, 29.49%, and 24.91% of their mitogenomes, respectively. Further, intergenic regions within the *O*. *xuefengensis* mitogenome accounted for 43.93% of the whole mitogenome, but only 11.41%–17.66% of the mitogenomes of the other three cordyceps species. Thus, considerable variation in the size of introns and intergenic regions is the major reason underlying the significant differences in the mitogenome sizes of *O*. *sinensis* and *O*. *xuefengensis*. In contrast, *C*. *militaris* and *C*. *brongniartii* exhibited similarly sized mitogenomes, due to similar numbers and sizes of intergenic regions and introns.

However, the large difference in introns did not affect the taxonomically close relationship between *O*. *sinensis* and *O*. *xuefengensis*. Maximum Likelihood and Bayesian phylogenetic analyses were constructed using 14 PCGs and *rps3* from 20 fungi to reveal the taxonomic relationships of these fungi. The phylogenetic analysis highly supported the close relationship between *O*. *xuefengensis* and *O*. *sinensis*. This apparent domestication of ancestral introns may indicate an adaptation to their host genome that may be due to their decreased mobility (Novikova & Belfort, 2017), or alternatively, to their potential important role in stabilizing the gene that hosts the intron (Genetics, 2003; Korovesi et al., 2018). Other introns may be acquired late in the divergence of taxa, either through HGT events or through active transposition (Mardanov et al., 2014; Wu et al., 2015). The transposition of some introns to other genomic regions with less sequence similarity can occur more frequently under stress‐induced conditions (Coros et al., 2009; Robbins et al., 2011). A recent study demonstrated that the number and Pcl of introns are highly variable between two *Rhizopogon* species (Li, Ren, et al., 2019). Further, the introns in nuclear genes of *S*. *cerevisiae* play crucial roles in the survival of the organism under starvation conditions (Parenteau et al., 2019). Consequently, the abundance of introns in the mitochondrial genome of *O*. *sinensis* relative to other cordyceps species may be a consequence of the particular environments they inhabit.

The accumulation of repeated evolutionary events in fungal mitogenomes could lead to over‐dispersal of repeat sequences and the introduction of new genes through HGT, thereby contributing to dynamic changes in genome structure and gene order (Aguileta et al., 2014). Moreover, the accumulation of repeat sequences could also be highly related to gene recombination and gene loss (Zou, Jakovlić, et al., 2017). A variety of models have been proposed to investigate these mitochondrial gene rearrangements (Xia et al., 2016). Here, the mitogenome of *O*. *sinensis* harbored a larger proportion of repeat sequences than the mitogenomes of *O*. *xuefengensis*, *C*. *militaris* or *C*. *brongniartii*. Interestingly, collinearity analysis revealed that the mitogenomic gene order in these four fungi was not highly variable. In addition, positive selection of core genes was not observed by Ka/Ks analysis. This differs from observations in plants, wherein mitochondrial gene order is highly variable due to high rates of recombination (Aguileta et al., 2014). These observations collectively indicate that selection upon the genomes by environmental factors is relatively weak among the four cordyceps fungi evaluated here, resulting in highly conserved phylogenetic relationships. Thus, we could also infer that differentiation of the cordyceps genomes remains in early stages, consistent with the molecular phylogenetic analyses. One reasonable explanation for these contradictory results is that the larger proportion of repeat sequences in the genome of *O*. *sinensis* exists in intronic regions, but not in intergenic regions, leading to little influence on their evolution.

Mitochondrial genomes are obtained from a common ancestor and have been widely used in population genetics and evolutionary studies (Al‐Reedy et al., 2012; Grosemans et al., 2016). Different barcode genes, including ITS, LSU, RPB2, and EF1α, have been used for phylogenetic analysis (Buyck et al., 2014; Qiang et al., 2018). However, the large number of available molecular markers in mitogenomes and their independent evolutionary histories make them attractive tools for reconstructing phylogenetic relationships (Jiang et al., 2017). Here, we reconstructed a well‐resolved phylogeny based on the combined alignment of 14 PCGs and the *rps3* gene that separated 20 fungal species into major clades. In addition, K2P and Ka/Ks analysis indicated that *cox1* and *rps3* divergence play important roles in population differentiation. Given those reliable genetic molecular markers are critical for further understanding the phylogenetics and classification of species, these two genes could be further evaluated as barcodes for fungal species identification.

As we know, mitogenomes have been widely used in the phylogenetic analysis of plants, fungi, and animals. Studies have shown that there is a certain correlation of genetic relationship, chemical component, and therapeutic effectiveness of medicinal plant. Generally speaking, a closer genetic relationship and higher chemical similarity in plants, and more consistent therapeutic effects expected when similarly categorized (Kim et al., 2018). For example, *Rauvolfia verticillata* (Lour.) Baill. was used instead of *R*. *serpentina* (L.) Benth. exKurz to develop reserpine because they have similar alkaloids and efficacy (Xiao et al., 2021). The genus *Scutellaria* are divided into two branches by molecular systematics, and the chemical components and pharmacological effects of the same group are similar (Shen, 2021), other similar results also occurred in *Malus* (Li et al., 2018) and *Acer* (Bi et al., 2016) genus. Through a series of comparative analyses of mitochondrial genomes, the phylogenetic analysis highly supported the close relationship between *O*. *xuefengensis* and *O*. *sinensis*, suggesting *O*. *xuefengensis* has the potential to be the alternative of *O*. *sinensis*.

## 5 CONCLUSIONS

This study extends the known mitogenomes of cordyceps fungi and identified variation in PCGs, tRNA genes, and rRNA genes among medicinal cordyceps species, including *O*. *xuefengensis*. Comparative mitochondrial genome analysis of four cordyceps fungi was conducted, revealing that their mitochondrial genomes are conserved and that differentiation of their mitogenomes is still in the early stages. Nevertheless, results indicated that the *cox1* and *rps3* genes likely play important roles in population differentiation among the taxa. Mitogenome analyses, like those reported here, will allow further study of the population genetics, taxonomic relationships, and evolutionary biology of medicinally important cordyceps species.

## CONFLICT OF INTEREST

The authors declare no conflicts of interest.

## AUTHOR CONTRIBUTIONS

**Can Zhong:** Project administration (lead); Writing – review & editing (lead). **Jian Jin:** Data curation (supporting); Methodology (supporting); Software (supporting). **Rongrong Zhou:** Methodology (equal). **Hao Liu:** Investigation (equal). **Jing Xie:** Software (supporting); Visualization (supporting). **Dan Wan:** Project administration (lead). **Shengen Xiao:** Conceptualization (equal); Writing – review & editing (supporting). **Shuihan Zhang:** Conceptualization (equal); Funding acquisition (lead); Writing – review & editing (supporting).

### OPEN RESEARCH BADGES

This article has earned a Preregistered Research Designs Badge for having a preregistered research design, available at https://doi.org/10.5281/zenodo.6345492.

## Supporting information

Table S1

Table S2

Table S3

Table S4

Table S5

## References

[ece38818-bib-0001] Aguileta, G. , de Vienne, D. M. , Ross, O. N. , Hood, M. E. , Giraud, T. , Petit, E. , & Gabaldón, T. (2014). High variability of mitochondrial gene order among fungi. Genome Biology and Evolution, 6(2), 451–465. 10.1093/gbe/evu028 24504088 PMC3942027

[ece38818-bib-0002] Al‐Reedy, R. M. , Malireddy, R. , Dillman, C. B. , & Kennell, J. C. (2012). Comparative analysis of Fusarium mitochondrial genomes reveals a highly variable region that encodes an exceptionally large open reading frame. Fungal Genetics, 49(1), 2–14. 10.1016/j.fgb.2011.11.008 22178648

[ece38818-bib-0003] Benson, G. (1999). Tandem repeats finder: A program to analyze DNA sequences. Nucleic Acids Research, 27(2), 573–580. 10.1093/nar/27.2.573 9862982 PMC148217

[ece38818-bib-0004] Bi, W. , Gao, Y. , Shen, J. , He, C. , Liu, H. , Peng, Y. , Zhang, C. , & Xiao, P. (2016). Traditional uses, phytochemistry, and pharmacology of the genus Acer (maple): A review. Journal of Ethnopharmacology, 189, 31–60. 10.1016/j.jep.2016.04.021 27132717

[ece38818-bib-0005] Boore, J. L. J. N. A. R. (1999). Animal mitochondrial genomes. Nucleic Acids Research, 27(8), 1767–1780. 10.1093/nar/27.8.1767 10101183 PMC148383

[ece38818-bib-0006] Buyck, B. , Kauff, F. , Eyssartier, G. , Couloux, A. , & Hofstetter, V. (2014). A multilocus phylogeny for worldwide Cantharellus (Cantharellales, Agaricomycetidae). Fungal Diversity, 64(1), 101–121. 10.1007/s13225-013-0272-3

[ece38818-bib-0007] Cameron, S. L. , Lambkin, C. L. , Barker, S. C. , & Whiting, M. F. (2007). A mitochondrial genome phylogeny of Diptera: Whole genome sequence data accurately resolve relationships over broad timescales with high precision. Systematic Entomology, 32(1), 40–59. 10.1111/j.1365-3113.2006.00355.x

[ece38818-bib-0008] Chen, J. , Guan, R. , Chang, S. , Du, T. , Zhang, H. , & Xing, H. (2011). Substoichiometrically different mitotypes coexist in mitochondrial genomes of *Brassica napus* L. PLoS One, 6(3), e17662. 10.1371/journal.pone.0017662 21423700 PMC3053379

[ece38818-bib-0009] Conant, G. C. , & Wolfe, K. H. (2008). GenomeVx: Simple web‐based creation of editable circular chromosome maps. Bioinformatics, 24(6), 861–862. 10.1093/bioinformatics/btm598 18227121

[ece38818-bib-0010] Coros, C. J. , Piazza, C. L. , Chalamcharla, V. R. , Smith, D. , & Belfort, M. (2009). Global regulators orchestrate group II intron retromobility. Molecular Cell, 34(2), 250–256. 10.1016/j.molcel.2009.03.014 19394301 PMC2690983

[ece38818-bib-0011] Curole, J. P. , & Kocher, T. D. (1999). *Mitogenomics*: digging deeper with complete mitochondrial genomes. Trends in Ecology & Evolution, 14(10), 394–398. 10.1016/S0169-5347(99)01660-2 10481201

[ece38818-bib-0012] Darling, A. C. , Mau, B. , Blattner, F. R. , & Perna, N. T. (2004). Mauve: Multiple alignment of conserved genomic sequence with rearrangements. Genome Research, 14(7), 1394–1403. 10.1101/gr.2289704 15231754 PMC442156

[ece38818-bib-0013] Genetics, B. S. J. C. (2003). Genetic conservation versus variability in mitochondria: The architecture of the mitochondrial genome in the petite‐negative yeast *Schizosaccharomyces pombe* . Current Genetics, 43(5), 311–326. 10.1007/s00294-003-0404-5 12739049

[ece38818-bib-0014] Ghikas, D. V. , Kouvelis, V. N. , & Typas, M. A. J. B. M. (2010). Phylogenetic and biogeographic implications inferred by mitochondrial intergenic region analyses and ITS1‐5.8S‐ITS2 of the entomopathogenic fungi *Beauveria bassiana* and *B. brongniartii* . BMC Microbiology, 10(1), 174–188. 10.1186/1471-2180-10-174 20553589 PMC2896372

[ece38818-bib-0015] Grosemans, T. , Morris, K. , Thomas, W. K. , Rigaux, A. , Moens, T. , & Derycke, S. (2016). Mitogenomics reveals high synteny and long evolutionary histories of sympatric cryptic nematode species. Ecology Evolution, 6(6), 1854–1870. 10.1002/ece3.1975 26933490 PMC4760989

[ece38818-bib-0016] Jiang, L. , Zhao, L. , Cheng, D. , Zhu, L. , Zhang, M. , Ruan, Q. , & Chen, W. (2017). The complete mitochondrial genome sequence of the Sichuan Digging Frog, Kaloula rugifera (Anura: Microhylidae) and its phylogenetic implications. Gene, 626, 367–375. 10.1016/j.gene.2017.05.039 28536079

[ece38818-bib-0017] Jie, S. (2021). The pharmacophylogenetic study of the genus Scutellaria L. in China. Chinese Academy of Medical Sciences and Peking Union Medical College.

[ece38818-bib-0018] Jin, J. , Qin, Y. , Zhong, C. , Zhou, R. , Xie, J. , Liu, H. , Xiao, J. , Cai, P. , Zhang, S. , & Qin, Y. (2019). Differential gene expression and associated metabolite accumulation in fungus *Ophiocordyceps xuefengensis* cultivated under breathable and airtight conditions. Mycoscience, 60(5), 281–286. 10.1016/j.myc.2019.02.009

[ece38818-bib-0019] Joseph, C. (2016). MEGA evolutionary software Re‐engineered to handle today's big data demands. Molecular Biology & Evolution, 33(7), 1887–1888. 10.1093/molbev/msw074 27189553

[ece38818-bib-0020] Katoh, K. , Rozewicki, J. , & Yamada, K. (2019). MAFFT online service: Multiple sequence alignment, interactive sequence choice and visualization. Briefings in Bioinformatics, 20(4), 1160–1166. 10.1093/bib/bbx108 28968734 PMC6781576

[ece38818-bib-0021] Kim, J. H. , Doh, E. J. , & Lee, G. (2018). Chemical differentiation of genetically identified *Atractylodes japonica*, *A. macrocephala*, and *A. chinensis* rhizomes using high‐performance liquid chromatography with chemometric analysis. Evidence‐Based Complementary and Alternative Medicine: Ecam, 2018, 4860371. 30174708 10.1155/2018/4860371 PMC6098908

[ece38818-bib-0022] Korovesi, A. G. , Ntertilis, M. , & Kouvelis, V. N. (2018). Mt‐rps3 is an ancient gene which provides insight into the evolution of fungal mitochondrial genomes. Molecular Phylogenetics Evolution, 127, 74–86. 10.1016/j.ympev.2018.04.037 29763662

[ece38818-bib-0023] Kurtz, S. , Choudhuri, J. V. , Ohlebusch, E. , Schleiermacher, C. , Stoye, J. , & Giegerich, R. (2001). REPuter: The manifold applications of repeat analysis on a genomic scale. Nucleic Acids Research, 22, 4633–4642. 10.1093/nar/29.22.4633 PMC92531 11713313

[ece38818-bib-0024] Lang, B. F. , Laforest, M.‐J. , & Burger, G. (2007). Mitochondrial introns: A critical view. Trends in Genetics, 23(3), 119–125. 10.1016/j.tig.2007.01.006 17280737

[ece38818-bib-0025] Li, Q. , Ren, Y. , Shi, X. , Peng, L. , Zhao, J. , Song, Y. , & Zhao, G. (2019). Comparative mitochondrial genome analysis of two ectomycorrhizal fungi (Rhizopogon) reveals dynamic changes of intron and phylogenetic relationships of the subphylum agaricomycotina. International Journal of Molecular Sciences, 20(20), 5167. 10.3390/ijms20205167 PMC6829451 31635252

[ece38818-bib-0026] Li, Q. , Wang, Q. , Jin, X. , Chen, Z. , Xiong, C. , Li, P. , Zhao, J. , & Huang, W. (2019). Characterization and comparison of the mitochondrial genomes from two Lyophyllum fungal species and insights into phylogeny of Agaricomycetes. International Journal of Biological Macromolecules, 121, 364–372. 10.1016/j.ijbiomac.2018.10.037 30315880

[ece38818-bib-0027] Li, Y. , Hu, X.‐D. , Yang, R.‐H. , Hsiang, T. , Wang, K. , Liang, D.‐Q. , Liang, F. , Cao, D.‐M. , Zhou, F. , Wen, G. , & Yao, Y.‐J. (2015). Complete mitochondrial genome of the medicinal fungus *Ophiocordyceps sinensis* . Scientific Reports, 5, 13892–13900. 10.1038/srep13892 26370521 PMC4570212

[ece38818-bib-0028] Li, P. , Shen, J. , Bi, W. , He, C.‐N. , & Xiao, P.‐G. (2018). Metabolomics analysis and quantitative determination of five components in Malus leaves. Chinese Traditional and Herbal Drugs, 49(22), 5378–5387.

[ece38818-bib-0029] Liu, W. , Cai, Y.‐L. , Zhang, Q.‐Q. , Chen, L.‐F. , Shu, F. , Ma, X.‐L. , & Bian, Y.‐B. (2020). The mitochondrial genome of *Morchella importuna* (272.2kb) is the largest among fungi and contains numerous introns, mitochondrial non‐conserved open reading frames and repetitive sequences. International Journal of Biological Macromolecules, 143, 373–381. 31830457 10.1016/j.ijbiomac.2019.12.056

[ece38818-bib-0030] Liu, Y. , Song, F. , Jiang, P. , Wilson, J.‐J. , Cai, W. , & Li, H. (2018). Compositional heterogeneity in true bug mitochondrial phylogenomics. Molecular Phylogenetics Evolution, 118, 135–144. 10.1016/j.ympev.2017.09.025 28986237

[ece38818-bib-0031] Mardanov, A. V. , Beletsky, A. V. , Kadnikov, V. V. , Ignatov, A. N. , & Ravin, N. V. (2014). The 203 kbp mitochondrial genome of the phytopathogenic fungus sclerotinia borealis reveals multiple invasions of introns and genomic duplications. PLoS One, 9(9), e107536. 10.1371/journal.pone.0107536 25216190 PMC4162613

[ece38818-bib-0032] Novikova, O. , & Belfort, M. (2017). Mobile Group II introns as ancestral eukaryotic elements. Trends in Genetics, 33(11), 773–783. 10.1016/j.tig.2017.07.009 28818345 PMC5659887

[ece38818-bib-0033] Parenteau, J. , Maignon, L. , Berthoumieux, M. , Catala, M. , Gagnon, V. , & Abou Elela, S. (2019). Introns are mediators of cell response to starvation. Nature, 565(7741), 612–617. 10.1038/s41586-018-0859-7 30651641

[ece38818-bib-0034] Perna, N. T. , & Kocher, T. D. (1995). Patterns of nucleotide composition at fourfold degenerate sites of animal mitochondrial genomes. Journal of Molecular Evolution, 41(3), 353–358. 10.1007/BF01215182 7563121

[ece38818-bib-0035] Poliseno, A. , Feregrino, C. , Sartoretto, S. , Aurelle, D. , Wörheide, G. , McFadden, C. S. , & Vargas, S. (2017). Comparative mitogenomics, phylogeny and evolutionary history of Leptogorgia (Gorgoniidae). Molecular Phylogenetics Evolution, 115, 181–189. 10.1016/j.ympev.2017.08.001 28782594

[ece38818-bib-0036] Pramateftaki, P. V. , Kouvelis, V. N. , Panagiotis, L. , & Typas, M. A. (2010). The mitochondrial genome of the wine yeast Hanseniaspora uvarum: A unique genome organization among yeast/fungal counterparts. FEMS Yeast Research, 6(1), 77–90. 10.1111/j.1567-1364.2005.00018.x 16423073

[ece38818-bib-0037] Qiang, L. , Liao, M. , Yang, M. , Xiong, C. , Jin, X. , Chen, Z. , & Huang, W. (2018). Characterization of the mitochondrial genomes of three species in the ectomycorrhizal genus Cantharellus and phylogeny of Agaricomycetes. International Journal of Biological Macromolecules, 118, 756–769. 10.1016/j.ijbiomac.2018.06.129 29959010

[ece38818-bib-0038] Robbins, J. B. , Smith, D. , & Belfort, M. (2011). Redox‐responsive zinc finger fidelity switch in homing endonuclease and intron promiscuity in oxidative stress. Current Biology, 21, 243–248. 10.1016/j.cub.2011.01.008 21256016 PMC3621118

[ece38818-bib-0039] Ronquist, F. , Teslenko, M. , van der Mark, P. , Ayres, D. L. , Darling, A. , Höhna, S. , Larget, B. , Liu, L. , Suchard, M. A. , & Huelsenbeck, J. P. (2012). MrBayes 3.2: Efficient bayesian phylogenetic inference and model choice across a large model space. Systematic Biology, 61(3), 539–542. 10.1093/sysbio/sys029 22357727 PMC3329765

[ece38818-bib-0040] Rozas, J. , Ferrer‐Mata, A. , Sánchez‐DelBarrio, J. C. , Guirao‐Rico, S. , Librado, P. , Ramos‐Onsins, S. E. , & Sánchez‐Gracia, A. (2017). DnaSP 6: DNA sequence polymorphism analysis of large datasets. Molecular Biology Evolution, 34, 3299–3302. 10.1093/molbev/msx248 29029172

[ece38818-bib-0041] Saccone, C. , Gissi, C. , Lanave, C. , Larizza, A. , & Reyes, A. (2000). Evolution of the mitochondrial genetic system: An overview. Genes, 261(1), 153–159. 10.1016/s0378-1119(00)00484-4 11164046

[ece38818-bib-0042] Sankoff, D. , Leduc, G. , Antoine, N. , Paquin, B. , Lang, B. F. , & Cedergren, R. (1992). Gene order comparisons for phylogenetic inference: Evolution of the mitochondrial genome. Proceedings of the National Academy of Sciences of the United States of America, 89(14), 6575–6579. 10.1073/pnas.89.14.6575 1631158 PMC49544

[ece38818-bib-0043] Song, F. , Li, H. , Liu, G.‐H. , Wang, W. , James, P. , Colwell, D. D. , Tran, A. , Gong, S. , Cai, W. , & Shao, R. (2019). Mitochondrial genome fragmentation unites the parasitic lice of eutherian mammals. Systematic Biology, 68(3), 430–440. 10.1093/sysbio/syy062 30239978 PMC6472445

[ece38818-bib-0044] Stamatakis, A. (2014). RAxML version 8: A tool for phylogenetic analysis and post‐analysis of large phylogenies. Bioinformatics, 30(9), 1312–1313. 10.1093/bioinformatics/btu033 24451623 PMC3998144

[ece38818-bib-0045] Stothard, P. (2000). The sequence manipulation suite: JavaScript programs for analyzing and formatting protein and DNA sequences. BioTechniques, 28(6), 1102–1104. 10.2144/00286ir01 10868275

[ece38818-bib-0046] Vaidya, G. , Lohman, D. J. , & Meier, R. (2011). SequenceMatrix: Concatenation software for the fast assembly of multi‐gene datasets with character set and codon information. Cladistics‐the International Journal of the Willi Hennig Society, 27(2), 171–180. 10.1111/j.1096-0031.2010.00329.x 34875773

[ece38818-bib-0047] Wen, T. C. , Zhu, R.‐C. , Kang, J.‐C. , Huang, M.‐H. , Tan, D.‐B. , Ariyawansha, H. , Hyde, K. D. , & Liu, H. (2013). Ophiocordyceps xuefengensis sp. nov. from larvae of Phassus nodus (Hepialidae) in Hunan Province, southern China. Phytotaxa, 123(1), 41–50. 10.11646/phytotaxa.123.1.2

[ece38818-bib-0048] Wu, B. , Buljic, A. , & Hao, W. (2015). Extensive horizontal transfer and homologous recombination generate highly chimeric mitochondrial genomes in yeast. Molecular Biology Evolution, 32, 2559–2570. 10.1093/molbev/msv127 26018571

[ece38818-bib-0049] Xia, Y. , Zheng, Y. , Murphy, R. W. , & Zeng, X. (2016). Intraspecific rearrangement of mitochondrial genome suggests the prevalence of the tandem duplication‐random loss (TDLR) mechanism in *Quasipaa boulengeri* . BMC Genomics, 17(1), 965. 10.1186/s12864-016-3309-7 27881087 PMC5122201

[ece38818-bib-0050] Xiao, P.‐G. , Li, M.‐H. , Hao, D.‐C. , He, C.‐N. , & Xu, L.‐J. (2021). Theoretical innovation and application practice of pharmacophylogeny. Modern Chinese Medicine, 23(9), 1499–1505.

[ece38818-bib-0051] Yue, K. , Ye, M. , Lin, X. , & Zhou, Z. (2013). The artificial cultivation of medicinal Caterpillar Fungus, *Ophiocordyceps sinensis* (Ascomycetes): A review. International Journal of Medicinal Mushrooms, 15(5), 425–434. 10.1615/IntJMedMushr.v15.i5.10 24266368

[ece38818-bib-0052] Zhang, J. , Wang, P. , Wei, X. , Li, L. , Cheng, H. , Wu, Y. , Zeng, W. , Yu, H. , & Chen, Y. (2015). A metabolomics approach for authentication of *Ophiocordyceps sinensis* by liquid chromatography coupled with quadrupole time‐of‐flight mass spectrometry. Food Research International, 76(OCT.PT.3), 489–497. 10.1016/j.foodres.2015.07.025 28455029

[ece38818-bib-0053] Zhang, Y. , Zhang, S. , Zhang, G. , Liu, X. , Wang, C. , & Xu, J. (2015). Comparison of mitochondrial genomes provides insights into intron dynamics and evolution in the caterpillar fungus *Cordyceps militaris* . Fungal Genetics and Biology, 77, 95–107. 10.1016/j.fgb.2015.04.009 25896956

[ece38818-bib-0054] Zhong, C. , Jin, J. , Liu, H. , Cai, Y. , Qin, Y. , Xie, J. , Wang, W.‐Z. , Qin, Y.‐H. , Huang, H.‐Y. , & Zhang, S.‐H. (2019). Research status quo, problems and prospects of *Ophiocordyceps xuefengensis* . Journal of Microbiology, 39(4), 107–114.

[ece38818-bib-0055] Zou, H. , Jakovlić, I. , Chen, R. , Zhang, D. , Zhang, J. , Li, W.‐X. , & Wang, G.‐T. (2017). The complete mitochondrial genome of parasitic nematode *Camallanus cotti*: extreme discontinuity in the rate of mitogenomic architecture evolution within the *Chromadorea class* . BMC Genomics, 18(1), 840. 10.1186/s12864-017-4237-x 29096600 PMC5669012

