# Supplementary material for: Comparative analysis of the complete mitochondrial genomes of four cordyceps fungi
Source: Ecol Evol. 2022 Apr 25;12(4):e8818. doi: 10.1002/ece3.8818 (PMC9036042; doi:10.1002/ece3.8818)
Supplement: Supplementary file 1 — Table S1 [file ECE3-12-e8818-s003.docx]

Table S1 General tRNA features in the four cordyceps mitogenomes

| **Gene** | **Position** | | **Size /bp** | **Product** | **Anticodon** |
| --- | --- | --- | --- | --- | --- |
| ***O. xuefengensis*** | | | | | |
| trnR | 12029 | 12099 | 71 | tRNA-Arg | TCT |
| trnY | 26568 | 26651 | 84 | tRNA-Tyr | GTA |
| trnD | 26674 | 26746 | 73 | tRNA-Asp | GTC |
| trnS | 27315 | 27394 | 80 | tRNA-Ser | GCT |
| trnN | 28188 | 28259 | 72 | tRNA-Asn | GTT |
| trnG | 34926 | 34996 | 71 | tRNA-Gly | TCC |
| trnV | 37010 | 37082 | 73 | tRNA-Val | TAC |
| trnI | 37468 | 37539 | 72 | tRNA-Ile | GAT |
| trnS | 37823 | 37908 | 86 | tRNA-Ser | TGA |
| trnW | 38009 | 38080 | 72 | tRNA-Trp | TCA |
| trnP | 38899 | 38971 | 73 | tRNA-Pro | TGG |
| trnT | 50518 | 50589 | 72 | tRNA-Thr | TGT |
| trnE | 51158 | 51230 | 73 | tRNA-Glu | TTC |
| trnM | 51234 | 51304 | 71 | tRNA-Met | CAT |
| trnM | 52249 | 52321 | 73 | tRNA-Met | CAT |
| trnL | 52360 | 52442 | 83 | tRNA-Leu | TAA |
| trnF | 53026 | 53098 | 73 | tRNA-Phe | GAA |
| trnK | 53104 | 53176 | 73 | tRNA-Lys | TTT |
| trnA | 53691 | 53762 | 72 | tRNA-Ala | TGC |
| trnG | 54110 | 54180 | 71 | tRNA-Gly | TCC |
| trnL | 54920 | 55004 | 85 | tRNA-Leu | TAG |
| trnQ | 55698 | 55771 | 74 | tRNA-Gln | TTG |
| trnH | 56640 | 56712 | 73 | tRNA-His | GTG |
| trnM | 57778 | 57848 | 71 | tRNA-Met | CAT |
| trnR | 66482 | 66552 | 71 | tRNA-Arg | ACG |
| trnC | 75311 | 75382 | 72 | tRNA-Cys | GCA |
| ***O. sinensis*** | | | | | |
| trnT | 19715 | 19786 | 72 | tRNA-Thr | TGT |
| trnE | 20017 | 20089 | 73 | tRNA-Glu | TTC |
| trnM | 20093 | 20163 | 71 | tRNA-Met | CAT |
| trnM | 20166 | 20238 | 73 | tRNA-Met | CAT |
| trnL | 20239 | 20321 | 83 | tRNA-Leu | TAA |
| trnF | 23951 | 24032 | 73 | tRNA-Phe | GAA |
| trnK | 24188 | 24260 | 73 | tRNA-Lys | TTT |
| trnA | 25669 | 25741 | 73 | tRNA-Ala | TGC |
| trnG | 26401 | 26471 | 71 | tRNA-Gly | TCC |
| trnL | 27782 | 27865 | 84 | tRNA-Leu | TAG |
| trnQ | 28238 | 28311 | 74 | tRNA-Gln | TTG |
| trnH | 28500 | 28575 | 76 | tRNA-His | GTG |
| trnM | 29557 | 29627 | 71 | tRNA-Met | CAT |
| trnR | 50205 | 50275 | 71 | tRNA-Arg | ACG |
| trnC | 79892 | 79963 | 72 | tRNA-Cys | GCA |
| trnR | 111323 | 111393 | 71 | tRNA-Arg | TCT |
| trnY | 139615 | 139698 | 84 | tRNA-Tyr | GTA |
| trnD | 139721 | 139793 | 73 | tRNA-Asp | GTC |
| trnN | 140587 | 140658 | 72 | tRNA-Asn | GTT |
| trnS | 140661 | 140741 | 81 | tRNA-Ser | GCT |
| trnI | 146772 | 146843 | 72 | tRNA-Ile | TAT |
| trnG | 148122 | 148192 | 71 | tRNA-Gly | TCC |
| trnV | 151005 | 151077 | 73 | tRNA-Val | TAC |
| trnI | 151708 | 151779 | 72 | tRNA-Ile | GAT |
| trnS | 155672 | 155757 | 86 | tRNA-Ser | TGA |
| trnW | 155865 | 155936 | 72 | tRNA-Trp | TCA |
| trnP | 157390 | 157462 | 73 | tRNA-Pro | TGG |
| ***C. militaris*** | | | | | |
| trnT | 8220 | 8290 | 71 | tRNA-Thr | TGT |
| trnE | 8294 | 8365 | 72 | tRNA-Glu | TTC |
| trnM | 8367 | 8437 | 71 | tRNA-Met | CAT |
| trnM | 8448 | 8520 | 73 | tRNA-Met | CAT |
| trnL | 8524 | 8605 | 82 | tRNA-Leu | TAA |
| trnA | 8610 | 8681 | 72 | tRNA-Ala | TGC |
| trnF | 8683 | 8755 | 73 | tRNA-Phe | GAA |
| trnK | 8792 | 8864 | 73 | tRNA-Lys | TTT |
| trnL | 8940 | 9023 | 84 | tRNA-Leu | / |
| trnH | 9067 | 9139 | 73 | tRNA-His | GTG |
| trnM | 9187 | 9258 | 72 | tRNA-Met | CAT |
| trnR | 14098 | 14168 | 71 | tRNA-Arg | ACG |
| trnC | 19190 | 19259 | 70 | tRNA-Cys | GCA |
| trnR | 22392 | 22462 | 71 | tRNA-Arg | TCT |
| trnY | 28926 | 29009 | 84 | tRNA-Tyr | GTA |
| trnD | 29013 | 29085 | 73 | tRNA-Asp | GTC |
| trnS | 29097 | 29177 | 81 | tRNA-Ser | GCT |
| trnN | 29186 | 29256 | 71 | tRNA-Asn | GTT |
| trnR | 29440 | 29510 | 71 | tRNA-Arg | CCT |
| trnG | 31654 | 31724 | 71 | tRNA-Gly | ACC |
| trnG | 31908 | 31979 | 72 | tRNA-Gly | TCC |
| trnV | 32727 | 32798 | 72 | tRNA-Val | TAC |
| trnI | 32806 | 32877 | 72 | tRNA-Ile | GAT |
| trnS | 32918 | 33002 | 85 | tRNA-Ser | TGA |
| trnW | 33004 | 33075 | 72 | tRNA-Trp | TCA |
| trnP | 33106 | 33177 | 72 | tRNA-Pro | TGG |
| ***C. brongniartii*** | | | | | |
| *trn*T | 6462 | 6532 | 71 | tRNA-Thr | ACA |
| *trn*E | 7047 | 7119 | 73 | tRNA-Glu | GAA |
| *trn*M | 7121 | 7191 | 71 | tRNA-Met | ATG |
| *trn*M | 7573 | 7645 | 73 | tRNA-Met | ATG |
| *trn*L | 7649 | 7730 | 82 | tRNA-Leu | TTA |
| *trn*A | 7734 | 7805 | 72 | tRNA-Ala | GCA |
| *trn*F | 7807 | 7879 | 73 | tRNA-Phe | TTC |
| *trn*K | 7892 | 7964 | 73 | tRNA-Lys | AAA |
| *trn*C | 17122 | 17191 | 79 | tRNA-Cys | TGC |
| *trn*R | 22800 | 22870 | 71 | tRNA-Arg | AGA |
| *trn*L | 7966 | 8049 | 84 | tRNA-Leu | CTA |
| *trn*Q | 8053 | 8125 | 73 | tRNA-Gln | CAA |
| *trn*H | 8168 | 8240 | 73 | tRNA-His | CAC |
| *trn*M | 8297 | 8368 | 72 | tRNA-Met | CAT |
| *trnR* | 11978 | 12048 | 71 | tRNA-Arg | CGT |
| *trn*Y | 30968 | 31053 | 86 | tRNA-Tyr | TAC |
| *trn*D | 31057 | 31129 | 73 | tRNA-Asp | GAC |
| *trn*S | 31200 | 31280 | 81 | tRNA-Ser | AGC |
| *trn*N | 31286 | 31356 | 71 | tRNA-Asn | AAC |
| *trn*G | 32274 | 32344 | 71 | tRNA-Gly | GGA |
| *trn*V | 33489 | 33561 | 73 | tRNA-Val | GTA |
| *trn*I | 33566 | 33637 | 72 | tRNA-Ile | ATC |
| *trn*S | 33675 | 33759 | 85 | tRNA-Ser | TCA |
| *trn*W | 33761 | 33832 | 72 | tRNA-Trp | TGA |
| *trn*P | 33844 | 33915 | 72 | tRNA-Pro | CCA |
