# Supplementary material for: Comparative analysis of the complete mitochondrial genomes of four cordyceps fungi
Source: Ecol Evol. 2022 Apr 25;12(4):e8818. doi: 10.1002/ece3.8818 (PMC9036042; doi:10.1002/ece3.8818)
Supplement: Supplementary file 2 — Table S2 [file ECE3-12-e8818-s004.docx]

Table S2 Complete mitochondrial genome characteristics for the four cordyceps analyzed here.

| Gene name | Items | *O. xuefengensis* | *O. sinensis* | *C. militaris* | *C. brongniartii* |
| --- | --- | --- | --- | --- | --- |
| *cox1* | Length/bp | 6067 | 30792 | 2644 | 4095 |
|  | Protein/aa | 461 | 531 | 530 | 538 |
|  | Introns/n | 3 | 14 | 1 | 2 |
|  | GC content | 30.16 | 34.46 | 30.57 | 31.17 |
|  | AT skew | -0.02 | -0.11 | -0.12 | -0.09 |
|  | GC skew | 0.12 | 0.08 | 0.17 | 0.12 |
|  | Start condon | TTG | ATG | ATG | ATG |
|  | Stop condon | TAG | TAG | TAA | TAA |
| *cox2* | Length/bp | 1837 | 11320 | 1937 | 750 |
|  | Protein/aa | 247 | 260 | 247 | 249 |
|  | Introns/n | 1 | 6 | 1 | 0 |
|  | GC content | 29.57 | 28.74 | 27.39 | 27.47 |
|  | AT skew | -0.02 | -0.04 | -0.02 | -0.04 |
|  | GC skew | 0.1 | 0.17 | 0.12 | 0.12 |
|  | Start condon | ATG | ATG | ATG | ATG |
|  | Stop condon | TAA | TAA | TAA | TAA |
| *cox3* | Length/bp | 4922 | 3723 | 2023 | 810 |
|  | Protein/aa | 229 | 269 | 269 | 269 |
|  | Introns/n | 3 | 2 | 1 | 0 |
|  | GC content | 32.46 | 31.6 | 29.75 | 29.88 |
|  | AT skew | -0.13 | -0.14 | -0.1 | -0.1 |
|  | GC skew | 0.04 | 0.01 | 0.1 | 0.07 |
|  | Start condon | ATG | GTG | ATG | ATG |
|  | Stop condon | TAA | TAG | TAA | TAA |
| *atp6* | Length/bp | 777 | 6248 | 783 | 792 |
|  | Protein/aa | 258 | 259 | 260 | 263 |
|  | Introns/n | 0 | 2 | 0 | 0 |
|  | GC content | 28.43 | 28.59 | 25.67 | 25.51 |
|  | AT skew | -0.09 | -0.1 | 0.01 | 0 |
|  | GC skew | 0.05 | 0.02 | 0.06 | 0.09 |
|  | Start condon | ATG | ATG | ATG | ATG |
|  | Stop condon | TAA | TAA | TAA | TAA |
| *atp8* | Length/bp | 147 | 147 | 147 | 147 |
|  | Protein/aa | 48 | 48 | 48 | 48 |
|  | Introns/n | 0 | 0 | 0 | 0 |
|  | GC content | 21.77 | 21.77 | 22.45 | 22.45 |
|  | AT skew | -0.08 | -0.1 | -0.09 | -0.09 |
|  | GC skew | -0.13 | -0.13 | -0.15 | -0.21 |
|  | Start condon | ATG | ATG | ATG | ATG |
|  | Stop condon | TAA | TAA | TAA | TAA |
| *atp9* | Length/bp | 225 | 1295 | 225 | 225 |
|  | Protein/aa | 74 | 74 | 74 | 74 |
|  | Introns/n | 0 | 1 | 0 | 0 |
|  | GC content | 34.67 | 35.11 | 32.89 | 32.44 |
|  | AT skew | -0.05 | -0.07 | -0.09 | -0.11 |
|  | GC skew | 0.23 | 0.22 | 0.22 | 0.21 |
|  | Start condon | GTG | GTG | ATG | ATG |
|  | Stop condon | TAA | TAA | TAA | TAA |
| *cob* | Length/bp | 3602 | 15429 | 2417 | 2474 |
|  | Protein/aa | 392 | 394 | 386 | 391 |
|  | Introns/n | 2 | 6 | 1 | 1 |
|  | GC content | 30.7 | 29.37 | 27.82 | 28.15 |
|  | AT skew | -0.08 | -0.07 | -0.05 | -0.04 |
|  | GC skew | 0.01 | 0.04 | 0.05 | 0.08 |
|  | Start condon | ATG | ATG | ATG | ATG |
|  | Stop condon | TAG | TAA | TAA | TAA |
| *nad1* | Length/bp | 4763 | 11445 | 1104 | 2520 |
|  | Protein/aa | 369 | 369 | 367 | 368 |
|  | Introns/n | 2 | 4 | 0 | 1 |
|  | GC content | 28.29 | 28.92 | 25.54 | 26.29 |
|  | AT skew | -0.14 | -0.12 | -0.11 | -0.09 |
|  | GC skew | 0.13 | 0.13 | 0.14 | 0.15 |
|  | Start condon | ATG | ATG | ATG | ATG |
|  | Stop condon | TAA | TAA | TAA | TAA |
| *nad2* | Length/bp | 5409 | 6828 | 1686 | 1707 |
|  | Protein/aa | 579 | 576 | 561 | 568 |
|  | Introns/n | 2 | 3 | 0 | 0 |
|  | GC content | 24.83 | 25.36 | 22.18 | 23.02 |
|  | AT skew | -0.08 | -0.08 | -0.1 | -0.11 |
|  | GC skew | 0.06 | 0.07 | 0.06 | 0.09 |
|  | Start condon | ATG | ATG | ATG | ATG |
|  | Stop condon | TAA | TAA | TAA | TAA |
| *nad3* | Length/bp | 414 | 414 | 420 | 420 |
|  | Protein/aa | 137 | 137 | 139 | 139 |
|  | Introns/n | 0 | 0 | 0 | 0 |
|  | GC content | 23.43 | 24.15 | 22.14 | 20.95 |
|  | AT skew | -0.11 | -0.1 | -0.1 | -0.1 |
|  | GC skew | 0.22 | 0.22 | 0.23 | 0.25 |
|  | Start condon | ATG | ATG | ATG | ATG |
|  | Stop condon | TAA | TAA | TAA | TAA |
| *nad4* | Length/bp | 1479 | 1572 | 1449 | 1497 |
|  | Protein/aa | 492 | 523 | 482 | 498 |
|  | Introns/n | 0 | 0 | 0 | 0 |
|  | GC content | 27.32 | 27.29 | 23.67 | 22.78 |
|  | AT skew | -0.12 | -0.09 | -0.07 | -0.06 |
|  | GC skew | 0.08 | 0.11 | 0.11 | 0.13 |
|  | Start condon | ATG | ATG | ATG | ATG |
|  | Stop condon | TAA | TAA | TAA | TAA |
| *nad4L* | Length/bp | 270 | 1777 | 270 | 270 |
|  | Protein/aa | 89 | 89 | 89 | 89 |
|  | Introns/n | 0 | 1 | 0 | 0 |
|  | GC content | 27.41 | 27.78 | 26.3 | 23.33 |
|  | AT skew | -0.03 | -0.05 | -0.06 | -0.04 |
|  | GC skew | 0.11 | 0.09 | 0.21 | 0.27 |
|  | Start condon | ATG | ATG | ATG | ATG |
|  | Stop condon | TAA | TAA | TAA | TAA |
| *nad5* | Length/bp | 4287 | 11781 | 1997 | 1983 |
|  | Protein/aa | 977 | 664 | 664 | 660 |
|  | Introns/n | 1 | 5 | 0 | 0 |
|  | GC content | 31.77 | 28.57 | 26.42 | 25.92 |
|  | AT skew | -0.03 | -0.13 | -0.11 | -0.1 |
|  | GC skew | 0.14 | 0.14 | 0.12 | 0.11 |
|  | Start condon | ATG | ATG | ATG | ATG |
|  | Stop condon | TAA | TAA | TAA | TAA |
| *nad6* | Length/bp | 795 | 1487 | 633 | 639 |
|  | Protein/aa | 264 | 236 | 210 | 212 |
|  | Introns/n | 0 | 1 | 0 | 0 |
|  | GC content | 24.4 | 23.91 | 20.85 | 21.44 |
|  | AT skew | 0.01 | -0.02 | -0.06 | -0.01 |
|  | GC skew | 0.05 | 0.05 | 0.15 | 0.09 |
|  | Start condon | TTG | ATG | ATG | ATG |
|  | Stop condon | TAA | TAA | TAA | TAA |
| *rps3* | Length/bp | 1539 | 1584 | 1323 | 1416 |
|  | Protein/aa | 512 | 528 | 421 | 472 |
|  | Introns/n | 0 | 0 | 0 | 0 |
|  | GC content | 22.87 | 24.49 | 19 | 20.48 |
|  | AT skew | 0.12 | 0.1 | 0.14 | 0.15 |
|  | GC skew | 0.06 | 0.08 | 0.06 | -0.04 |
|  | Start condon | ATG | ATG | ATG | ATG |
|  | Stop condon | TAA | TAG | TAA | TAA |
