# Supplementary material for: Comparative analysis of the complete mitochondrial genomes of four cordyceps fungi
Source: Ecol Evol. 2022 Apr 25;12(4):e8818. doi: 10.1002/ece3.8818 (PMC9036042; doi:10.1002/ece3.8818)
Supplement: Supplementary file 3 — Table S3 [file ECE3-12-e8818-s002.docx]

Table S3 Local BLAST analysis of the four cordyceps mitogenomes against themselves.

| Species | Identitie (%) | Aligned length (bp) | Mismatched bases (bp) | Gaps (bp) | from | to | from | to | evalue |
| --- | --- | --- | --- | --- | --- | --- | --- | --- | --- |
| *O. xuefengensis* | 100 | 138 | 0 | 0 | 37569 | 37706 | 35329 | 35466 | 5.51E-68 |
| *O. xuefengensis* | 100 | 138 | 0 | 0 | 35329 | 35466 | 37569 | 37706 | 5.51E-68 |
| *O. xuefengensis* | 89.655 | 174 | 12 | 5 | 17753 | 17923 | 417 | 587 | 2.60E-56 |
| *O. xuefengensis* | 89.535 | 172 | 16 | 2 | 417 | 587 | 17753 | 17923 | 2.60E-56 |
| *O. xuefengensis* | 84.091 | 220 | 22 | 10 | 41630 | 41839 | 39934 | 40150 | 2.62E-51 |
| *O. xuefengensis* | 84.091 | 220 | 22 | 10 | 39934 | 40150 | 41630 | 41839 | 2.62E-51 |
| *O. xuefengensis* | 95.385 | 65 | 2 | 1 | 52460 | 52523 | 49407 | 49343 | 7.60E-22 |
| *O. xuefengensis* | 95.385 | 65 | 2 | 1 | 49343 | 49407 | 52523 | 52460 | 7.60E-22 |
| *O. xuefengensis* | 100 | 51 | 0 | 0 | 55524 | 55574 | 53516 | 53566 | 1.27E-19 |
| *O. xuefengensis* | 100 | 51 | 0 | 0 | 53516 | 53566 | 55524 | 55574 | 1.27E-19 |
| *O. xuefengensis* | 80.992 | 121 | 14 | 9 | 70934 | 71048 | 70871 | 70988 | 2.13E-17 |
| *O. xuefengensis* | 80.992 | 121 | 14 | 9 | 70871 | 70988 | 70934 | 71048 | 2.13E-17 |
| *O. xuefengensis* | 92.727 | 55 | 4 | 0 | 76440 | 76494 | 49344 | 49398 | 3.56E-15 |
| *O. xuefengensis* | 92.727 | 55 | 4 | 0 | 49344 | 49398 | 76440 | 76494 | 3.56E-15 |
| *O. xuefengensis* | 93.617 | 47 | 2 | 1 | 76441 | 76487 | 28102 | 28057 | 7.70E-12 |
| *O. xuefengensis* | 93.478 | 46 | 3 | 0 | 49346 | 49391 | 28102 | 28057 | 7.70E-12 |
| *O. xuefengensis* | 93.478 | 46 | 3 | 0 | 28057 | 28102 | 49391 | 49346 | 7.70E-12 |
| *O. xuefengensis* | 90.566 | 53 | 4 | 1 | 28057 | 28108 | 76487 | 76435 | 7.70E-12 |
| *C. brongniartii* | 89.005 | 191 | 15 | 2 | 22198 | 22388 | 22153 | 22337 | 1.72E-61 |
| *C.brongniartii* | 89.005 | 191 | 15 | 2 | 22153 | 22337 | 22198 | 22388 | 1.72E-61 |
| *C. brongniartii* | 92.784 | 97 | 4 | 2 | 33124 | 33220 | 23909 | 24002 | 3.86E-33 |
| *C. brongniartii* | 92.784 | 97 | 4 | 2 | 23909 | 24002 | 33124 | 33220 | 3.86E-33 |
| *C. brongniartii* | 88.034 | 117 | 7 | 5 | 28916 | 29032 | 23919 | 24028 | 1.80E-31 |
| *C. brongniartii* | 88.034 | 117 | 7 | 5 | 23919 | 24028 | 28916 | 29032 | 1.80E-31 |
| *C. brongniartii* | 96.154 | 78 | 2 | 1 | 25983 | 26060 | 6753 | 6829 | 8.36E-30 |
| *C. brongniartii* | 96.154 | 78 | 2 | 1 | 6753 | 6829 | 25983 | 26060 | 8.36E-30 |
| *C. brongniartii* | 95 | 80 | 1 | 2 | 28947 | 29026 | 6693 | 6617 | 1.08E-28 |
| *C. brongniartii* | 95 | 80 | 1 | 2 | 6617 | 6693 | 29026 | 28947 | 1.08E-28 |
| *C. brongniartii* | 94.667 | 75 | 1 | 3 | 23950 | 24022 | 6691 | 6618 | 6.51E-26 |
| *C. brongniartii* | 94.667 | 75 | 1 | 3 | 6618 | 6691 | 24022 | 23950 | 6.51E-26 |
| *C. brongniartii* | 89.888 | 89 | 7 | 2 | 33134 | 33220 | 28916 | 29004 | 6.51E-26 |
| *C. brongniartii* | 89.888 | 89 | 7 | 2 | 28916 | 29004 | 33134 | 33220 | 6.51E-26 |
| *C. brongniartii* | 95.082 | 61 | 2 | 1 | 21571 | 21630 | 21523 | 21583 | 2.36E-20 |
| *C. brongniartii* | 95.082 | 61 | 2 | 1 | 21523 | 21583 | 21571 | 21630 | 2.36E-20 |
| *C. brongniartii* | 96.364 | 55 | 0 | 2 | 33166 | 33220 | 6691 | 6639 | 1.10E-18 |
| *C. brongniartii* | 92.308 | 65 | 1 | 2 | 6628 | 6688 | 6760 | 6824 | 1.10E-18 |
| *C. brongniartii* | 96.364 | 55 | 0 | 2 | 6639 | 6691 | 33220 | 33166 | 1.10E-18 |
| *C. brongniartii* | 92.188 | 64 | 1 | 2 | 6628 | 6687 | 25990 | 26053 | 3.95E-18 |
| *C. brongniartii* | 87.5 | 72 | 2 | 1 | 11713 | 11784 | 7266 | 7330 | 8.54E-15 |
| *C. brongniartii* | 86.842 | 76 | 0 | 5 | 6627 | 6697 | 10601 | 10671 | 8.54E-15 |
| *C. brongniartii* | 87.5 | 72 | 2 | 1 | 7266 | 7330 | 11713 | 11784 | 8.54E-15 |
| *C. militaris* | 88.417 | 259 | 26 | 4 | 1977 | 2233 | 630 | 886 | 7.44E-85 |
| *C. militaris* | 88.417 | 259 | 26 | 4 | 630 | 886 | 1977 | 2233 | 7.44E-85 |
| *C. militaris* | 100 | 62 | 0 | 0 | 13019 | 13080 | 12957 | 13018 | 1.74E-26 |
| *C. militaris* | 100 | 62 | 0 | 0 | 12957 | 13018 | 13019 | 13080 | 1.74E-26 |
| *C. militaris* | 92.188 | 64 | 5 | 0 | 29442 | 29505 | 29188 | 29251 | 2.93E-19 |
| *C. militaris* | 92.188 | 64 | 5 | 0 | 29188 | 29251 | 29442 | 29505 | 2.93E-19 |
| *O. sinensis* | 96.382 | 304 | 6 | 4 | 70017 | 70318 | 44756 | 45056 | 1.19E-139 |
| *O. sinensis* | 96.382 | 304 | 6 | 4 | 44756 | 45056 | 70017 | 70318 | 1.19E-139 |
| *O. sinensis* | 96.833 | 221 | 3 | 2 | 156014 | 156233 | 24336 | 24553 | 9.77E-101 |
| *O. sinensis* | 96.833 | 221 | 3 | 2 | 24336 | 24553 | 156014 | 156233 | 9.77E-101 |
| *O. sinensis* | 98.361 | 183 | 1 | 2 | 150557 | 150738 | 24378 | 24559 | 7.71E-87 |
| *O. sinensis* | 98.361 | 183 | 1 | 2 | 24378 | 24559 | 150557 | 150738 | 7.71E-87 |
| *O. sinensis* | 97.778 | 180 | 0 | 2 | 156055 | 156233 | 150556 | 150732 | 6.00E-83 |
| *O. sinensis* | 97.778 | 180 | 0 | 2 | 150556 | 150732 | 156055 | 156233 | 6.00E-83 |
| *O. sinensis* | 91.364 | 220 | 12 | 7 | 63536 | 63750 | 63410 | 63627 | 4.68E-79 |
| *O. sinensis* | 91.364 | 220 | 12 | 7 | 63410 | 63627 | 63536 | 63750 | 4.68E-79 |
| *O. sinensis* | 87.645 | 259 | 8 | 4 | 157015 | 157273 | 121022 | 120788 | 1.31E-74 |
| *O. sinensis* | 88.142 | 253 | 6 | 4 | 120794 | 121022 | 157267 | 157015 | 1.31E-74 |
| *O. sinensis* | 97.436 | 156 | 1 | 2 | 147536 | 147688 | 26075 | 26230 | 1.32E-69 |
| *O. sinensis* | 97.436 | 156 | 1 | 2 | 26075 | 26230 | 147536 | 147688 | 1.32E-69 |
| *O. sinensis* | 98.63 | 146 | 0 | 1 | 141114 | 141259 | 140263 | 140406 | 6.13E-68 |
| *O. sinensis* | 98.63 | 146 | 0 | 1 | 140263 | 140406 | 141114 | 141259 | 6.13E-68 |
| *O. sinensis* | 97.315 | 149 | 1 | 2 | 141111 | 141257 | 126030 | 126177 | 1.03E-65 |
| *O. sinensis* | 97.315 | 149 | 1 | 2 | 126030 | 126177 | 141111 | 141257 | 1.03E-65 |
| *O. sinensis* | 98.551 | 138 | 1 | 1 | 147528 | 147665 | 108185 | 108049 | 1.72E-63 |
| *O. sinensis* | 97.241 | 145 | 1 | 2 | 140263 | 140404 | 126033 | 126177 | 1.72E-63 |
| *O. sinensis* | 97.241 | 145 | 1 | 2 | 126033 | 126177 | 140263 | 140404 | 1.72E-63 |
| *O. sinensis* | 98.551 | 138 | 1 | 1 | 108049 | 108185 | 147665 | 147528 | 1.72E-63 |
| *O. sinensis* | 85.081 | 248 | 23 | 14 | 63473 | 63713 | 63410 | 63650 | 6.18E-63 |
| *O. sinensis* | 85.081 | 248 | 23 | 14 | 63410 | 63650 | 63473 | 63713 | 6.18E-63 |
| *O. sinensis* | 97.727 | 132 | 0 | 2 | 108049 | 108177 | 26206 | 26075 | 6.22E-58 |
| *O. sinensis* | 97.727 | 132 | 0 | 2 | 26075 | 26206 | 108177 | 108049 | 6.22E-58 |
| *O. sinensis* | 82.671 | 277 | 17 | 10 | 70439 | 70715 | 45094 | 45339 | 1.04E-55 |
| *O. sinensis* | 82.671 | 277 | 17 | 10 | 45094 | 45339 | 70439 | 70715 | 1.04E-55 |
| *O. sinensis* | 96.212 | 132 | 2 | 2 | 135278 | 135409 | 57033 | 56905 | 1.35E-54 |
| *O. sinensis* | 96.212 | 132 | 2 | 2 | 56905 | 57033 | 135409 | 135278 | 1.35E-54 |
| *O. sinensis* | 95.902 | 122 | 3 | 2 | 108032 | 108152 | 14241 | 14121 | 1.36E-49 |
| *O. sinensis* | 93.333 | 135 | 6 | 2 | 135281 | 135415 | 17520 | 17389 | 1.36E-49 |
| *O. sinensis* | 93.939 | 132 | 5 | 2 | 156372 | 156500 | 56183 | 56314 | 1.36E-49 |
| *O. sinensis* | 95.238 | 126 | 3 | 2 | 17398 | 17520 | 135406 | 135281 | 1.36E-49 |
| *O. sinensis* | 98.214 | 112 | 2 | 0 | 155467 | 155578 | 152174 | 152063 | 1.36E-49 |
| *O. sinensis* | 98.214 | 112 | 2 | 0 | 152063 | 152174 | 155578 | 155467 | 1.36E-49 |
| *O. sinensis* | 93.939 | 132 | 5 | 2 | 56183 | 56314 | 156372 | 156500 | 1.36E-49 |
| *O. sinensis* | 96.61 | 118 | 2 | 1 | 120867 | 120984 | 109927 | 109812 | 4.88E-49 |
| *O. sinensis* | 96.61 | 118 | 2 | 1 | 109812 | 109927 | 120984 | 120867 | 4.88E-49 |
| *O. sinensis* | 96.581 | 117 | 1 | 2 | 120885 | 121001 | 55665 | 55778 | 6.31E-48 |
| *O. sinensis* | 100 | 100 | 0 | 0 | 26988 | 27087 | 17421 | 17520 | 2.94E-46 |
| *O. sinensis* | 100 | 100 | 0 | 0 | 17421 | 17520 | 26988 | 27087 | 2.94E-46 |
| *O. sinensis* | 92.366 | 131 | 9 | 1 | 150132 | 150262 | 57033 | 56904 | 2.94E-46 |
| *O. sinensis* | 92.366 | 131 | 9 | 1 | 56904 | 57033 | 150262 | 150132 | 2.94E-46 |
| *O. sinensis* | 94.309 | 123 | 1 | 3 | 151447 | 151568 | 126679 | 126562 | 1.06E-45 |
| *O. sinensis* | 94.309 | 123 | 1 | 3 | 126562 | 126679 | 151568 | 151447 | 1.06E-45 |
| *O. sinensis* | 92.308 | 130 | 6 | 3 | 56903 | 57030 | 17393 | 17520 | 3.80E-45 |
| *O. sinensis* | 95.652 | 115 | 2 | 2 | 25253 | 25367 | 18775 | 18886 | 3.80E-45 |
| *O. sinensis* | 95.652 | 115 | 2 | 2 | 18775 | 18886 | 25253 | 25367 | 3.80E-45 |
| *O. sinensis* | 92.308 | 130 | 6 | 3 | 17393 | 17520 | 56903 | 57030 | 3.80E-45 |
| *O. sinensis* | 90.647 | 139 | 10 | 2 | 150127 | 150262 | 135273 | 135411 | 3.80E-45 |
| *O. sinensis* | 90.647 | 139 | 10 | 2 | 135273 | 135411 | 150127 | 150262 | 3.80E-45 |
| *O. sinensis* | 98.058 | 103 | 2 | 0 | 120909 | 121011 | 113372 | 113474 | 1.37E-44 |
| *O. sinensis* | 98.058 | 103 | 2 | 0 | 113372 | 113474 | 120909 | 121011 | 1.37E-44 |
| *O. sinensis* | 96.33 | 109 | 4 | 0 | 156373 | 156481 | 135275 | 135383 | 1.37E-44 |
| *O. sinensis* | 96.33 | 109 | 4 | 0 | 135275 | 135383 | 156373 | 156481 | 1.37E-44 |
| *O. sinensis* | 91.406 | 128 | 8 | 2 | 156376 | 156503 | 27090 | 26966 | 2.29E-42 |
| *O. sinensis* | 92 | 125 | 7 | 2 | 26972 | 27093 | 156497 | 156373 | 2.29E-42 |
| *O. sinensis* | 97.98 | 99 | 1 | 1 | 150035 | 150133 | 55465 | 55562 | 8.22E-42 |
| *O. sinensis* | 95.327 | 107 | 5 | 0 | 156015 | 156121 | 109990 | 110096 | 8.22E-42 |
| *O. sinensis* | 97.98 | 99 | 1 | 1 | 55465 | 55562 | 150035 | 150133 | 8.22E-42 |
| *O. sinensis* | 95.327 | 107 | 5 | 0 | 109990 | 110096 | 156015 | 156121 | 8.22E-42 |
| *O. sinensis* | 91.87 | 123 | 6 | 4 | 109978 | 110096 | 24322 | 24444 | 2.96E-41 |
| *O. sinensis* | 94.595 | 111 | 3 | 2 | 135273 | 135383 | 27095 | 26988 | 2.96E-41 |
| *O. sinensis* | 90.84 | 131 | 5 | 2 | 135101 | 135231 | 70442 | 70319 | 2.96E-41 |
| *O. sinensis* | 91.87 | 123 | 6 | 4 | 24322 | 24444 | 109978 | 110096 | 2.96E-41 |
| *O. sinensis* | 90.84 | 131 | 5 | 2 | 70319 | 70442 | 135231 | 135101 | 2.96E-41 |
| *O. sinensis* | 93.86 | 114 | 4 | 2 | 26988 | 27098 | 135383 | 135270 | 2.96E-41 |
| *O. sinensis* | 97.917 | 96 | 2 | 0 | 63618 | 63713 | 63432 | 63527 | 1.06E-40 |
| *O. sinensis* | 97.917 | 96 | 2 | 0 | 63432 | 63527 | 63618 | 63713 | 1.06E-40 |
| *O. sinensis* | 95.238 | 105 | 3 | 1 | 156376 | 156480 | 57033 | 56931 | 3.82E-40 |
| *O. sinensis* | 95.238 | 105 | 3 | 1 | 56931 | 57033 | 156480 | 156376 | 3.82E-40 |
| *O. sinensis* | 91.87 | 123 | 1 | 3 | 157037 | 157159 | 55778 | 55665 | 1.38E-39 |
| *O. sinensis* | 97.849 | 93 | 2 | 0 | 113372 | 113464 | 55686 | 55778 | 4.95E-39 |
| *O. sinensis* | 74.731 | 372 | 88 | 6 | 142016 | 142384 | 93531 | 93899 | 4.95E-39 |
| *O. sinensis* | 91.129 | 124 | 3 | 2 | 157054 | 157177 | 109812 | 109927 | 4.95E-39 |
| *O. sinensis* | 74.731 | 372 | 88 | 6 | 93531 | 93899 | 142016 | 142384 | 4.95E-39 |
| *O. sinensis* | 91.129 | 124 | 3 | 2 | 109812 | 109927 | 157054 | 157177 | 4.95E-39 |
| *O. sinensis* | 96.875 | 96 | 2 | 1 | 135105 | 135200 | 108618 | 108712 | 1.78E-38 |
| *O. sinensis* | 95.146 | 103 | 1 | 2 | 157026 | 157128 | 113474 | 113376 | 1.78E-38 |
| *O. sinensis* | 96.875 | 96 | 2 | 1 | 108618 | 108712 | 135105 | 135200 | 1.78E-38 |
| *O. sinensis* | 95.146 | 103 | 1 | 2 | 113376 | 113474 | 157128 | 157026 | 1.78E-38 |
| *O. sinensis* | 90.244 | 123 | 8 | 4 | 150135 | 150255 | 17520 | 17400 | 6.40E-38 |
| *O. sinensis* | 92.727 | 110 | 7 | 1 | 135275 | 135383 | 56184 | 56293 | 6.40E-38 |
| *O. sinensis* | 92.727 | 110 | 7 | 1 | 56184 | 56293 | 135275 | 135383 | 6.40E-38 |
| *O. sinensis* | 81.517 | 211 | 21 | 14 | 17400 | 17595 | 150255 | 150048 | 6.40E-38 |
| *O. sinensis* | 88.148 | 135 | 10 | 4 | 56177 | 56311 | 27100 | 26972 | 2.30E-37 |
| *O. sinensis* | 94.231 | 104 | 3 | 2 | 56931 | 57033 | 26989 | 27090 | 2.30E-37 |
| *O. sinensis* | 88.148 | 135 | 10 | 4 | 26972 | 27100 | 56311 | 56177 | 2.30E-37 |
| *O. sinensis* | 94.231 | 104 | 3 | 2 | 26989 | 27090 | 56931 | 57033 | 2.30E-37 |
| *O. sinensis* | 87.5 | 136 | 9 | 6 | 126885 | 127019 | 109993 | 110121 | 1.07E-35 |
| *O. sinensis* | 87.5 | 136 | 9 | 6 | 109993 | 110121 | 126885 | 127019 | 1.07E-35 |
| *O. sinensis* | 96.591 | 88 | 2 | 1 | 23748 | 23834 | 14348 | 14435 | 4.98E-34 |
| *O. sinensis* | 96.591 | 88 | 2 | 1 | 14348 | 14435 | 23748 | 23834 | 4.98E-34 |
| *O. sinensis* | 89.655 | 116 | 8 | 4 | 150121 | 150234 | 27102 | 26989 | 4.98E-34 |
| *O. sinensis* | 89.655 | 116 | 8 | 4 | 26989 | 27102 | 150234 | 150121 | 4.98E-34 |
| *O. sinensis* | 90.179 | 112 | 7 | 2 | 110176 | 110286 | 110027 | 110135 | 1.79E-33 |
| *O. sinensis* | 90.179 | 112 | 7 | 2 | 110027 | 110135 | 110176 | 110286 | 1.79E-33 |
| *O. sinensis* | 100 | 76 | 0 | 0 | 24727 | 24802 | 19872 | 19947 | 6.45E-33 |
| *O. sinensis* | 100 | 76 | 0 | 0 | 19872 | 19947 | 24727 | 24802 | 6.45E-33 |
| *O. sinensis* | 87.692 | 130 | 4 | 6 | 135287 | 135405 | 94156 | 94284 | 6.45E-33 |
| *O. sinensis* | 87.692 | 130 | 4 | 6 | 94156 | 94284 | 135287 | 135405 | 6.45E-33 |
| *O. sinensis* | 91.262 | 103 | 6 | 2 | 156379 | 156481 | 17520 | 17421 | 8.34E-32 |
| *O. sinensis* | 90.566 | 106 | 7 | 1 | 56931 | 57033 | 56292 | 56187 | 8.34E-32 |
| *O. sinensis* | 90.566 | 106 | 7 | 1 | 56187 | 56292 | 57033 | 56931 | 8.34E-32 |
| *O. sinensis* | 94.444 | 90 | 3 | 2 | 94163 | 94251 | 57019 | 56931 | 8.34E-32 |
| *O. sinensis* | 90.385 | 104 | 10 | 0 | 63185 | 63288 | 63149 | 63252 | 8.34E-32 |
| *O. sinensis* | 90.385 | 104 | 10 | 0 | 63149 | 63252 | 63185 | 63288 | 8.34E-32 |
| *O. sinensis* | 93.617 | 94 | 2 | 4 | 56934 | 57024 | 94248 | 94156 | 8.34E-32 |
| *O. sinensis* | 91.262 | 103 | 6 | 2 | 17421 | 17520 | 156481 | 156379 | 8.34E-32 |
| *O. sinensis* | 97.468 | 79 | 2 | 0 | 157205 | 157283 | 28151 | 28229 | 3.00E-31 |
| *O. sinensis* | 79.167 | 216 | 30 | 8 | 34440 | 34654 | 30311 | 30512 | 3.00E-31 |
| *O. sinensis* | 79.167 | 216 | 30 | 8 | 30311 | 30512 | 34440 | 34654 | 3.00E-31 |
| *O. sinensis* | 97.468 | 79 | 2 | 0 | 28151 | 28229 | 157205 | 157283 | 3.00E-31 |
| *O. sinensis* | 92.391 | 92 | 5 | 2 | 113470 | 113560 | 55829 | 55919 | 1.40E-29 |
| *O. sinensis* | 92.391 | 92 | 5 | 2 | 55829 | 55919 | 113470 | 113560 | 1.40E-29 |
| *O. sinensis* | 97.368 | 76 | 1 | 1 | 143390 | 143465 | 120948 | 121022 | 5.02E-29 |
| *O. sinensis* | 97.368 | 76 | 1 | 1 | 120948 | 121022 | 143390 | 143465 | 5.02E-29 |
| *O. sinensis* | 95.122 | 82 | 1 | 2 | 157012 | 157093 | 143468 | 143390 | 1.80E-28 |
| *O. sinensis* | 88.571 | 105 | 10 | 1 | 156376 | 156480 | 150132 | 150234 | 1.80E-28 |
| *O. sinensis* | 88.571 | 105 | 10 | 1 | 150132 | 150234 | 156376 | 156480 | 1.80E-28 |
| *O. sinensis* | 95.122 | 82 | 1 | 2 | 143390 | 143468 | 157093 | 157012 | 1.80E-28 |
| *O. sinensis* | 87.719 | 114 | 3 | 4 | 141148 | 141250 | 16574 | 16461 | 2.33E-27 |
| *O. sinensis* | 87.719 | 114 | 3 | 4 | 16461 | 16574 | 141250 | 141148 | 2.33E-27 |
| *O. sinensis* | 95.946 | 74 | 3 | 0 | 27373 | 27446 | 24935 | 24862 | 8.40E-27 |
| *O. sinensis* | 98.529 | 68 | 1 | 0 | 24868 | 24935 | 27440 | 27373 | 8.40E-27 |
| *O. sinensis* | 95.946 | 74 | 2 | 1 | 55863 | 55936 | 27601 | 27529 | 3.02E-26 |
| *O. sinensis* | 97.183 | 71 | 1 | 1 | 27532 | 27601 | 55933 | 55863 | 3.02E-26 |
| *O. sinensis* | 85.833 | 120 | 6 | 4 | 94165 | 94284 | 17507 | 17399 | 1.09E-25 |
| *O. sinensis* | 87.5 | 104 | 9 | 3 | 56190 | 56293 | 17520 | 17421 | 1.09E-25 |
| *O. sinensis* | 87.5 | 104 | 9 | 3 | 17421 | 17520 | 56293 | 56190 | 1.09E-25 |
| *O. sinensis* | 85.833 | 120 | 6 | 4 | 17399 | 17507 | 94284 | 94165 | 1.09E-25 |
| *O. sinensis* | 88.35 | 103 | 3 | 3 | 140297 | 140390 | 16574 | 16472 | 3.91E-25 |
| *O. sinensis* | 90.909 | 88 | 5 | 2 | 94165 | 94251 | 27074 | 26989 | 3.91E-25 |
| *O. sinensis* | 89.583 | 96 | 2 | 3 | 108618 | 108712 | 70438 | 70350 | 3.91E-25 |
| *O. sinensis* | 91.765 | 85 | 4 | 2 | 26992 | 27074 | 94248 | 94165 | 3.91E-25 |
| *O. sinensis* | 89.583 | 96 | 2 | 3 | 70350 | 70438 | 108712 | 108618 | 3.91E-25 |
| *O. sinensis* | 88.35 | 103 | 3 | 3 | 16472 | 16574 | 140390 | 140297 | 3.91E-25 |
| *O. sinensis* | 87.736 | 106 | 2 | 4 | 126067 | 126163 | 16574 | 16471 | 1.41E-24 |
| *O. sinensis* | 95.775 | 71 | 2 | 1 | 109878 | 109947 | 27609 | 27679 | 1.41E-24 |
| *O. sinensis* | 94.521 | 73 | 4 | 0 | 63216 | 63288 | 63144 | 63216 | 1.41E-24 |
| *O. sinensis* | 94.521 | 73 | 4 | 0 | 63144 | 63216 | 63216 | 63288 | 1.41E-24 |
| *O. sinensis* | 95.775 | 71 | 2 | 1 | 27609 | 27679 | 109878 | 109947 | 1.41E-24 |
| *O. sinensis* | 87.736 | 106 | 2 | 4 | 16471 | 16574 | 126163 | 126067 | 1.41E-24 |
| *O. sinensis* | 92.405 | 79 | 4 | 2 | 92709 | 92786 | 23817 | 23740 | 5.05E-24 |
| *O. sinensis* | 92.405 | 79 | 4 | 2 | 23740 | 23817 | 92786 | 92709 | 5.05E-24 |
| *O. sinensis* | 83.333 | 138 | 5 | 6 | 128834 | 128969 | 108739 | 108618 | 5.05E-24 |
| *O. sinensis* | 83.333 | 138 | 5 | 6 | 108618 | 108739 | 128969 | 128834 | 5.05E-24 |
| *O. sinensis* | 95.522 | 67 | 3 | 0 | 150168 | 150234 | 56226 | 56292 | 6.54E-23 |
| *O. sinensis* | 89.655 | 87 | 6 | 3 | 123710 | 123795 | 92890 | 92974 | 6.54E-23 |
| *O. sinensis* | 96.923 | 65 | 1 | 1 | 143390 | 143454 | 113411 | 113474 | 6.54E-23 |
| *O. sinensis* | 89.655 | 87 | 6 | 3 | 92890 | 92974 | 123710 | 123795 | 6.54E-23 |
| *O. sinensis* | 96.923 | 65 | 1 | 1 | 113411 | 113474 | 143390 | 143454 | 6.54E-23 |
| *O. sinensis* | 95.522 | 67 | 3 | 0 | 56226 | 56292 | 150168 | 150234 | 6.54E-23 |
| *O. sinensis* | 95.455 | 66 | 3 | 0 | 150557 | 150622 | 110031 | 110096 | 2.35E-22 |
| *O. sinensis* | 92.105 | 76 | 5 | 1 | 157210 | 157284 | 143426 | 143351 | 2.35E-22 |
| *O. sinensis* | 95.455 | 66 | 3 | 0 | 110031 | 110096 | 150557 | 150622 | 2.35E-22 |
| *O. sinensis* | 92.105 | 76 | 5 | 1 | 143351 | 143426 | 157284 | 157210 | 2.35E-22 |
| *O. sinensis* | 92 | 75 | 5 | 1 | 143352 | 143426 | 28229 | 28156 | 8.46E-22 |
| *O. sinensis* | 90.361 | 83 | 2 | 1 | 108618 | 108694 | 28799 | 28717 | 8.46E-22 |
| *O. sinensis* | 89.412 | 85 | 5 | 3 | 109708 | 109789 | 29502 | 29419 | 8.46E-22 |
| *O. sinensis* | 90.361 | 83 | 2 | 1 | 28720 | 28802 | 108691 | 108615 | 8.46E-22 |
| *O. sinensis* | 89.412 | 85 | 5 | 3 | 29419 | 29502 | 109789 | 109708 | 8.46E-22 |
| *O. sinensis* | 92 | 75 | 5 | 1 | 28156 | 28229 | 143426 | 143352 | 8.46E-22 |
| *O. sinensis* | 85.859 | 99 | 8 | 3 | 129102 | 129200 | 44276 | 44368 | 1.09E-20 |
| *O. sinensis* | 95.312 | 64 | 2 | 1 | 150168 | 150231 | 94186 | 94248 | 1.09E-20 |
| *O. sinensis* | 85.859 | 99 | 8 | 3 | 44276 | 44368 | 129102 | 129200 | 1.09E-20 |
| *O. sinensis* | 95.312 | 64 | 2 | 1 | 94186 | 94248 | 150168 | 150231 | 1.09E-20 |
| *O. sinensis* | 91.781 | 73 | 3 | 3 | 92709 | 92779 | 14417 | 14346 | 3.93E-20 |
| *O. sinensis* | 91.781 | 73 | 3 | 3 | 14346 | 14417 | 92779 | 92709 | 3.93E-20 |
| *O. sinensis* | 95.082 | 61 | 3 | 0 | 29320 | 29380 | 28997 | 29057 | 1.41E-19 |
| *O. sinensis* | 95.082 | 61 | 3 | 0 | 28997 | 29057 | 29320 | 29380 | 1.41E-19 |
| *O. sinensis* | 98.148 | 54 | 0 | 1 | 108100 | 108152 | 14143 | 14090 | 1.83E-18 |
| *O. sinensis* | 87.952 | 83 | 4 | 1 | 135105 | 135181 | 28799 | 28717 | 1.83E-18 |
| *O. sinensis* | 96.491 | 57 | 1 | 1 | 27546 | 27601 | 113560 | 113504 | 1.83E-18 |
| *O. sinensis* | 88.75 | 80 | 3 | 1 | 28720 | 28799 | 135178 | 135105 | 1.83E-18 |
| *O. sinensis* | 90.278 | 72 | 4 | 2 | 109709 | 109779 | 28703 | 28772 | 6.58E-18 |
| *O. sinensis* | 90.278 | 72 | 4 | 2 | 28703 | 28772 | 109709 | 109779 | 6.58E-18 |
| *O. sinensis* | 96.364 | 55 | 1 | 1 | 143390 | 143444 | 55725 | 55778 | 2.37E-17 |
| *O. sinensis* | 94.828 | 58 | 2 | 1 | 139024 | 139080 | 110197 | 110254 | 2.37E-17 |
| *O. sinensis* | 92.308 | 65 | 1 | 4 | 55856 | 55918 | 120776 | 120838 | 2.37E-17 |
| *O. sinensis* | 94.828 | 58 | 2 | 1 | 110197 | 110254 | 139024 | 139080 | 2.37E-17 |
| *O. sinensis* | 98 | 50 | 1 | 0 | 21226 | 21275 | 139080 | 139031 | 8.52E-17 |
| *O. sinensis* | 88.732 | 71 | 6 | 1 | 110176 | 110244 | 24374 | 24444 | 3.06E-16 |
| *O. sinensis* | 94.545 | 55 | 3 | 0 | 63659 | 63713 | 63410 | 63464 | 3.06E-16 |
| *O. sinensis* | 94.545 | 55 | 3 | 0 | 63410 | 63464 | 63659 | 63713 | 3.06E-16 |
| *O. sinensis* | 88.732 | 71 | 6 | 1 | 24374 | 24444 | 110176 | 110244 | 3.06E-16 |
| *O. sinensis* | 90.769 | 65 | 5 | 1 | 150558 | 150622 | 110181 | 110244 | 3.06E-16 |
| *O. sinensis* | 90.769 | 65 | 5 | 1 | 156057 | 156121 | 110181 | 110244 | 3.06E-16 |
| *O. sinensis* | 100 | 46 | 0 | 0 | 135105 | 135150 | 128969 | 128924 | 3.06E-16 |
| *O. sinensis* | 100 | 46 | 0 | 0 | 128924 | 128969 | 135150 | 135105 | 3.06E-16 |
| *O. sinensis* | 90.769 | 65 | 5 | 1 | 110181 | 110244 | 150558 | 150622 | 3.06E-16 |
| *O. sinensis* | 90.769 | 65 | 5 | 1 | 110181 | 110244 | 156057 | 156121 | 3.06E-16 |
| *O. sinensis* | 80.165 | 121 | 16 | 5 | 60695 | 60809 | 6975 | 7093 | 1.10E-15 |
| *O. sinensis* | 97.917 | 48 | 1 | 0 | 55848 | 55895 | 17514 | 17467 | 1.10E-15 |
| *O. sinensis* | 97.917 | 48 | 1 | 0 | 55848 | 55895 | 27081 | 27034 | 1.10E-15 |
| *O. sinensis* | 90.625 | 64 | 5 | 1 | 143350 | 143412 | 28700 | 28763 | 1.10E-15 |
| *O. sinensis* | 97.917 | 48 | 1 | 0 | 27034 | 27081 | 55895 | 55848 | 1.10E-15 |
| *O. sinensis* | 97.917 | 48 | 1 | 0 | 17467 | 17514 | 55895 | 55848 | 1.10E-15 |
| *O. sinensis* | 80.165 | 121 | 16 | 6 | 6975 | 7093 | 60695 | 60809 | 1.10E-15 |
| *O. sinensis* | 91.935 | 62 | 1 | 4 | 108994 | 109053 | 70384 | 70443 | 1.10E-15 |
| *O. sinensis* | 97.917 | 48 | 1 | 0 | 98467 | 98514 | 98226 | 98179 | 1.10E-15 |
| *O. sinensis* | 97.917 | 48 | 1 | 0 | 98179 | 98226 | 98514 | 98467 | 1.10E-15 |
| *O. sinensis* | 87.671 | 73 | 7 | 2 | 120774 | 120845 | 108713 | 108642 | 1.10E-15 |
| *O. sinensis* | 91.935 | 62 | 1 | 4 | 70384 | 70443 | 108994 | 109053 | 1.10E-15 |
| *O. sinensis* | 93.22 | 59 | 1 | 3 | 135094 | 135150 | 109060 | 109003 | 1.10E-15 |
| *O. sinensis* | 89.552 | 67 | 5 | 2 | 108648 | 108713 | 120839 | 120774 | 1.10E-15 |
| *O. sinensis* | 94.545 | 55 | 1 | 2 | 27547 | 27600 | 120838 | 120785 | 1.10E-15 |
| *O. sinensis* | 93.22 | 59 | 1 | 3 | 109003 | 109060 | 135150 | 135094 | 1.10E-15 |
| *O. sinensis* | 90.625 | 64 | 5 | 1 | 28700 | 28763 | 143350 | 143412 | 1.10E-15 |
| *O. sinensis* | 84.615 | 91 | 4 | 5 | 141159 | 141246 | 13808 | 13725 | 3.96E-15 |
| *O. sinensis* | 92.857 | 56 | 2 | 2 | 127140 | 127194 | 9439 | 9493 | 1.43E-14 |
| *O. sinensis* | 97.872 | 47 | 0 | 1 | 28718 | 28764 | 27591 | 27546 | 1.43E-14 |
| *O. sinensis* | 94.34 | 53 | 2 | 1 | 27546 | 27597 | 28764 | 28712 | 1.43E-14 |
| *O. sinensis* | 97.872 | 47 | 0 | 1 | 55874 | 55919 | 28718 | 28764 | 1.43E-14 |
| *O. sinensis* | 97.872 | 47 | 0 | 1 | 28718 | 28764 | 55874 | 55919 | 1.43E-14 |
| *O. sinensis* | 97.826 | 46 | 1 | 0 | 58034 | 58079 | 57998 | 58043 | 1.43E-14 |
| *O. sinensis* | 97.826 | 46 | 1 | 0 | 57998 | 58043 | 58034 | 58079 | 1.43E-14 |
| *O. sinensis* | 91.525 | 59 | 3 | 2 | 139024 | 139080 | 110048 | 110106 | 1.43E-14 |
| *O. sinensis* | 95.918 | 49 | 1 | 1 | 28718 | 28766 | 113515 | 113562 | 5.13E-14 |
| *O. sinensis* | 88.235 | 68 | 3 | 5 | 113470 | 113536 | 17530 | 17467 | 1.84E-13 |
| *O. sinensis* | 97.778 | 45 | 0 | 1 | 87494 | 87538 | 28679 | 28722 | 1.84E-13 |
| *O. sinensis* | 95.745 | 47 | 2 | 0 | 70392 | 70438 | 28753 | 28799 | 1.84E-13 |
| *O. sinensis* | 95.745 | 47 | 2 | 0 | 28753 | 28799 | 70392 | 70438 | 1.84E-13 |
| *O. sinensis* | 97.778 | 45 | 0 | 1 | 28679 | 28722 | 87494 | 87538 | 1.84E-13 |
| *O. sinensis* | 88.06 | 67 | 5 | 3 | 113495 | 113560 | 108648 | 108712 | 1.84E-13 |
| *O. sinensis* | 97.727 | 44 | 1 | 0 | 143370 | 143413 | 108691 | 108648 | 1.84E-13 |
| *O. sinensis* | 88.235 | 68 | 3 | 5 | 17467 | 17530 | 113536 | 113470 | 1.84E-13 |
| *O. sinensis* | 95.745 | 47 | 2 | 0 | 108648 | 108694 | 143413 | 143367 | 1.84E-13 |
| *O. sinensis* | 85 | 80 | 5 | 3 | 140308 | 140387 | 13808 | 13736 | 6.63E-13 |
| *O. sinensis* | 94.118 | 51 | 0 | 3 | 128925 | 128975 | 28757 | 28804 | 6.63E-13 |
| *O. sinensis* | 97.727 | 44 | 0 | 1 | 128926 | 128969 | 70396 | 70438 | 6.63E-13 |
| *O. sinensis* | 87.692 | 65 | 7 | 1 | 143362 | 143426 | 120788 | 120851 | 6.63E-13 |
| *O. sinensis* | 94.118 | 51 | 0 | 3 | 28757 | 28804 | 128925 | 128975 | 6.63E-13 |
| *O. sinensis* | 97.727 | 44 | 0 | 1 | 70396 | 70438 | 128926 | 128969 | 6.63E-13 |
| *O. sinensis* | 87.692 | 65 | 7 | 1 | 120788 | 120851 | 143362 | 143426 | 6.63E-13 |
| *O. sinensis* | 81.308 | 107 | 5 | 9 | 126069 | 126166 | 13825 | 13725 | 2.38E-12 |
| *O. sinensis* | 93.878 | 49 | 2 | 1 | 113488 | 113536 | 27081 | 27034 | 2.38E-12 |
| *O. sinensis* | 92.308 | 52 | 3 | 1 | 120788 | 120838 | 28712 | 28763 | 2.38E-12 |
| *O. sinensis* | 93.878 | 49 | 2 | 1 | 27034 | 27081 | 113536 | 113488 | 2.38E-12 |
| *O. sinensis* | 92.308 | 52 | 3 | 1 | 28712 | 28763 | 120788 | 120838 | 2.38E-12 |
| *O. sinensis* | 88.333 | 60 | 5 | 1 | 138775 | 138832 | 12906 | 12965 | 8.58E-12 |
| *O. sinensis* | 100 | 38 | 0 | 0 | 66898 | 66935 | 34128 | 34165 | 8.58E-12 |
| *O. sinensis* | 100 | 38 | 0 | 0 | 34128 | 34165 | 66898 | 66935 | 8.58E-12 |
| *O. sinensis* | 88.333 | 60 | 5 | 1 | 12906 | 12965 | 138775 | 138832 | 8.58E-12 |
| *O. sinensis* | 82.716 | 81 | 13 | 1 | 148114 | 148193 | 146764 | 146844 | 8.58E-12 |
| *O. sinensis* | 82.716 | 81 | 13 | 1 | 146764 | 146844 | 148114 | 148193 | 8.58E-12 |
| *O. sinensis* | 85.507 | 69 | 7 | 2 | 29429 | 29497 | 28772 | 28707 | 3.08E-11 |
| *O. sinensis* | 84.722 | 72 | 8 | 2 | 28707 | 28775 | 29497 | 29426 | 3.08E-11 |
| *O. sinensis* | 90.566 | 53 | 3 | 2 | 18819 | 18870 | 113500 | 113551 | 3.08E-11 |
