# Supplementary material for: Comparative analysis of the complete mitochondrial genomes of four cordyceps fungi
Source: Ecol Evol. 2022 Apr 25;12(4):e8818. doi: 10.1002/ece3.8818 (PMC9036042; doi:10.1002/ece3.8818)
Supplement: Supplementary file 4 — Table S4 [file ECE3-12-e8818-s005.docx]

Table S4 Tandem repeats detected in the mitogenomes of cordyceps using the Tandem Repeats Finder program

| Indices | PeriodSize | CopyNumber | ConsensusSize | PercentMatches | PercentIndels | Score | Entropy(0-2) | Repeat motif |
| --- | --- | --- | --- | --- | --- | --- | --- | --- |
| ***C. brongniartii*** | | | | | | | | |
| 21523--21630 | 48 | 2.3 | 47 | 93 | 4 | 189 | 1.66 | TAAAAATTATTGCACAAAATATGAACATTTATACTTACATTTTTCAA |
| 21809--21903 | 27 | 3.5 | 27 | 89 | 0 | 118 | 1.98 | GTTGAACCTAGACCAACTACCCCTATG |
| 22153--22250 | 45 | 2.2 | 45 | 100 | 0 | 196 | 1.89 | TCAAAGTACTATTATGCAAAATGTAGCTGAATCTAGTTTTCAAGC |
| 22221--22384 | 51 | 3.2 | 50 | 92 | 3 | 256 | 1.94 | TAGCTGAATCTAGTTCTCAAGCTCAAAGTAATATGCCTGAGATGAGAGTA |
| 29131--29161 | 16 | 1.9 | 16 | 93 | 0 | 53 | 1.12 | ATATAAATATAATAAG |
| 29138--29174 | 19 | 1.9 | 19 | 88 | 0 | 56 | 1.2 | TATAATAAGAAATAAATAT |
| 4845--4880 | 18 | 2 | 18 | 94 | 0 | 63 | 1.31 | AATAAATTAACTTTATTA |
| ***C. militaris*** | | | | | | | | |
| 11924--11956 | 10 | 3.2 | 11 | 83 | 16 | 52 | 1.6 | ATACAAGTTAT |
| 12957--13080 | 62 | 2 | 62 | 100 | 0 | 248 | 1.81 | TATTGAAGATGAGTGCTGCAAGAGGTTATTCTGTAGATATATTAGAAAAATGTGACTTAGAA |
| 1660--1695 | 19 | 1.9 | 18 | 94 | 5 | 63 | 1.31 | AAAATTATATTATTATTC |
| 6761--6796 | 18 | 2 | 18 | 94 | 0 | 63 | 1.31 | AATAAATTAACTTTATTA |
| 6773--6811 | 21 | 2 | 19 | 85 | 14 | 53 | 1.14 | TTATTAAATAAATTATTTA |
| ***O. sinensis*** | | | | | | | | |
| 121537--121572 | 5 | 7.2 | 5 | 100 | 0 | 72 | 0.96 | TGGTT |
| 121938--121972 | 5 | 7.2 | 5 | 93 | 6 | 63 | 0.97 | TGGTT |
| 126024--126052 | 6 | 4.8 | 6 | 100 | 0 | 58 | 0.89 | AAAATT |
| 135320--135356 | 19 | 1.9 | 19 | 88 | 0 | 56 | 1.98 | TCTACGAAGACTTCGTAGC |
| 14087--14174 | 31 | 2.8 | 31 | 100 | 0 | 176 | 1.67 | CTGCTGCTGCTTGCATATTTTTTTCTTCTTA |
| 146549--146584 | 5 | 7.2 | 5 | 100 | 0 | 72 | 0.96 | TGGTT |
| 147842--147866 | 12 | 2.1 | 12 | 100 | 0 | 50 | 1.58 | ATTATGATGGAG |
| 150172--150208 | 19 | 1.9 | 19 | 88 | 0 | 56 | 1.98 | TCTACGAAGACTTCGTAGC |
| 155417--155476 | 19 | 3.2 | 19 | 90 | 6 | 95 | 0.92 | TGTTTGTTTTGTTTTGTAT |
| 155422--155476 | 5 | 11.2 | 5 | 90 | 3 | 85 | 0.94 | TTTTG |
| 156418--156454 | 19 | 1.9 | 19 | 88 | 0 | 56 | 1.98 | TCTACGAAGACTTCGTAGC |
| 19441--19477 | 18 | 2.1 | 18 | 90 | 5 | 58 | 1.59 | ATATATATATGCTTCTGT |
| 21382--21415 | 14 | 2.3 | 15 | 90 | 5 | 52 | 1.16 | AATATAAATTTATAT |
| 23492--23526 | 7 | 5 | 7 | 85 | 0 | 52 | 1.55 | GCGAAGC |
| 24573--24600 | 7 | 4 | 7 | 100 | 0 | 56 | 1.84 | GTTTCAC |
| 25777--25811 | 15 | 2.3 | 15 | 100 | 0 | 70 | 1.96 | GCTGCTATGAAATAA |
| 30835--30891 | 7 | 8.1 | 7 | 100 | 0 | 114 | 1.55 | CGCTTCG |
| 45473--45563 | 30 | 3 | 30 | 87 | 9 | 141 | 1.8 | CAAAATTCAATACGTCTTTTAGATACTATT |
| 48185--48220 | 19 | 1.9 | 19 | 88 | 5 | 56 | 1.94 | ACTTATGGCACAGGTAATG |
| 50433--50471 | 21 | 1.9 | 20 | 85 | 15 | 53 | 1.67 | TAATGCTATTATCTATAAAA |
| 55764--55799 | 7 | 5.1 | 7 | 100 | 0 | 72 | 1.55 | CGCTTCG |
| 55805--55838 | 6 | 5.7 | 6 | 96 | 0 | 59 | 0.99 | AAATTT |
| 56230--56266 | 19 | 1.9 | 19 | 88 | 0 | 56 | 1.98 | TCTACGAAGACTTCGTAGC |
| 57210--57241 | 15 | 2 | 17 | 88 | 11 | 50 | 1.54 | ATTATAAATATTGCATA |
| 57998--58079 | 36 | 2.3 | 36 | 97 | 0 | 155 | 1.96 | CTACAGGATCTGATACTAAAGGATCATCTGTTGATT |
| 58008--58081 | 21 | 3.8 | 21 | 69 | 20 | 70 | 1.94 | TGATACTAAAGGATCATCTGT |
| 58634--58662 | 13 | 2.2 | 13 | 100 | 0 | 58 | 1 | AAATATTATTATT |
| 63144--63288 | 72 | 2 | 72 | 94 | 2 | 256 | 1.87 | CCGATTCTCCTCAAGCTGGACCATATGCACCCTCACCCGATTCTCCTCAAGCTGGACCATCAGCA |
| 63416--63713 | 63 | 4.8 | 63 | 85 | 7 | 446 | 1.63 | GATAATGAAGATGTAAAAGACAAAGGTAAAGGAGTAGAAAAAGCAGAAAATAATACAGATACT |
| 63416--63750 | 123 | 2.7 | 123 | 92 | 4 | 523 | 1.68 | GATAATGAAGATGTAAAAGACAAAGGTAAAGGAGTAGAAAAAGAAGAAAGTGAATCGAATGCCCC |
| 67713--67748 | 7 | 5.1 | 7 | 100 | 0 | 72 | 1.36 | GCGGAGC |
| 71567--71612 | 18 | 2.6 | 18 | 100 | 0 | 92 | 1.7 | TAATAATCCACAACAGCC |
| 72395--72420 | 8 | 3.2 | 8 | 100 | 0 | 52 | 1.89 | GCTGCGTA |
| 75134--75176 | 19 | 2.3 | 18 | 81 | 14 | 52 | 1.67 | TATATTTCTACATTTAGA |
| 80010--80076 | 2 | 34 | 2 | 77 | 3 | 64 | 1.27 | TA |
| 83281--83366 | 27 | 3.2 | 27 | 91 | 0 | 145 | 1.84 | CAAGCGTGCTTCGCAGAAAAAAGATAA |
| 8422--8500 | 22 | 3.4 | 22 | 75 | 16 | 86 | 1.82 | TAGTATTAATGCTCAACAGTTA |
| 8528--8576 | 25 | 2 | 25 | 83 | 0 | 62 | 1.83 | TAATAGTATTAATACTCAACAGTTA |
| 86277--86312 | 7 | 5.1 | 7 | 96 | 0 | 63 | 1.88 | ACTTCGT |
| 9037--9086 | 24 | 2.1 | 24 | 88 | 0 | 73 | 1.55 | AAATATATATGATATCTAAGAACA |
| 94190--94226 | 19 | 1.9 | 19 | 88 | 0 | 56 | 1.97 | TATACGAAGACTTCGTAGC |
| 94318--94343 | 9 | 2.9 | 9 | 100 | 0 | 52 | 1.9 | AGCTTCGCG |
| 98109--98185 | 7 | 11 | 7 | 100 | 0 | 154 | 1.56 | GCGAAGC |
| ***O. xuefengensis*** | | | | | | | | |
| 27654--27691 | 19 | 2 | 20 | 85 | 15 | 53 | 1.51 | CTGTAGTTTTATTTTGCTTG |
| 43285--43361 | 22 | 3.4 | 22 | 79 | 10 | 91 | 1.78 | TAGTATTAATGCTAAACAGTTA |
| 43385--43436 | 25 | 2.1 | 25 | 92 | 0 | 86 | 1.78 | TTATAATAGTATTAATACTCAACAG |
| 43862--43911 | 24 | 2.1 | 24 | 92 | 0 | 82 | 1.57 | AAATATATTTGATATCTAAGAACA |
| 50244--50280 | 18 | 2.1 | 18 | 90 | 5 | 58 | 1.59 | ATATATATATGCTTCTGT |
| 66701--66739 | 21 | 1.9 | 20 | 85 | 15 | 53 | 1.67 | TAATGCTATTATCTATAAAA |
| 8353--8388 | 18 | 1.9 | 19 | 88 | 5 | 56 | 1.28 | CTTTAAATATATATTATTC |
