# Supplementary material for: Comparative analysis of the complete mitochondrial genomes of four cordyceps fungi
Source: Ecol Evol. 2022 Apr 25;12(4):e8818. doi: 10.1002/ece3.8818 (PMC9036042; doi:10.1002/ece3.8818)
Supplement: Supplementary file 5 — Table S5 [file ECE3-12-e8818-s001.docx]

Table S5 Distribution of repeat loci in the four cordyceps mitogenomes identified by REPuter.

| Species | 1st repeat length | Starting position | Match direction | 2nd repeat length | Starting position | E-value |
| --- | --- | --- | --- | --- | --- | --- |
| *C._brongniartii* | 85 | 22220 | F | 85 | 22271 | 5.77E-37 |
| *C._brongniartii* | 75 | 22236 | F | 75 | 22287 | 4.14E-31 |
| *C._brongniartii* | 71 | 6752 | F | 71 | 25982 | 1.24E-32 |
| *C._brongniartii* | 65 | 6759 | F | 65 | 15092 | 2.80E-25 |
| *C._brongniartii* | 59 | 23940 | F | 59 | 33156 | 8.55E-22 |
| *C._brongniartii* | 57 | 22152 | F | 57 | 22197 | 2.67E-24 |
| *C._brongniartii* | 56 | 21522 | F | 56 | 21570 | 8.64E-22 |
| *C._brongniartii* | 53 | 15092 | F | 53 | 25989 | 4.95E-20 |
| *C._brongniartii* | 51 | 6641 | F | 51 | 15110 | 3.59E-17 |
| *C._brongniartii* | 51 | 28953 | F | 51 | 33169 | 6.38E-23 |
| *C._brongniartii* | 50 | 22270 | F | 50 | 22321 | 1.35E-16 |
| *C._brongniartii* | 49 | 6780 | P | 49 | 28948 | 5.08E-16 |
| *C._brongniartii* | 47 | 21531 | F | 47 | 21579 | 2.30E-18 |
| *C._brongniartii* | 47 | 6776 | F | 47 | 26006 | 1.63E-20 |
| *C._brongniartii* | 47 | 15109 | F | 47 | 26006 | 7.16E-15 |
| *C._brongniartii* | 47 | 6641 | F | 47 | 6777 | 1.59E-16 |
| *C._brongniartii* | 46 | 21808 | F | 46 | 21835 | 2.68E-14 |
| *C._brongniartii* | 46 | 22220 | F | 46 | 22322 | 2.68E-14 |
| *C._brongniartii* | 46 | 6641 | F | 46 | 26007 | 6.09E-16 |
| *C._brongniartii* | 46 | 23953 | F | 46 | 28953 | 9.02E-18 |
| *C._brongniartii* | 46 | 26010 | P | 46 | 33167 | 9.02E-18 |
| *C._brongniartii* | 44 | 26010 | P | 44 | 28953 | 1.38E-16 |
| *C._brongniartii* | 44 | 23951 | P | 44 | 26012 | 1.38E-16 |
| *C._brongniartii* | 43 | 10622 | F | 43 | 26009 | 1.39E-12 |
| *C._brongniartii* | 43 | 6779 | F | 43 | 10622 | 1.39E-12 |
| *C._brongniartii* | 43 | 6780 | P | 43 | 33170 | 5.40E-16 |
| *C._brongniartii* | 43 | 15113 | P | 43 | 33170 | 3.40E-14 |
| *C._brongniartii* | 43 | 15113 | P | 43 | 28954 | 3.40E-14 |
| *C._brongniartii* | 42 | 10619 | P | 42 | 33175 | 5.19E-12 |
| *C._brongniartii* | 42 | 6645 | P | 42 | 33170 | 1.67E-17 |
| *C._brongniartii* | 42 | 10619 | P | 42 | 28959 | 5.19E-12 |
| *C._brongniartii* | 42 | 6645 | P | 42 | 28954 | 1.67E-17 |
| *C._brongniartii* | 41 | 6645 | F | 41 | 10624 | 1.93E-11 |
| *C._brongniartii* | 41 | 6646 | P | 41 | 23954 | 6.69E-17 |
| *C._brongniartii* | 41 | 6782 | P | 41 | 23954 | 8.23E-15 |
| *C._brongniartii* | 41 | 15115 | P | 41 | 23954 | 4.94E-13 |
| *C._brongniartii* | 40 | 10625 | P | 40 | 23955 | 7.14E-11 |
| *C._brongniartii* | 38 | 21830 | F | 38 | 21857 | 9.76E-10 |
| *C._brongniartii* | 37 | 21541 | F | 37 | 21589 | 1.71E-14 |
| *C._brongniartii* | 37 | 17902 | F | 37 | 19816 | 3.60E-09 |
| *C._brongniartii* | 35 | 6616 | P | 35 | 28991 | 2.88E-11 |
| *C._brongniartii* | 34 | 7265 | F | 34 | 11712 | 1.10E-12 |
| *C._brongniartii* | 34 | 22175 | F | 34 | 22322 | 5.54E-09 |
| *C._brongniartii* | 34 | 22175 | F | 34 | 22271 | 5.54E-09 |
| *C._brongniartii* | 33 | 23908 | F | 33 | 33123 | 2.08E-08 |
| *C._brongniartii* | 32 | 7298 | F | 32 | 11752 | 7.83E-08 |
| *C._brongniartii* | 32 | 10622 | F | 32 | 15112 | 1.68E-09 |
| *C._brongniartii* | 32 | 23963 | P | 32 | 26012 | 1.75E-11 |
| *C._brongniartii* | 31 | 23990 | F | 31 | 28992 | 2.94E-07 |
| *C._brongniartii* | 31 | 6619 | P | 31 | 23990 | 2.94E-07 |
| *C._brongniartii* | 31 | 10560 | P | 31 | 19927 | 8.52E-06 |
| *C._brongniartii* | 30 | 1000 | F | 30 | 24713 | 3.08E-05 |
| *C._brongniartii* | 30 | 912 | F | 30 | 24624 | 3.08E-05 |
| *C._brongniartii* | 30 | 6757 | F | 30 | 10599 | 2.53E-08 |
| *C._brongniartii* | 30 | 10599 | F | 30 | 25987 | 1.10E-06 |
| *C._brongniartii* | 30 | 22236 | F | 30 | 22338 | 1.10E-06 |
| *C._brongniartii* | 30 | 10599 | F | 30 | 15090 | 2.53E-08 |
| *C. militaris* | 117 | 753 | F | 117 | 2100 | 7.92E-56 |
| *C. militaris* | 109 | 761 | F | 109 | 2108 | 3.92E-53 |
| *C. militaris* | 91 | 779 | F | 91 | 2126 | 1.39E-44 |
| *C. militaris* | 62 | 12956 | F | 62 | 13018 | 1.46E-29 |
| *C. militaris* | 61 | 809 | F | 61 | 2156 | 5.86E-29 |
| *C. militaris* | 42 | 29209 | F | 42 | 29463 | 4.99E-12 |
| *C. militaris* | 35 | 2395 | F | 35 | 12436 | 4.66E-08 |
| *C. militaris* | 32 | 7467 | F | 32 | 31322 | 2.26E-06 |
| *C. militaris* | 32 | 5590 | F | 32 | 22475 | 7.54E-08 |
| *C. militaris* | 31 | 19102 | F | 31 | 32677 | 2.83E-07 |
| *C. militaris* | 31 | 1234 | F | 31 | 2601 | 8.20E-06 |
| *C. militaris* | 31 | 21196 | F | 31 | 31041 | 8.20E-06 |
| *C. militaris* | 30 | 667 | F | 30 | 2013 | 2.96E-05 |
| *C. militaris* | 30 | 5591 | F | 30 | 19379 | 2.96E-05 |
| *C. militaris* | 30 | 19379 | F | 30 | 22476 | 2.96E-05 |
| *C. militaris* | 30 | 29187 | F | 30 | 29441 | 2.96E-05 |
| *C. militaris* | 30 | 9546 | P | 30 | 9546 | 1.06E-06 |
| *O. sinensis* | 153 | 44903 | F | 153 | 70165 | 8.46E-76 |
| *O. sinensis* | 142 | 44914 | F | 142 | 70176 | 2.02E-71 |
| *O. sinensis* | 133 | 63494 | F | 133 | 63617 | 2.35E-68 |
| *O. sinensis* | 128 | 140262 | F | 128 | 141113 | 6.03E-68 |
| *O. sinensis* | 117 | 24401 | F | 117 | 150579 | 8.87E-59 |
| *O. sinensis* | 112 | 63415 | F | 112 | 63478 | 8.70E-56 |
| *O. sinensis* | 112 | 152062 | P | 112 | 155466 | 1.45E-53 |
| *O. sinensis* | 110 | 44793 | F | 110 | 70056 | 4.14E-57 |
| *O. sinensis* | 106 | 135277 | F | 106 | 156375 | 5.52E-48 |
| *O. sinensis* | 105 | 14120 | F | 105 | 147560 | 1.34E-51 |
| *O. sinensis* | 105 | 24448 | F | 105 | 156128 | 2.08E-49 |
| *O. sinensis* | 103 | 113371 | F | 103 | 120908 | 3.21E-48 |
| *O. sinensis* | 100 | 17420 | F | 100 | 26987 | 4.34E-51 |
| *O. sinensis* | 97 | 24456 | F | 97 | 156136 | 8.09E-47 |
| *O. sinensis* | 96 | 63431 | F | 96 | 63617 | 4.56E-44 |
| *O. sinensis* | 96 | 14135 | P | 96 | 108042 | 3.20E-46 |
| *O. sinensis* | 95 | 108617 | F | 95 | 135104 | 1.66E-41 |
| *O. sinensis* | 93 | 55685 | F | 93 | 120908 | 2.74E-42 |
| *O. sinensis* | 93 | 55685 | F | 93 | 113371 | 2.74E-42 |
| *O. sinensis* | 91 | 55464 | F | 91 | 150034 | 1.14E-45 |
| *O. sinensis* | 90 | 56204 | F | 90 | 156392 | 1.64E-40 |
| *O. sinensis* | 90 | 108048 | P | 90 | 147575 | 4.55E-45 |
| *O. sinensis* | 89 | 121510 | F | 89 | 146522 | 4.86E-42 |
| *O. sinensis* | 89 | 17397 | P | 89 | 135317 | 1.82E-44 |
| *O. sinensis* | 87 | 126571 | P | 87 | 151469 | 7.61E-41 |
| *O. sinensis* | 87 | 56910 | P | 87 | 150167 | 2.92E-43 |
| *O. sinensis* | 86 | 56930 | P | 86 | 156394 | 3.22E-36 |
| *O. sinensis* | 86 | 56930 | P | 86 | 135296 | 1.17E-42 |
| *O. sinensis* | 83 | 55695 | F | 83 | 113381 | 1.86E-38 |
| *O. sinensis* | 83 | 18774 | F | 83 | 25252 | 1.86E-38 |
| *O. sinensis* | 83 | 126080 | F | 83 | 141159 | 7.46E-41 |
| *O. sinensis* | 83 | 94165 | F | 83 | 135295 | 1.85E-34 |
| *O. sinensis* | 83 | 55678 | P | 83 | 109811 | 2.29E-36 |
| *O. sinensis* | 82 | 56212 | F | 82 | 156400 | 7.34E-38 |
| *O. sinensis* | 82 | 22992 | F | 82 | 108040 | 7.34E-38 |
| *O. sinensis* | 82 | 126080 | F | 82 | 140308 | 2.99E-40 |
| *O. sinensis* | 82 | 26971 | P | 82 | 156415 | 7.14E-34 |
| *O. sinensis* | 82 | 56934 | P | 82 | 94166 | 7.14E-34 |
| *O. sinensis* | 81 | 23000 | F | 81 | 56353 | 2.75E-33 |
| *O. sinensis* | 80 | 14151 | P | 80 | 22994 | 4.78E-39 |
| *O. sinensis* | 79 | 150555 | F | 79 | 156054 | 1.91E-38 |
| *O. sinensis* | 78 | 56938 | P | 78 | 156394 | 2.07E-33 |
| *O. sinensis* | 77 | 26074 | F | 77 | 147535 | 7.06E-35 |
| *O. sinensis* | 76 | 19871 | F | 76 | 24726 | 1.22E-36 |
| *O. sinensis* | 76 | 28153 | F | 76 | 157207 | 2.79E-34 |
| *O. sinensis* | 76 | 56204 | F | 76 | 135294 | 2.32E-30 |
| *O. sinensis* | 76 | 109811 | P | 76 | 120908 | 3.14E-32 |
| *O. sinensis* | 76 | 109811 | P | 76 | 113371 | 3.14E-32 |
| *O. sinensis* | 76 | 98109 | R | 76 | 98109 | 1.22E-36 |
| *O. sinensis* | 75 | 94165 | F | 75 | 156393 | 8.92E-30 |
| *O. sinensis* | 75 | 56351 | P | 75 | 147592 | 1.22E-31 |
| *O. sinensis* | 74 | 23000 | P | 74 | 147591 | 4.34E-33 |
| *O. sinensis* | 74 | 56206 | P | 74 | 56942 | 3.42E-29 |
| *O. sinensis* | 73 | 45233 | F | 73 | 70609 | 1.31E-28 |
| *O. sinensis* | 73 | 56353 | F | 73 | 108048 | 1.85E-30 |
| *O. sinensis* | 73 | 56221 | F | 73 | 156409 | 7.83E-35 |
| *O. sinensis* | 73 | 120912 | F | 73 | 150050 | 1.31E-28 |
| *O. sinensis* | 73 | 55480 | F | 73 | 120912 | 1.31E-28 |
| *O. sinensis* | 73 | 14152 | P | 73 | 56353 | 1.71E-32 |
| *O. sinensis* | 72 | 56212 | F | 72 | 135302 | 5.04E-28 |
| *O. sinensis* | 72 | 24335 | F | 72 | 156013 | 7.20E-30 |
| *O. sinensis* | 72 | 113369 | F | 72 | 150044 | 5.04E-28 |
| *O. sinensis* | 72 | 55474 | F | 72 | 113369 | 5.04E-28 |
| *O. sinensis* | 72 | 56221 | F | 72 | 135311 | 5.04E-28 |
| *O. sinensis* | 72 | 45247 | F | 72 | 70623 | 5.04E-28 |
| *O. sinensis* | 72 | 17212 | P | 72 | 148776 | 5.04E-28 |
| *O. sinensis* | 71 | 14350 | F | 71 | 23750 | 1.25E-33 |
| *O. sinensis* | 71 | 17522 | F | 71 | 156127 | 2.80E-29 |
| *O. sinensis* | 71 | 17523 | F | 71 | 150626 | 1.93E-27 |
| *O. sinensis* | 71 | 56221 | P | 71 | 56930 | 1.93E-27 |
| *O. sinensis* | 71 | 98108 | R | 71 | 98108 | 1.25E-33 |
| *O. sinensis* | 70 | 113404 | F | 70 | 120941 | 5.01E-33 |
| *O. sinensis* | 70 | 98108 | F | 70 | 98115 | 5.01E-33 |
| *O. sinensis* | 70 | 55685 | F | 70 | 150046 | 7.40E-27 |
| *O. sinensis* | 70 | 55476 | F | 70 | 55685 | 7.40E-27 |
| *O. sinensis* | 70 | 26146 | F | 70 | 147605 | 5.01E-33 |
| *O. sinensis* | 70 | 150626 | F | 70 | 156128 | 1.05E-30 |
| *O. sinensis* | 70 | 109817 | P | 70 | 150046 | 7.40E-27 |
| *O. sinensis* | 70 | 55476 | P | 70 | 109817 | 7.40E-27 |
| *O. sinensis* | 69 | 26082 | F | 69 | 147543 | 2.00E-32 |
| *O. sinensis* | 69 | 45142 | F | 69 | 70495 | 4.15E-30 |
| *O. sinensis* | 68 | 23001 | F | 68 | 148777 | 1.64E-27 |
| *O. sinensis* | 68 | 56354 | F | 68 | 148777 | 1.08E-25 |
| *O. sinensis* | 68 | 108049 | F | 68 | 148777 | 1.64E-27 |
| *O. sinensis* | 68 | 63143 | F | 68 | 63215 | 1.08E-25 |
| *O. sinensis* | 68 | 17215 | F | 68 | 147596 | 1.63E-29 |
| *O. sinensis* | 68 | 14156 | F | 68 | 17215 | 1.64E-27 |
| *O. sinensis* | 68 | 147596 | P | 68 | 148777 | 1.64E-27 |
| *O. sinensis* | 68 | 14156 | P | 68 | 148777 | 1.64E-27 |
| *O. sinensis* | 68 | 55854 | P | 68 | 120771 | 1.08E-25 |
| *O. sinensis* | 68 | 17215 | P | 68 | 108049 | 1.63E-29 |
| *O. sinensis* | 68 | 17215 | P | 68 | 56354 | 1.08E-25 |
| *O. sinensis* | 68 | 24867 | P | 68 | 27372 | 1.63E-29 |
| *O. sinensis* | 68 | 17215 | P | 68 | 23001 | 1.64E-27 |
| *O. sinensis* | 67 | 55695 | F | 67 | 150056 | 4.15E-25 |
| *O. sinensis* | 67 | 55486 | F | 67 | 55695 | 4.15E-25 |
| *O. sinensis* | 67 | 135315 | F | 67 | 150167 | 3.21E-31 |
| *O. sinensis* | 67 | 56225 | F | 67 | 150167 | 4.15E-25 |
| *O. sinensis* | 67 | 150167 | F | 67 | 156413 | 4.15E-25 |
| *O. sinensis* | 67 | 24341 | F | 67 | 126886 | 4.15E-25 |
| *O. sinensis* | 67 | 26986 | P | 67 | 56227 | 4.15E-25 |
| *O. sinensis* | 66 | 110030 | F | 66 | 150556 | 1.58E-24 |
| *O. sinensis* | 66 | 110030 | F | 66 | 156055 | 1.58E-24 |
| *O. sinensis* | 66 | 126886 | F | 66 | 156019 | 1.58E-24 |
| *O. sinensis* | 66 | 17420 | P | 66 | 156415 | 1.58E-24 |
| *O. sinensis* | 66 | 26987 | P | 66 | 135317 | 1.28E-30 |
| *O. sinensis* | 66 | 55695 | P | 66 | 109811 | 2.54E-28 |
| *O. sinensis* | 66 | 17420 | P | 66 | 56227 | 1.58E-24 |
| *O. sinensis* | 65 | 28164 | F | 65 | 157218 | 5.13E-30 |
| *O. sinensis* | 65 | 26988 | F | 65 | 56930 | 5.13E-30 |
| *O. sinensis* | 65 | 17421 | F | 65 | 56930 | 5.13E-30 |
| *O. sinensis* | 65 | 26988 | P | 65 | 150169 | 5.13E-30 |
| *O. sinensis* | 65 | 17421 | P | 65 | 150169 | 5.13E-30 |
| *O. sinensis* | 65 | 70332 | P | 65 | 135152 | 6.05E-24 |
| *O. sinensis* | 64 | 27608 | F | 64 | 109877 | 3.94E-27 |
| *O. sinensis* | 64 | 18793 | F | 64 | 25271 | 2.05E-29 |
| *O. sinensis* | 64 | 55845 | P | 64 | 120784 | 2.31E-23 |
| *O. sinensis* | 63 | 17531 | F | 63 | 150634 | 1.44E-24 |
| *O. sinensis* | 63 | 98108 | F | 63 | 98122 | 8.21E-29 |
| *O. sinensis* | 63 | 94185 | F | 63 | 150167 | 8.80E-23 |
| *O. sinensis* | 63 | 143350 | P | 63 | 157221 | 8.80E-23 |
| *O. sinensis* | 62 | 150634 | F | 62 | 156136 | 3.28E-28 |
| *O. sinensis* | 62 | 17531 | F | 62 | 24456 | 3.35E-22 |
| *O. sinensis* | 62 | 110055 | F | 62 | 110203 | 3.28E-28 |
| *O. sinensis* | 62 | 120856 | P | 62 | 157125 | 6.10E-26 |
| *O. sinensis* | 62 | 28167 | P | 62 | 143351 | 3.35E-22 |
| *O. sinensis* | 62 | 26146 | P | 62 | 56351 | 6.10E-26 |
| *O. sinensis* | 61 | 55858 | F | 61 | 113499 | 2.40E-25 |
| *O. sinensis* | 61 | 28996 | F | 61 | 29319 | 1.28E-21 |
| *O. sinensis* | 61 | 56955 | P | 61 | 156394 | 1.31E-27 |
| *O. sinensis* | 61 | 113499 | P | 61 | 120774 | 2.16E-23 |
| *O. sinensis* | 61 | 55491 | P | 61 | 109811 | 1.28E-21 |
| *O. sinensis* | 61 | 26992 | P | 61 | 94187 | 1.28E-21 |
| *O. sinensis* | 61 | 17425 | P | 61 | 94187 | 1.28E-21 |
| *O. sinensis* | 61 | 14164 | P | 61 | 56353 | 1.31E-27 |
| *O. sinensis* | 61 | 27531 | P | 61 | 55872 | 2.40E-25 |
| *O. sinensis* | 60 | 45217 | F | 60 | 70593 | 4.85E-21 |
| *O. sinensis* | 60 | 55718 | F | 60 | 113404 | 5.25E-27 |
| *O. sinensis* | 60 | 14165 | F | 60 | 26146 | 9.45E-25 |
| *O. sinensis* | 60 | 26146 | P | 60 | 108048 | 5.25E-27 |
| *O. sinensis* | 60 | 17223 | P | 60 | 56354 | 8.37E-23 |
| *O. sinensis* | 60 | 23000 | P | 60 | 26146 | 9.45E-25 |
| *O. sinensis* | 59 | 120963 | F | 59 | 143406 | 2.10E-26 |
| *O. sinensis* | 59 | 56221 | F | 59 | 94181 | 1.84E-20 |
| *O. sinensis* | 59 | 17224 | F | 59 | 26146 | 3.72E-24 |
| *O. sinensis* | 59 | 120910 | P | 59 | 157071 | 2.10E-26 |
| *O. sinensis* | 59 | 26146 | P | 59 | 148777 | 3.23E-22 |
| *O. sinensis* | 58 | 30352 | F | 58 | 38962 | 7.00E-20 |
| *O. sinensis* | 58 | 111760 | F | 58 | 128851 | 1.46E-23 |
| *O. sinensis* | 58 | 30834 | P | 58 | 98107 | 8.40E-26 |
| *O. sinensis* | 57 | 14086 | F | 57 | 14117 | 3.36E-25 |
| *O. sinensis* | 57 | 94191 | F | 57 | 135321 | 4.83E-21 |
| *O. sinensis* | 57 | 56231 | F | 57 | 94191 | 2.66E-19 |
| *O. sinensis* | 57 | 94191 | F | 57 | 156419 | 2.66E-19 |
| *O. sinensis* | 57 | 122789 | F | 57 | 147595 | 4.83E-21 |
| *O. sinensis* | 57 | 14155 | F | 57 | 122789 | 2.66E-19 |
| *O. sinensis* | 57 | 109826 | F | 57 | 157071 | 5.75E-23 |
| *O. sinensis* | 57 | 28705 | P | 57 | 157222 | 2.66E-19 |
| *O. sinensis* | 57 | 113375 | P | 57 | 157071 | 5.75E-23 |
| *O. sinensis* | 57 | 55480 | P | 57 | 157071 | 4.83E-21 |
| *O. sinensis* | 57 | 150050 | P | 57 | 157071 | 4.83E-21 |
| *O. sinensis* | 57 | 55689 | P | 57 | 157071 | 5.75E-23 |
| *O. sinensis* | 57 | 26996 | P | 57 | 156415 | 4.83E-21 |
| *O. sinensis* | 57 | 17429 | P | 57 | 156415 | 4.83E-21 |
| *O. sinensis* | 57 | 16471 | P | 57 | 141186 | 4.83E-21 |
| *O. sinensis* | 57 | 108061 | P | 57 | 122789 | 4.83E-21 |
| *O. sinensis* | 57 | 23013 | P | 57 | 122789 | 2.66E-19 |
| *O. sinensis* | 57 | 55865 | P | 57 | 120771 | 4.83E-21 |
| *O. sinensis* | 57 | 30834 | P | 57 | 98122 | 3.36E-25 |
| *O. sinensis* | 57 | 30834 | P | 57 | 98115 | 3.36E-25 |
| *O. sinensis* | 57 | 26996 | P | 57 | 56227 | 4.83E-21 |
| *O. sinensis* | 57 | 17429 | P | 57 | 56227 | 4.83E-21 |
| *O. sinensis* | 57 | 28168 | P | 57 | 28705 | 2.66E-19 |
| *O. sinensis* | 57 | 30834 | R | 57 | 30834 | 3.36E-25 |
| *O. sinensis* | 57 | 30834 | C | 57 | 98108 | 3.36E-25 |
| *O. sinensis* | 57 | 30834 | C | 57 | 98115 | 3.36E-25 |
| *O. sinensis* | 57 | 30834 | C | 57 | 98122 | 3.36E-25 |
| *O. sinensis* | 56 | 126029 | F | 56 | 141110 | 2.26E-22 |
| *O. sinensis* | 56 | 23018 | F | 56 | 108066 | 1.34E-24 |
| *O. sinensis* | 56 | 98108 | F | 56 | 98129 | 1.34E-24 |
| *O. sinensis* | 56 | 17215 | F | 56 | 122790 | 2.26E-22 |
| *O. sinensis* | 56 | 45000 | F | 56 | 70262 | 2.26E-22 |
| *O. sinensis* | 56 | 23018 | P | 56 | 147591 | 1.34E-24 |
| *O. sinensis* | 56 | 16472 | P | 56 | 126107 | 1.86E-20 |
| *O. sinensis* | 56 | 30835 | P | 56 | 98129 | 1.34E-24 |
| *O. sinensis* | 56 | 30834 | C | 56 | 98129 | 1.34E-24 |
| *O. sinensis* | 55 | 56212 | F | 55 | 94172 | 3.81E-18 |
| *O. sinensis* | 55 | 56371 | F | 55 | 108066 | 8.87E-22 |
| *O. sinensis* | 55 | 28158 | F | 55 | 55845 | 3.81E-18 |
| *O. sinensis* | 55 | 63184 | F | 55 | 63220 | 7.19E-20 |
| *O. sinensis* | 55 | 24401 | F | 55 | 156078 | 5.38E-24 |
| *O. sinensis* | 55 | 56371 | P | 55 | 147592 | 8.87E-22 |
| *O. sinensis* | 54 | 24499 | F | 54 | 156179 | 2.15E-23 |
| *O. sinensis* | 54 | 14089 | F | 54 | 147560 | 2.15E-23 |
| *O. sinensis* | 54 | 121893 | F | 54 | 146504 | 1.44E-17 |
| *O. sinensis* | 54 | 143359 | P | 54 | 157221 | 2.77E-19 |
| *O. sinensis* | 54 | 28153 | P | 54 | 120799 | 1.44E-17 |
| *O. sinensis* | 53 | 27605 | F | 53 | 157124 | 5.44E-17 |
| *O. sinensis* | 53 | 126032 | F | 53 | 140262 | 1.37E-20 |
| *O. sinensis* | 53 | 92921 | F | 53 | 123742 | 1.07E-18 |
| *O. sinensis* | 53 | 28709 | F | 53 | 143359 | 5.44E-17 |
| *O. sinensis* | 53 | 108132 | P | 53 | 147527 | 1.37E-20 |
| *O. sinensis* | 52 | 14120 | F | 52 | 26099 | 3.44E-22 |
| *O. sinensis* | 52 | 14089 | F | 52 | 26099 | 3.44E-22 |
| *O. sinensis* | 52 | 109989 | F | 52 | 156014 | 4.11E-18 |
| *O. sinensis* | 52 | 24336 | F | 52 | 109989 | 4.11E-18 |
| *O. sinensis* | 52 | 17232 | P | 52 | 148776 | 4.11E-18 |
| *O. sinensis* | 52 | 56371 | P | 52 | 122789 | 2.05E-16 |
| *O. sinensis* | 52 | 27606 | P | 52 | 120866 | 4.11E-18 |
| *O. sinensis* | 52 | 27540 | P | 52 | 55872 | 3.44E-22 |
| *O. sinensis* | 51 | 45160 | F | 51 | 70513 | 1.38E-21 |
| *O. sinensis* | 51 | 23018 | F | 51 | 148794 | 2.11E-19 |
| *O. sinensis* | 51 | 56371 | F | 51 | 148794 | 1.58E-17 |
| *O. sinensis* | 51 | 108066 | F | 51 | 148794 | 2.11E-19 |
| *O. sinensis* | 51 | 120957 | P | 51 | 157029 | 2.11E-19 |
| *O. sinensis* | 51 | 113420 | P | 51 | 157029 | 2.11E-19 |
| *O. sinensis* | 51 | 109836 | P | 51 | 150046 | 1.58E-17 |
| *O. sinensis* | 51 | 122790 | P | 51 | 148794 | 7.74E-16 |
| *O. sinensis* | 51 | 26154 | P | 51 | 148777 | 2.11E-19 |
| *O. sinensis* | 51 | 147613 | P | 51 | 148777 | 2.11E-19 |
| *O. sinensis* | 51 | 14173 | P | 51 | 148777 | 2.11E-19 |
| *O. sinensis* | 50 | 109877 | F | 50 | 157127 | 6.07E-17 |
| *O. sinensis* | 50 | 110046 | F | 50 | 150572 | 5.51E-21 |
| *O. sinensis* | 50 | 110046 | F | 50 | 156071 | 5.51E-21 |
| *O. sinensis* | 50 | 30834 | F | 50 | 30841 | 5.51E-21 |
| *O. sinensis* | 50 | 27542 | F | 50 | 120771 | 6.07E-17 |
| *O. sinensis* | 50 | 55848 | F | 50 | 108641 | 2.91E-15 |
| *O. sinensis* | 50 | 55848 | F | 50 | 135128 | 2.91E-15 |
| *O. sinensis* | 50 | 45278 | F | 50 | 70654 | 2.91E-15 |
| *O. sinensis* | 50 | 135128 | F | 50 | 157215 | 8.26E-19 |
| *O. sinensis* | 50 | 108641 | F | 50 | 157215 | 8.26E-19 |
| *O. sinensis* | 50 | 26143 | F | 50 | 122796 | 2.91E-15 |
| *O. sinensis* | 50 | 17523 | F | 50 | 24448 | 2.91E-15 |
| *O. sinensis* | 50 | 28179 | P | 50 | 143351 | 6.07E-17 |
| *O. sinensis* | 50 | 21225 | P | 50 | 139030 | 8.26E-19 |
| *O. sinensis* | 50 | 109877 | P | 50 | 120866 | 8.26E-19 |
| *O. sinensis* | 50 | 30834 | P | 50 | 98108 | 5.51E-21 |
| *O. sinensis* | 50 | 30841 | C | 50 | 98108 | 5.51E-21 |
| *O. sinensis* | 49 | 24510 | F | 49 | 150689 | 2.20E-20 |
| *O. sinensis* | 49 | 6799 | F | 49 | 34470 | 1.10E-14 |
| *O. sinensis* | 49 | 63415 | F | 49 | 63664 | 3.24E-18 |
| *O. sinensis* | 49 | 63415 | F | 49 | 63541 | 3.24E-18 |
| *O. sinensis* | 49 | 109992 | F | 49 | 126884 | 3.24E-18 |
| *O. sinensis* | 49 | 98108 | F | 49 | 98136 | 2.20E-20 |
| *O. sinensis* | 49 | 55845 | F | 49 | 157212 | 1.10E-14 |
| *O. sinensis* | 49 | 111791 | P | 49 | 150140 | 3.24E-18 |
| *O. sinensis* | 49 | 16479 | P | 49 | 140335 | 2.20E-20 |
| *O. sinensis* | 49 | 27553 | P | 49 | 27553 | 3.24E-18 |
| *O. sinensis* | 49 | 30834 | C | 49 | 98136 | 2.20E-20 |
| *O. sinensis* | 48 | 113426 | F | 48 | 143406 | 8.81E-20 |
| *O. sinensis* | 48 | 34509 | F | 48 | 60604 | 4.11E-14 |
| *O. sinensis* | 48 | 113400 | F | 48 | 150075 | 4.11E-14 |
| *O. sinensis* | 48 | 55505 | F | 48 | 113400 | 4.11E-14 |
| *O. sinensis* | 48 | 27033 | F | 48 | 120798 | 4.11E-14 |
| *O. sinensis* | 48 | 17466 | F | 48 | 120798 | 4.11E-14 |
| *O. sinensis* | 48 | 120937 | F | 48 | 150075 | 8.94E-16 |
| *O. sinensis* | 48 | 55714 | F | 48 | 150075 | 8.94E-16 |
| *O. sinensis* | 48 | 55505 | F | 48 | 120937 | 8.94E-16 |
| *O. sinensis* | 48 | 55505 | F | 48 | 55714 | 8.94E-16 |
| *O. sinensis* | 48 | 98178 | P | 48 | 98466 | 1.27E-17 |
| *O. sinensis* | 48 | 27033 | P | 48 | 55847 | 1.27E-17 |
| *O. sinensis* | 48 | 17466 | P | 48 | 55847 | 1.27E-17 |
| *O. sinensis* | 47 | 56987 | F | 47 | 86162 | 1.54E-13 |
| *O. sinensis* | 47 | 63205 | F | 47 | 63241 | 1.54E-13 |
| *O. sinensis* | 47 | 28164 | F | 47 | 135131 | 4.97E-17 |
| *O. sinensis* | 47 | 28164 | F | 47 | 108644 | 4.97E-17 |
| *O. sinensis* | 47 | 70318 | P | 47 | 135184 | 1.54E-13 |
| *O. sinensis* | 47 | 108644 | P | 47 | 120795 | 1.54E-13 |
| *O. sinensis* | 47 | 27545 | P | 47 | 113513 | 4.97E-17 |
| *O. sinensis* | 47 | 55505 | P | 47 | 109811 | 3.43E-15 |
| *O. sinensis* | 47 | 29455 | P | 47 | 109707 | 3.43E-15 |
| *O. sinensis* | 47 | 70350 | P | 47 | 108665 | 1.54E-13 |
| *O. sinensis* | 47 | 27034 | P | 47 | 28160 | 1.54E-13 |
| *O. sinensis* | 47 | 17467 | P | 47 | 28160 | 1.54E-13 |
| *O. sinensis* | 46 | 57997 | F | 46 | 58033 | 1.95E-16 |
| *O. sinensis* | 46 | 83285 | F | 46 | 83312 | 5.78E-13 |
| *O. sinensis* | 46 | 120793 | F | 46 | 143367 | 5.78E-13 |
| *O. sinensis* | 46 | 27626 | F | 46 | 109895 | 1.41E-18 |
| *O. sinensis* | 46 | 120793 | P | 46 | 157221 | 5.78E-13 |
| *O. sinensis* | 46 | 143405 | P | 46 | 157029 | 1.41E-18 |
| *O. sinensis* | 46 | 55854 | P | 46 | 143367 | 5.78E-13 |
| *O. sinensis* | 46 | 17467 | P | 46 | 135128 | 5.78E-13 |
| *O. sinensis* | 46 | 27034 | P | 46 | 135128 | 5.78E-13 |
| *O. sinensis* | 46 | 128923 | P | 46 | 135104 | 1.41E-18 |
| *O. sinensis* | 46 | 108617 | P | 46 | 128923 | 1.41E-18 |
| *O. sinensis* | 46 | 28167 | P | 46 | 120793 | 5.78E-13 |
| *O. sinensis* | 46 | 28717 | P | 46 | 113495 | 5.78E-13 |
| *O. sinensis* | 46 | 28716 | P | 46 | 108648 | 5.78E-13 |
| *O. sinensis* | 46 | 27034 | P | 46 | 108641 | 5.78E-13 |
| *O. sinensis* | 46 | 17467 | P | 46 | 108641 | 5.78E-13 |
| *O. sinensis* | 46 | 25791 | P | 46 | 25791 | 1.31E-14 |
| *O. sinensis* | 45 | 28717 | F | 45 | 120793 | 5.02E-14 |
| *O. sinensis* | 45 | 121902 | F | 45 | 146513 | 5.02E-14 |
| *O. sinensis* | 45 | 7047 | F | 45 | 34691 | 2.16E-12 |
| *O. sinensis* | 45 | 121545 | F | 45 | 121945 | 5.64E-18 |
| *O. sinensis* | 45 | 121945 | F | 45 | 146557 | 5.64E-18 |
| *O. sinensis* | 45 | 87564 | P | 45 | 111768 | 2.16E-12 |
| *O. sinensis* | 45 | 26074 | P | 45 | 108132 | 5.64E-18 |
| *O. sinensis* | 45 | 28717 | P | 45 | 55855 | 5.02E-14 |
| *O. sinensis* | 45 | 18823 | P | 45 | 18823 | 2.16E-12 |
| *O. sinensis* | 44 | 28755 | F | 44 | 70394 | 2.98E-15 |
| *O. sinensis* | 44 | 109768 | F | 44 | 120831 | 8.07E-12 |
| *O. sinensis* | 44 | 4044 | F | 44 | 51925 | 8.07E-12 |
| *O. sinensis* | 44 | 14562 | F | 44 | 18067 | 8.07E-12 |
| *O. sinensis* | 44 | 93845 | F | 44 | 142330 | 8.07E-12 |
| *O. sinensis* | 44 | 28188 | F | 44 | 29461 | 8.07E-12 |
| *O. sinensis* | 44 | 13719 | F | 44 | 111752 | 8.07E-12 |
| *O. sinensis* | 44 | 55734 | P | 44 | 157036 | 2.98E-15 |
| *O. sinensis* | 44 | 135134 | P | 44 | 143369 | 8.07E-12 |
| *O. sinensis* | 44 | 108647 | P | 44 | 143369 | 2.98E-15 |
| *O. sinensis* | 44 | 56203 | P | 44 | 109728 | 2.98E-15 |
| *O. sinensis* | 43 | 150689 | F | 43 | 156190 | 9.02E-17 |
| *O. sinensis* | 43 | 135175 | F | 43 | 135358 | 9.02E-17 |
| *O. sinensis* | 43 | 6809 | F | 43 | 34480 | 3.01E-11 |
| *O. sinensis* | 43 | 58833 | F | 43 | 94507 | 3.01E-11 |
| *O. sinensis* | 43 | 18823 | F | 43 | 120784 | 3.01E-11 |
| *O. sinensis* | 43 | 30855 | F | 43 | 55763 | 1.16E-14 |
| *O. sinensis* | 43 | 30834 | F | 43 | 30848 | 9.02E-17 |
| *O. sinensis* | 43 | 27033 | F | 43 | 28722 | 3.01E-11 |
| *O. sinensis* | 43 | 17466 | F | 43 | 28722 | 3.01E-11 |
| *O. sinensis* | 43 | 16464 | F | 43 | 128847 | 7.33E-13 |
| *O. sinensis* | 43 | 17551 | F | 43 | 150654 | 1.16E-14 |
| *O. sinensis* | 43 | 108648 | F | 43 | 113496 | 3.01E-11 |
| *O. sinensis* | 43 | 113496 | F | 43 | 135135 | 3.01E-11 |
| *O. sinensis* | 43 | 24401 | F | 43 | 110053 | 9.02E-17 |
| *O. sinensis* | 43 | 27034 | P | 43 | 157218 | 3.01E-11 |
| *O. sinensis* | 43 | 17467 | P | 43 | 157218 | 3.01E-11 |
| *O. sinensis* | 43 | 109728 | P | 43 | 156392 | 3.01E-11 |
| *O. sinensis* | 43 | 109728 | P | 43 | 135294 | 3.01E-11 |
| *O. sinensis* | 43 | 17402 | P | 43 | 135175 | 9.02E-17 |
| *O. sinensis* | 43 | 87566 | P | 43 | 128859 | 3.01E-11 |
| *O. sinensis* | 43 | 108668 | P | 43 | 128859 | 3.01E-11 |
| *O. sinensis* | 43 | 108668 | P | 43 | 111768 | 3.01E-11 |
| *O. sinensis* | 43 | 56973 | P | 43 | 94166 | 1.16E-14 |
| *O. sinensis* | 43 | 30848 | C | 43 | 98108 | 9.02E-17 |
| *O. sinensis* | 42 | 98108 | F | 42 | 98143 | 3.61E-16 |
| *O. sinensis* | 42 | 17403 | F | 42 | 70332 | 2.80E-12 |
| *O. sinensis* | 42 | 17551 | F | 42 | 24476 | 2.80E-12 |
| *O. sinensis* | 42 | 17551 | F | 42 | 156156 | 4.55E-14 |
| *O. sinensis* | 42 | 28171 | F | 42 | 113499 | 1.12E-10 |
| *O. sinensis* | 42 | 113499 | F | 42 | 157225 | 1.12E-10 |
| *O. sinensis* | 42 | 120815 | P | 42 | 157203 | 1.12E-10 |
| *O. sinensis* | 42 | 29972 | P | 42 | 155309 | 1.12E-10 |
| *O. sinensis* | 42 | 126616 | P | 42 | 151469 | 3.61E-16 |
| *O. sinensis* | 42 | 113499 | P | 42 | 143367 | 1.12E-10 |
| *O. sinensis* | 42 | 70332 | P | 42 | 135358 | 2.80E-12 |
| *O. sinensis* | 42 | 70400 | P | 42 | 135100 | 3.61E-16 |
| *O. sinensis* | 42 | 23028 | P | 42 | 122789 | 4.55E-14 |
| *O. sinensis* | 42 | 108076 | P | 42 | 122789 | 4.55E-14 |
| *O. sinensis* | 42 | 56381 | P | 42 | 122789 | 2.80E-12 |
| *O. sinensis* | 42 | 23018 | P | 42 | 26146 | 3.61E-16 |
| *O. sinensis* | 42 | 30834 | C | 42 | 98143 | 3.61E-16 |
| *O. sinensis* | 41 | 4102 | F | 41 | 51990 | 4.15E-10 |
| *O. sinensis* | 41 | 23485 | F | 41 | 98102 | 1.07E-11 |
| *O. sinensis* | 41 | 56975 | F | 41 | 109728 | 4.15E-10 |
| *O. sinensis* | 41 | 24444 | F | 41 | 121889 | 4.15E-10 |
| *O. sinensis* | 41 | 121889 | F | 41 | 150622 | 4.15E-10 |
| *O. sinensis* | 41 | 9455 | F | 41 | 127156 | 4.15E-10 |
| *O. sinensis* | 41 | 48326 | F | 41 | 115056 | 4.15E-10 |
| *O. sinensis* | 41 | 27034 | F | 41 | 143373 | 1.07E-11 |
| *O. sinensis* | 41 | 17467 | F | 41 | 143373 | 1.07E-11 |
| *O. sinensis* | 41 | 56250 | F | 41 | 94210 | 4.15E-10 |
| *O. sinensis* | 41 | 94210 | F | 41 | 156438 | 4.15E-10 |
| *O. sinensis* | 41 | 28681 | F | 41 | 87497 | 1.44E-15 |
| *O. sinensis* | 41 | 7038 | F | 41 | 60753 | 4.15E-10 |
| *O. sinensis* | 41 | 7030 | F | 41 | 60745 | 1.07E-11 |
| *O. sinensis* | 41 | 58848 | F | 41 | 94522 | 4.15E-10 |
| *O. sinensis* | 41 | 29461 | F | 41 | 157242 | 1.07E-11 |
| *O. sinensis* | 41 | 24403 | F | 41 | 110203 | 1.44E-15 |
| *O. sinensis* | 41 | 110203 | F | 41 | 150581 | 1.44E-15 |
| *O. sinensis* | 41 | 110203 | F | 41 | 156080 | 1.44E-15 |
| *O. sinensis* | 41 | 16533 | P | 41 | 141147 | 4.15E-10 |
| *O. sinensis* | 41 | 16533 | P | 41 | 140296 | 4.15E-10 |
| *O. sinensis* | 41 | 84211 | P | 41 | 84216 | 4.15E-10 |
| *O. sinensis* | 41 | 155418 | R | 41 | 155422 | 4.15E-10 |
| *O. sinensis* | 40 | 28761 | F | 40 | 128931 | 5.77E-15 |
| *O. sinensis* | 40 | 44278 | F | 40 | 129104 | 5.77E-15 |
| *O. sinensis* | 40 | 87512 | F | 40 | 128827 | 1.54E-09 |
| *O. sinensis* | 40 | 70354 | F | 40 | 111771 | 1.54E-09 |
| *O. sinensis* | 40 | 70354 | F | 40 | 128862 | 1.54E-09 |
| *O. sinensis* | 40 | 7417 | F | 40 | 128214 | 1.54E-09 |
| *O. sinensis* | 40 | 28702 | F | 40 | 109708 | 6.93E-13 |
| *O. sinensis* | 40 | 23491 | F | 40 | 98150 | 4.05E-11 |
| *O. sinensis* | 40 | 108672 | F | 40 | 135159 | 4.05E-11 |
| *O. sinensis* | 40 | 18826 | F | 40 | 28711 | 1.54E-09 |
| *O. sinensis* | 40 | 121893 | F | 40 | 151473 | 4.05E-11 |
| *O. sinensis* | 40 | 120878 | P | 40 | 157125 | 5.77E-15 |
| *O. sinensis* | 40 | 27013 | P | 40 | 156415 | 5.77E-15 |
| *O. sinensis* | 40 | 17446 | P | 40 | 156415 | 5.77E-15 |
| *O. sinensis* | 40 | 111800 | P | 40 | 150140 | 5.77E-15 |
| *O. sinensis* | 40 | 120799 | P | 40 | 135134 | 1.54E-09 |
| *O. sinensis* | 40 | 87578 | P | 40 | 128850 | 1.54E-09 |
| *O. sinensis* | 40 | 18826 | P | 40 | 113507 | 1.54E-09 |
| *O. sinensis* | 40 | 23776 | P | 40 | 92708 | 1.54E-09 |
| *O. sinensis* | 40 | 14376 | P | 40 | 92708 | 1.54E-09 |
| *O. sinensis* | 40 | 27013 | P | 40 | 56227 | 5.77E-15 |
| *O. sinensis* | 40 | 17446 | P | 40 | 56227 | 5.77E-15 |
| *O. sinensis* | 40 | 18826 | P | 40 | 55866 | 1.54E-09 |
| *O. sinensis* | 39 | 13780 | F | 39 | 121988 | 5.70E-09 |
| *O. sinensis* | 39 | 16468 | F | 39 | 111760 | 1.54E-10 |
| *O. sinensis* | 39 | 8461 | F | 39 | 8546 | 5.70E-09 |
| *O. sinensis* | 39 | 27042 | F | 39 | 120807 | 1.54E-10 |
| *O. sinensis* | 39 | 17475 | F | 39 | 120807 | 1.54E-10 |
| *O. sinensis* | 39 | 12913 | F | 39 | 138780 | 2.70E-12 |
| *O. sinensis* | 39 | 72688 | F | 39 | 94524 | 5.70E-09 |
| *O. sinensis* | 39 | 17398 | F | 39 | 86089 | 5.70E-09 |
| *O. sinensis* | 39 | 7500 | F | 39 | 9877 | 5.70E-09 |
| *O. sinensis* | 39 | 28723 | F | 39 | 143373 | 1.54E-10 |
| *O. sinensis* | 39 | 24369 | F | 39 | 126914 | 1.54E-10 |
| *O. sinensis* | 39 | 27563 | F | 39 | 55872 | 2.70E-12 |
| *O. sinensis* | 39 | 21165 | F | 39 | 142502 | 5.70E-09 |
| *O. sinensis* | 39 | 28723 | P | 39 | 157222 | 1.54E-10 |
| *O. sinensis* | 39 | 17475 | P | 39 | 157214 | 5.70E-09 |
| *O. sinensis* | 39 | 27042 | P | 39 | 157214 | 5.70E-09 |
| *O. sinensis* | 39 | 29989 | P | 39 | 155295 | 5.70E-09 |
| *O. sinensis* | 39 | 122807 | P | 39 | 148789 | 5.70E-09 |
| *O. sinensis* | 39 | 28723 | P | 39 | 135135 | 5.70E-09 |
| *O. sinensis* | 39 | 87578 | P | 39 | 111760 | 5.70E-09 |
| *O. sinensis* | 39 | 70358 | P | 39 | 108665 | 1.54E-10 |
| *O. sinensis* | 39 | 14104 | P | 39 | 108099 | 2.31E-14 |
| *O. sinensis* | 39 | 17475 | P | 39 | 28160 | 1.54E-10 |
| *O. sinensis* | 39 | 27042 | P | 39 | 28160 | 1.54E-10 |
| *O. sinensis* | 38 | 110097 | F | 38 | 110248 | 2.10E-08 |
| *O. sinensis* | 38 | 70400 | F | 38 | 128931 | 9.24E-14 |
| *O. sinensis* | 38 | 55740 | F | 38 | 143406 | 9.24E-14 |
| *O. sinensis* | 38 | 88223 | F | 38 | 128217 | 2.10E-08 |
| *O. sinensis* | 38 | 44330 | F | 38 | 129162 | 2.10E-08 |
| *O. sinensis* | 38 | 30832 | F | 38 | 55761 | 9.24E-14 |
| *O. sinensis* | 38 | 121582 | F | 38 | 126619 | 2.10E-08 |
| *O. sinensis* | 38 | 9369 | F | 38 | 144649 | 2.10E-08 |
| *O. sinensis* | 38 | 34448 | F | 38 | 41985 | 2.10E-08 |
| *O. sinensis* | 38 | 18822 | F | 38 | 27554 | 2.10E-08 |
| *O. sinensis* | 38 | 6782 | F | 38 | 60548 | 2.10E-08 |
| *O. sinensis* | 38 | 34127 | F | 38 | 66897 | 9.24E-14 |
| *O. sinensis* | 38 | 110022 | F | 38 | 126914 | 2.10E-08 |
| *O. sinensis* | 38 | 126914 | F | 38 | 156047 | 5.85E-10 |
| *O. sinensis* | 38 | 30377 | F | 38 | 38987 | 2.10E-08 |
| *O. sinensis* | 38 | 94210 | F | 38 | 135340 | 1.05E-11 |
| *O. sinensis* | 38 | 94210 | F | 38 | 150192 | 1.05E-11 |
| *O. sinensis* | 38 | 63178 | F | 38 | 63250 | 2.10E-08 |
| *O. sinensis* | 38 | 17522 | F | 38 | 146503 | 2.10E-08 |
| *O. sinensis* | 38 | 16528 | P | 38 | 156388 | 9.24E-14 |
| *O. sinensis* | 38 | 30015 | P | 38 | 155270 | 2.10E-08 |
| *O. sinensis* | 38 | 121582 | P | 38 | 151470 | 2.10E-08 |
| *O. sinensis* | 38 | 16528 | P | 38 | 135290 | 9.24E-14 |
| *O. sinensis* | 38 | 28761 | P | 38 | 135104 | 9.24E-14 |
| *O. sinensis* | 38 | 28761 | P | 38 | 108617 | 9.24E-14 |
| *O. sinensis* | 38 | 70400 | P | 38 | 108617 | 9.24E-14 |
| *O. sinensis* | 38 | 18822 | P | 38 | 27563 | 5.85E-10 |
| *O. sinensis* | 37 | 17561 | F | 37 | 94228 | 7.75E-08 |
| *O. sinensis* | 37 | 41107 | F | 37 | 128215 | 7.75E-08 |
| *O. sinensis* | 37 | 121493 | F | 37 | 128850 | 2.21E-09 |
| *O. sinensis* | 37 | 72677 | F | 37 | 94513 | 7.75E-08 |
| *O. sinensis* | 37 | 45472 | F | 37 | 45502 | 7.75E-08 |
| *O. sinensis* | 37 | 70062 | F | 37 | 129597 | 7.75E-08 |
| *O. sinensis* | 37 | 44799 | F | 37 | 129597 | 7.75E-08 |
| *O. sinensis* | 37 | 14768 | F | 37 | 98402 | 7.75E-08 |
| *O. sinensis* | 37 | 9350 | F | 37 | 129755 | 2.21E-09 |
| *O. sinensis* | 37 | 9350 | F | 37 | 144630 | 2.21E-09 |
| *O. sinensis* | 37 | 18832 | F | 37 | 143367 | 7.75E-08 |
| *O. sinensis* | 37 | 41118 | F | 37 | 88232 | 7.75E-08 |
| *O. sinensis* | 37 | 30475 | F | 37 | 34617 | 4.10E-11 |
| *O. sinensis* | 37 | 27563 | F | 37 | 113513 | 4.10E-11 |
| *O. sinensis* | 37 | 27555 | F | 37 | 120784 | 4.10E-11 |
| *O. sinensis* | 37 | 17523 | F | 37 | 121893 | 2.21E-09 |
| *O. sinensis* | 37 | 121893 | F | 37 | 156128 | 7.75E-08 |
| *O. sinensis* | 37 | 28192 | P | 37 | 143351 | 4.10E-11 |
| *O. sinensis* | 37 | 29465 | P | 37 | 143351 | 7.75E-08 |
| *O. sinensis* | 37 | 16083 | P | 37 | 140500 | 7.75E-08 |
| *O. sinensis* | 37 | 70366 | P | 37 | 135146 | 7.75E-08 |
| *O. sinensis* | 37 | 21242 | P | 37 | 126945 | 7.75E-08 |
| *O. sinensis* | 37 | 17560 | P | 37 | 121489 | 7.75E-08 |
| *O. sinensis* | 37 | 27563 | P | 37 | 120784 | 3.70E-13 |
| *O. sinensis* | 37 | 27555 | P | 37 | 113513 | 3.70E-13 |
| *O. sinensis* | 37 | 27033 | P | 37 | 113499 | 3.70E-13 |
| *O. sinensis* | 37 | 17466 | P | 37 | 113499 | 3.70E-13 |
| *O. sinensis* | 37 | 29465 | P | 37 | 109707 | 4.10E-11 |
| *O. sinensis* | 37 | 70366 | P | 37 | 108659 | 7.75E-08 |
| *O. sinensis* | 37 | 26114 | P | 37 | 108101 | 3.70E-13 |
| *O. sinensis* | 37 | 55763 | P | 37 | 98107 | 3.70E-13 |
| *O. sinensis* | 37 | 23739 | P | 37 | 92749 | 2.21E-09 |
| *O. sinensis* | 37 | 18820 | P | 37 | 70371 | 7.75E-08 |
| *O. sinensis* | 37 | 25298 | P | 37 | 70371 | 7.75E-08 |
| *O. sinensis* | 37 | 40663 | P | 37 | 40663 | 7.75E-08 |
| *O. sinensis* | 37 | 155418 | R | 37 | 155421 | 7.75E-08 |
| *O. sinensis* | 36 | 111760 | F | 36 | 121494 | 8.38E-09 |
| *O. sinensis* | 36 | 23731 | F | 36 | 111752 | 2.85E-07 |
| *O. sinensis* | 36 | 30848 | F | 36 | 55763 | 1.48E-12 |
| *O. sinensis* | 36 | 30841 | F | 36 | 55763 | 1.48E-12 |
| *O. sinensis* | 36 | 30834 | F | 36 | 30855 | 1.48E-12 |
| *O. sinensis* | 36 | 110196 | F | 36 | 139023 | 1.48E-12 |
| *O. sinensis* | 36 | 6979 | F | 36 | 39094 | 2.85E-07 |
| *O. sinensis* | 36 | 8417 | F | 36 | 8551 | 2.85E-07 |
| *O. sinensis* | 36 | 109712 | F | 36 | 143356 | 8.38E-09 |
| *O. sinensis* | 36 | 57342 | F | 36 | 72694 | 1.60E-10 |
| *O. sinensis* | 36 | 121510 | F | 36 | 121911 | 1.60E-10 |
| *O. sinensis* | 36 | 30423 | F | 36 | 39033 | 8.38E-09 |
| *O. sinensis* | 36 | 28719 | F | 36 | 87566 | 2.85E-07 |
| *O. sinensis* | 36 | 55682 | F | 36 | 121487 | 8.38E-09 |
| *O. sinensis* | 36 | 13727 | F | 36 | 128851 | 2.85E-07 |
| *O. sinensis* | 36 | 151470 | F | 36 | 156125 | 2.85E-07 |
| *O. sinensis* | 36 | 109712 | P | 36 | 157242 | 8.38E-09 |
| *O. sinensis* | 36 | 84235 | P | 36 | 156127 | 2.85E-07 |
| *O. sinensis* | 36 | 126621 | P | 36 | 156125 | 2.85E-07 |
| *O. sinensis* | 36 | 87556 | P | 36 | 109722 | 1.60E-10 |
| *O. sinensis* | 36 | 28188 | P | 36 | 109712 | 8.38E-09 |
| *O. sinensis* | 36 | 87570 | P | 36 | 108651 | 2.85E-07 |
| *O. sinensis* | 36 | 55763 | P | 36 | 98143 | 1.48E-12 |
| *O. sinensis* | 36 | 55763 | P | 36 | 98136 | 1.48E-12 |
| *O. sinensis* | 36 | 55763 | P | 36 | 98129 | 1.48E-12 |
| *O. sinensis* | 36 | 55763 | P | 36 | 98122 | 1.48E-12 |
| *O. sinensis* | 36 | 55763 | P | 36 | 98115 | 1.48E-12 |
| *O. sinensis* | 36 | 72885 | P | 36 | 92803 | 8.38E-09 |
| *O. sinensis* | 36 | 13727 | P | 36 | 87581 | 2.85E-07 |
| *O. sinensis* | 36 | 70358 | P | 36 | 87566 | 2.85E-07 |
| *O. sinensis* | 36 | 23490 | P | 36 | 55764 | 8.38E-09 |
| *O. sinensis* | 36 | 23490 | P | 36 | 30856 | 8.38E-09 |
| *O. sinensis* | 36 | 28706 | P | 36 | 29461 | 1.60E-10 |
| *O. sinensis* | 36 | 28192 | P | 36 | 28702 | 8.38E-09 |
| *O. sinensis* | 36 | 55763 | R | 36 | 55763 | 1.48E-12 |
| *O. sinensis* | 36 | 55763 | R | 36 | 30834 | 1.48E-12 |
| *O. sinensis* | 36 | 30855 | R | 36 | 55763 | 1.48E-12 |
| *O. sinensis* | 36 | 30848 | R | 36 | 55763 | 1.48E-12 |
| *O. sinensis* | 36 | 30841 | R | 36 | 55763 | 1.48E-12 |
| *O. sinensis* | 36 | 55763 | C | 36 | 98108 | 1.48E-12 |
| *O. sinensis* | 36 | 55763 | C | 36 | 98115 | 1.48E-12 |
| *O. sinensis* | 36 | 55763 | C | 36 | 98122 | 1.48E-12 |
| *O. sinensis* | 36 | 55763 | C | 36 | 98129 | 1.48E-12 |
| *O. sinensis* | 36 | 55763 | C | 36 | 98136 | 1.48E-12 |
| *O. sinensis* | 36 | 55763 | C | 36 | 98143 | 1.48E-12 |
| *O. sinensis* | 36 | 30855 | C | 36 | 98108 | 1.48E-12 |
| *O. sinensis* | 35 | 56279 | F | 35 | 156465 | 3.17E-08 |
| *O. sinensis* | 35 | 135376 | F | 35 | 150227 | 3.17E-08 |
| *O. sinensis* | 35 | 55902 | F | 35 | 108705 | 1.04E-06 |
| *O. sinensis* | 35 | 55810 | F | 35 | 157178 | 1.04E-06 |
| *O. sinensis* | 35 | 109777 | F | 35 | 120840 | 3.17E-08 |
| *O. sinensis* | 35 | 16527 | F | 35 | 109736 | 3.17E-08 |
| *O. sinensis* | 35 | 6838 | F | 35 | 38989 | 1.04E-06 |
| *O. sinensis* | 35 | 25300 | F | 35 | 27554 | 1.04E-06 |
| *O. sinensis* | 35 | 135165 | F | 35 | 141205 | 3.17E-08 |
| *O. sinensis* | 35 | 126126 | F | 35 | 135165 | 3.17E-08 |
| *O. sinensis* | 35 | 135165 | F | 35 | 140354 | 3.17E-08 |
| *O. sinensis* | 35 | 70739 | F | 35 | 148222 | 1.04E-06 |
| *O. sinensis* | 35 | 98108 | F | 35 | 98150 | 5.91E-12 |
| *O. sinensis* | 35 | 23491 | F | 35 | 98143 | 3.17E-08 |
| *O. sinensis* | 35 | 23491 | F | 35 | 98136 | 3.17E-08 |
| *O. sinensis* | 35 | 23491 | F | 35 | 98129 | 3.17E-08 |
| *O. sinensis* | 35 | 23491 | F | 35 | 98122 | 3.17E-08 |
| *O. sinensis* | 35 | 23491 | F | 35 | 98115 | 3.17E-08 |
| *O. sinensis* | 35 | 29457 | F | 35 | 70367 | 3.17E-08 |
| *O. sinensis* | 35 | 24372 | F | 35 | 156050 | 6.21E-10 |
| *O. sinensis* | 35 | 58857 | F | 35 | 72695 | 3.17E-08 |
| *O. sinensis* | 35 | 57343 | F | 35 | 58857 | 1.04E-06 |
| *O. sinensis* | 35 | 110117 | F | 35 | 150710 | 5.91E-12 |
| *O. sinensis* | 35 | 70371 | F | 35 | 113516 | 1.04E-06 |
| *O. sinensis* | 35 | 40408 | F | 35 | 114922 | 1.04E-06 |
| *O. sinensis* | 35 | 24372 | F | 35 | 110025 | 1.04E-06 |
| *O. sinensis* | 35 | 9348 | F | 35 | 40553 | 3.17E-08 |
| *O. sinensis* | 35 | 18825 | F | 35 | 113507 | 6.21E-10 |
| *O. sinensis* | 35 | 18825 | F | 35 | 55866 | 6.21E-10 |
| *O. sinensis* | 35 | 14332 | F | 35 | 56916 | 5.91E-12 |
| *O. sinensis* | 35 | 17392 | F | 35 | 56902 | 1.04E-06 |
| *O. sinensis* | 35 | 155414 | F | 35 | 155424 | 1.04E-06 |
| *O. sinensis* | 35 | 28021 | F | 35 | 109688 | 6.21E-10 |
| *O. sinensis* | 35 | 146502 | F | 35 | 151471 | 3.17E-08 |
| *O. sinensis* | 35 | 126011 | F | 35 | 141098 | 5.91E-12 |
| *O. sinensis* | 35 | 150644 | P | 35 | 156146 | 1.04E-06 |
| *O. sinensis* | 35 | 156146 | P | 35 | 156146 | 1.04E-06 |
| *O. sinensis* | 35 | 150644 | P | 35 | 150644 | 1.04E-06 |
| *O. sinensis* | 35 | 84235 | P | 35 | 150626 | 3.17E-08 |
| *O. sinensis* | 35 | 14332 | P | 35 | 150213 | 5.91E-12 |
| *O. sinensis* | 35 | 140419 | P | 35 | 147977 | 3.17E-08 |
| *O. sinensis* | 35 | 121584 | P | 35 | 146502 | 1.04E-06 |
| *O. sinensis* | 35 | 126621 | P | 35 | 146502 | 3.17E-08 |
| *O. sinensis* | 35 | 135146 | P | 35 | 143366 | 1.04E-06 |
| *O. sinensis* | 35 | 70349 | P | 35 | 141205 | 1.04E-06 |
| *O. sinensis* | 35 | 70349 | P | 35 | 140354 | 1.04E-06 |
| *O. sinensis* | 35 | 86093 | P | 35 | 135183 | 1.04E-06 |
| *O. sinensis* | 35 | 120807 | P | 35 | 135131 | 1.04E-06 |
| *O. sinensis* | 35 | 70349 | P | 35 | 126126 | 1.04E-06 |
| *O. sinensis* | 35 | 84235 | P | 35 | 121893 | 1.04E-06 |
| *O. sinensis* | 35 | 28178 | P | 35 | 120793 | 3.17E-08 |
| *O. sinensis* | 35 | 18825 | P | 35 | 120792 | 3.17E-08 |
| *O. sinensis* | 35 | 70371 | P | 35 | 120783 | 3.17E-08 |
| *O. sinensis* | 35 | 55887 | P | 35 | 120771 | 6.21E-10 |
| *O. sinensis* | 35 | 109767 | P | 35 | 113468 | 1.04E-06 |
| *O. sinensis* | 35 | 94172 | P | 35 | 109728 | 1.04E-06 |
| *O. sinensis* | 35 | 70367 | P | 35 | 109717 | 1.04E-06 |
| *O. sinensis* | 35 | 18834 | P | 35 | 108656 | 1.04E-06 |
| *O. sinensis* | 35 | 55764 | P | 35 | 98150 | 5.91E-12 |
| *O. sinensis* | 35 | 24448 | P | 35 | 84235 | 3.17E-08 |
| *O. sinensis* | 35 | 25300 | P | 35 | 55875 | 1.04E-06 |
| *O. sinensis* | 35 | 23491 | P | 35 | 30849 | 3.17E-08 |
| *O. sinensis* | 35 | 23491 | P | 35 | 30842 | 3.17E-08 |
| *O. sinensis* | 35 | 23491 | P | 35 | 30835 | 3.17E-08 |
| *O. sinensis* | 35 | 25300 | P | 35 | 27566 | 3.17E-08 |
| *O. sinensis* | 35 | 21246 | P | 35 | 24397 | 3.17E-08 |
| *O. sinensis* | 35 | 17541 | P | 35 | 17541 | 1.04E-06 |
| *O. sinensis* | 35 | 80041 | R | 35 | 80041 | 3.17E-08 |
| *O. sinensis* | 35 | 98144 | R | 35 | 23491 | 3.17E-08 |
| *O. sinensis* | 35 | 98137 | R | 35 | 23491 | 3.17E-08 |
| *O. sinensis* | 35 | 98130 | R | 35 | 23491 | 3.17E-08 |
| *O. sinensis* | 35 | 98123 | R | 35 | 23491 | 3.17E-08 |
| *O. sinensis* | 35 | 98116 | R | 35 | 23491 | 3.17E-08 |
| *O. sinensis* | 35 | 98109 | R | 35 | 23491 | 3.17E-08 |
| *O. sinensis* | 35 | 30859 | R | 35 | 30859 | 3.17E-08 |
| *O. sinensis* | 35 | 155410 | R | 35 | 155421 | 1.04E-06 |
| *O. sinensis* | 35 | 55763 | C | 35 | 98150 | 5.91E-12 |
| *O. sinensis* | 35 | 30834 | C | 35 | 98150 | 5.91E-12 |
| *O. sinensis* | 35 | 55763 | C | 35 | 23491 | 3.17E-08 |
| *O. sinensis* | 35 | 30834 | C | 35 | 23491 | 3.17E-08 |
| *O. sinensis* | 35 | 30855 | C | 35 | 23491 | 3.17E-08 |
| *O. sinensis* | 35 | 30848 | C | 35 | 23491 | 3.17E-08 |
| *O. sinensis* | 35 | 30841 | C | 35 | 23491 | 3.17E-08 |
| *O. sinensis* | 34 | 146810 | F | 34 | 148159 | 2.41E-09 |
| *O. sinensis* | 34 | 25301 | F | 34 | 120784 | 1.19E-07 |
| *O. sinensis* | 34 | 45472 | F | 34 | 45532 | 3.82E-06 |
| *O. sinensis* | 34 | 34558 | F | 34 | 42095 | 3.82E-06 |
| *O. sinensis* | 34 | 4037 | F | 34 | 5633 | 2.41E-09 |
| *O. sinensis* | 34 | 84237 | F | 34 | 126621 | 3.82E-06 |
| *O. sinensis* | 34 | 14086 | F | 34 | 147588 | 3.82E-06 |
| *O. sinensis* | 34 | 14086 | F | 34 | 14148 | 3.82E-06 |
| *O. sinensis* | 34 | 27042 | F | 34 | 28731 | 1.19E-07 |
| *O. sinensis* | 34 | 17475 | F | 34 | 28731 | 1.19E-07 |
| *O. sinensis* | 34 | 6886 | F | 34 | 60653 | 3.82E-06 |
| *O. sinensis* | 34 | 17815 | F | 34 | 19221 | 3.82E-06 |
| *O. sinensis* | 34 | 12918 | F | 34 | 102511 | 2.41E-09 |
| *O. sinensis* | 34 | 102511 | F | 34 | 138785 | 2.41E-09 |
| *O. sinensis* | 34 | 3547 | F | 34 | 102511 | 3.82E-06 |
| *O. sinensis* | 34 | 63169 | F | 34 | 63205 | 3.82E-06 |
| *O. sinensis* | 34 | 86271 | F | 34 | 86278 | 1.19E-07 |
| *O. sinensis* | 34 | 83305 | F | 34 | 83332 | 3.82E-06 |
| *O. sinensis* | 34 | 108983 | F | 34 | 128904 | 2.41E-09 |
| *O. sinensis* | 34 | 31076 | F | 34 | 128341 | 3.82E-06 |
| *O. sinensis* | 34 | 27566 | F | 34 | 70371 | 1.19E-07 |
| *O. sinensis* | 34 | 55875 | F | 34 | 70371 | 3.82E-06 |
| *O. sinensis* | 34 | 41347 | F | 34 | 145420 | 3.82E-06 |
| *O. sinensis* | 34 | 27558 | F | 34 | 28711 | 3.82E-06 |
| *O. sinensis* | 34 | 9379 | F | 34 | 144659 | 3.82E-06 |
| *O. sinensis* | 34 | 63148 | F | 34 | 63184 | 3.82E-06 |
| *O. sinensis* | 34 | 121918 | F | 34 | 146529 | 3.82E-06 |
| *O. sinensis* | 34 | 39139 | F | 34 | 60745 | 3.82E-06 |
| *O. sinensis* | 34 | 113508 | F | 34 | 120787 | 1.19E-07 |
| *O. sinensis* | 34 | 55867 | F | 34 | 120787 | 1.19E-07 |
| *O. sinensis* | 34 | 56903 | F | 34 | 86084 | 3.82E-06 |
| *O. sinensis* | 34 | 155418 | F | 34 | 155423 | 1.19E-07 |
| *O. sinensis* | 34 | 152254 | F | 34 | 152369 | 2.36E-11 |
| *O. sinensis* | 34 | 17522 | F | 34 | 151472 | 1.19E-07 |
| *O. sinensis* | 34 | 18832 | P | 34 | 157233 | 3.82E-06 |
| *O. sinensis* | 34 | 121587 | P | 34 | 157065 | 3.82E-06 |
| *O. sinensis* | 34 | 109737 | P | 34 | 156392 | 1.19E-07 |
| *O. sinensis* | 34 | 21246 | P | 34 | 156075 | 1.19E-07 |
| *O. sinensis* | 34 | 84237 | P | 34 | 151472 | 3.82E-06 |
| *O. sinensis* | 34 | 21246 | P | 34 | 150576 | 1.19E-07 |
| *O. sinensis* | 34 | 23043 | P | 34 | 147557 | 3.82E-06 |
| *O. sinensis* | 34 | 55866 | P | 34 | 143367 | 1.19E-07 |
| *O. sinensis* | 34 | 113507 | P | 34 | 143367 | 1.19E-07 |
| *O. sinensis* | 34 | 27563 | P | 34 | 143361 | 3.82E-06 |
| *O. sinensis* | 34 | 18825 | P | 34 | 143367 | 3.82E-06 |
| *O. sinensis* | 34 | 13761 | P | 34 | 141172 | 2.36E-11 |
| *O. sinensis* | 34 | 13761 | P | 34 | 140321 | 2.36E-11 |
| *O. sinensis* | 34 | 86085 | P | 34 | 135375 | 2.41E-09 |
| *O. sinensis* | 34 | 109737 | P | 34 | 135294 | 1.19E-07 |
| *O. sinensis* | 34 | 94226 | P | 34 | 128850 | 1.19E-07 |
| *O. sinensis* | 34 | 17522 | P | 34 | 126621 | 1.19E-07 |
| *O. sinensis* | 34 | 13761 | P | 34 | 126093 | 2.36E-11 |
| *O. sinensis* | 34 | 113468 | P | 34 | 120831 | 3.82E-06 |
| *O. sinensis* | 34 | 120787 | P | 34 | 120792 | 3.82E-06 |
| *O. sinensis* | 34 | 25301 | P | 34 | 113516 | 3.82E-06 |
| *O. sinensis* | 34 | 55867 | P | 34 | 113513 | 3.82E-06 |
| *O. sinensis* | 34 | 113508 | P | 34 | 113513 | 3.82E-06 |
| *O. sinensis* | 34 | 21246 | P | 34 | 110050 | 1.19E-07 |
| *O. sinensis* | 34 | 13790 | P | 34 | 108639 | 3.82E-06 |
| *O. sinensis* | 34 | 14086 | P | 34 | 108091 | 3.82E-06 |
| *O. sinensis* | 34 | 86089 | P | 34 | 94250 | 3.82E-06 |
| *O. sinensis* | 34 | 16467 | P | 34 | 94226 | 1.19E-07 |
| *O. sinensis* | 34 | 14319 | P | 34 | 70411 | 3.82E-06 |
| *O. sinensis* | 34 | 27555 | P | 34 | 70371 | 3.82E-06 |
| *O. sinensis* | 34 | 16528 | P | 34 | 56204 | 1.19E-07 |
| *O. sinensis* | 34 | 55867 | P | 34 | 55872 | 3.82E-06 |
| *O. sinensis* | 34 | 27563 | P | 34 | 28711 | 1.19E-07 |
| *O. sinensis* | 34 | 18825 | P | 34 | 28717 | 1.19E-07 |
| *O. sinensis* | 34 | 18832 | P | 34 | 28179 | 3.82E-06 |
| *O. sinensis* | 34 | 23043 | P | 34 | 26096 | 3.82E-06 |
| *O. sinensis* | 34 | 18834 | P | 34 | 25301 | 1.19E-07 |
| *O. sinensis* | 34 | 23492 | R | 34 | 98151 | 1.19E-07 |
| *O. sinensis* | 34 | 121536 | R | 34 | 121536 | 2.36E-11 |
| *O. sinensis* | 34 | 146548 | R | 34 | 121536 | 2.36E-11 |
| *O. sinensis* | 34 | 146548 | R | 34 | 146548 | 2.36E-11 |
| *O. sinensis* | 33 | 63617 | F | 33 | 63680 | 4.50E-07 |
| *O. sinensis* | 33 | 63557 | F | 33 | 63617 | 4.50E-07 |
| *O. sinensis* | 33 | 94228 | F | 33 | 150664 | 1.39E-05 |
| *O. sinensis* | 33 | 112480 | F | 33 | 128227 | 4.50E-07 |
| *O. sinensis* | 33 | 5746 | F | 33 | 9462 | 1.39E-05 |
| *O. sinensis* | 33 | 81799 | F | 33 | 82864 | 1.39E-05 |
| *O. sinensis* | 33 | 81784 | F | 33 | 82849 | 1.39E-05 |
| *O. sinensis* | 33 | 80027 | F | 33 | 80043 | 1.39E-05 |
| *O. sinensis* | 33 | 87528 | F | 33 | 113488 | 4.50E-07 |
| *O. sinensis* | 33 | 108945 | F | 33 | 146493 | 1.39E-05 |
| *O. sinensis* | 33 | 9192 | F | 33 | 144474 | 1.39E-05 |
| *O. sinensis* | 33 | 13716 | F | 33 | 23728 | 1.39E-05 |
| *O. sinensis* | 33 | 129750 | F | 33 | 144625 | 1.39E-05 |
| *O. sinensis* | 33 | 27042 | F | 33 | 143381 | 9.37E-09 |
| *O. sinensis* | 33 | 17475 | F | 33 | 143381 | 9.37E-09 |
| *O. sinensis* | 33 | 9310 | F | 33 | 34563 | 4.50E-07 |
| *O. sinensis* | 33 | 87562 | F | 33 | 111784 | 1.39E-05 |
| *O. sinensis* | 33 | 87562 | F | 33 | 128875 | 1.39E-05 |
| *O. sinensis* | 33 | 135290 | F | 33 | 141155 | 4.50E-07 |
| *O. sinensis* | 33 | 141155 | F | 33 | 156388 | 4.50E-07 |
| *O. sinensis* | 33 | 135290 | F | 33 | 140304 | 4.50E-07 |
| *O. sinensis* | 33 | 140304 | F | 33 | 156388 | 4.50E-07 |
| *O. sinensis* | 33 | 23170 | F | 33 | 26491 | 1.39E-05 |
| *O. sinensis* | 33 | 113514 | F | 33 | 143367 | 1.39E-05 |
| *O. sinensis* | 33 | 55873 | F | 33 | 143367 | 1.39E-05 |
| *O. sinensis* | 33 | 58010 | F | 33 | 58046 | 9.46E-11 |
| *O. sinensis* | 33 | 29453 | F | 33 | 94179 | 1.39E-05 |
| *O. sinensis* | 33 | 28717 | F | 33 | 113514 | 4.50E-07 |
| *O. sinensis* | 33 | 28717 | F | 33 | 55873 | 4.50E-07 |
| *O. sinensis* | 33 | 17520 | F | 33 | 157066 | 4.50E-07 |
| *O. sinensis* | 33 | 57342 | F | 33 | 94530 | 1.39E-05 |
| *O. sinensis* | 33 | 44960 | F | 33 | 129756 | 1.39E-05 |
| *O. sinensis* | 33 | 70222 | F | 33 | 129756 | 1.39E-05 |
| *O. sinensis* | 33 | 42150 | F | 33 | 60698 | 1.39E-05 |
| *O. sinensis* | 33 | 3866 | F | 33 | 5472 | 1.39E-05 |
| *O. sinensis* | 33 | 6974 | F | 33 | 60694 | 1.39E-05 |
| *O. sinensis* | 33 | 87566 | F | 33 | 120795 | 1.39E-05 |
| *O. sinensis* | 33 | 110055 | F | 33 | 139030 | 4.50E-07 |
| *O. sinensis* | 33 | 24403 | F | 33 | 139030 | 4.50E-07 |
| *O. sinensis* | 33 | 139030 | F | 33 | 150581 | 4.50E-07 |
| *O. sinensis* | 33 | 139030 | F | 33 | 156080 | 4.50E-07 |
| *O. sinensis* | 33 | 126949 | F | 33 | 139030 | 4.50E-07 |
| *O. sinensis* | 33 | 24448 | F | 33 | 151473 | 1.39E-05 |
| *O. sinensis* | 33 | 150626 | F | 33 | 151473 | 1.39E-05 |
| *O. sinensis* | 33 | 27536 | P | 33 | 157106 | 1.39E-05 |
| *O. sinensis* | 33 | 84240 | P | 33 | 157066 | 4.50E-07 |
| *O. sinensis* | 33 | 29955 | P | 33 | 155335 | 1.39E-05 |
| *O. sinensis* | 33 | 151499 | P | 33 | 151499 | 1.39E-05 |
| *O. sinensis* | 33 | 126621 | P | 33 | 150626 | 1.39E-05 |
| *O. sinensis* | 33 | 17394 | P | 33 | 150227 | 1.39E-05 |
| *O. sinensis* | 33 | 86085 | P | 33 | 150227 | 1.39E-05 |
| *O. sinensis* | 33 | 27066 | P | 33 | 150122 | 1.39E-05 |
| *O. sinensis* | 33 | 16469 | P | 33 | 139988 | 4.50E-07 |
| *O. sinensis* | 33 | 128852 | P | 33 | 139988 | 4.50E-07 |
| *O. sinensis* | 33 | 111761 | P | 33 | 139988 | 4.50E-07 |
| *O. sinensis* | 33 | 56904 | P | 33 | 135376 | 4.50E-07 |
| *O. sinensis* | 33 | 16476 | P | 33 | 135165 | 1.39E-05 |
| *O. sinensis* | 33 | 121893 | P | 33 | 126621 | 9.46E-11 |
| *O. sinensis* | 33 | 24448 | P | 33 | 126621 | 1.39E-05 |
| *O. sinensis* | 33 | 121584 | P | 33 | 121893 | 1.39E-05 |
| *O. sinensis* | 33 | 108683 | P | 33 | 121497 | 1.39E-05 |
| *O. sinensis* | 33 | 94228 | P | 33 | 121492 | 1.39E-05 |
| *O. sinensis* | 33 | 108658 | P | 33 | 120795 | 9.37E-09 |
| *O. sinensis* | 33 | 28717 | P | 33 | 120787 | 1.39E-05 |
| *O. sinensis* | 33 | 87578 | P | 33 | 113494 | 1.39E-05 |
| *O. sinensis* | 33 | 55895 | P | 33 | 113364 | 1.39E-05 |
| *O. sinensis* | 33 | 94226 | P | 33 | 111760 | 4.50E-07 |
| *O. sinensis* | 33 | 21242 | P | 33 | 110203 | 1.39E-05 |
| *O. sinensis* | 33 | 13727 | P | 33 | 94226 | 4.50E-07 |
| *O. sinensis* | 33 | 16528 | P | 33 | 94165 | 9.37E-09 |
| *O. sinensis* | 33 | 16476 | P | 33 | 87576 | 1.39E-05 |
| *O. sinensis* | 33 | 87561 | P | 33 | 87561 | 1.39E-05 |
| *O. sinensis* | 33 | 13779 | P | 33 | 87539 | 9.46E-11 |
| *O. sinensis* | 33 | 76645 | P | 33 | 76645 | 1.39E-05 |
| *O. sinensis* | 33 | 55804 | R | 33 | 55804 | 4.50E-07 |
| *O. sinensis* | 33 | 121539 | R | 33 | 121539 | 9.46E-11 |
| *O. sinensis* | 33 | 146551 | R | 33 | 121539 | 9.46E-11 |
| *O. sinensis* | 33 | 146551 | R | 33 | 146551 | 9.46E-11 |
| *O. sinensis* | 32 | 34558 | F | 32 | 60654 | 5.07E-05 |
| *O. sinensis* | 32 | 57002 | F | 32 | 86177 | 3.63E-08 |
| *O. sinensis* | 32 | 56268 | F | 32 | 141215 | 3.63E-08 |
| *O. sinensis* | 32 | 94228 | F | 32 | 156166 | 5.07E-05 |
| *O. sinensis* | 32 | 87590 | F | 32 | 94232 | 5.07E-05 |
| *O. sinensis* | 32 | 94230 | F | 32 | 157099 | 1.69E-06 |
| *O. sinensis* | 32 | 45307 | F | 32 | 70683 | 5.07E-05 |
| *O. sinensis* | 32 | 121491 | F | 32 | 150047 | 5.07E-05 |
| *O. sinensis* | 32 | 55477 | F | 32 | 121491 | 5.07E-05 |
| *O. sinensis* | 32 | 3950 | F | 32 | 51825 | 5.07E-05 |
| *O. sinensis* | 32 | 7420 | F | 32 | 61170 | 5.07E-05 |
| *O. sinensis* | 32 | 70275 | F | 32 | 129796 | 5.07E-05 |
| *O. sinensis* | 32 | 45013 | F | 32 | 129796 | 5.07E-05 |
| *O. sinensis* | 32 | 9189 | F | 32 | 30313 | 5.07E-05 |
| *O. sinensis* | 32 | 9314 | F | 32 | 30437 | 1.69E-06 |
| *O. sinensis* | 32 | 16467 | F | 32 | 121493 | 5.07E-05 |
| *O. sinensis* | 32 | 16475 | F | 32 | 128858 | 3.63E-08 |
| *O. sinensis* | 32 | 120909 | F | 32 | 121491 | 3.63E-08 |
| *O. sinensis* | 32 | 55800 | F | 32 | 126020 | 5.07E-05 |
| *O. sinensis* | 32 | 92758 | F | 32 | 94231 | 3.63E-08 |
| *O. sinensis* | 32 | 7291 | F | 32 | 16781 | 5.07E-05 |
| *O. sinensis* | 32 | 63143 | F | 32 | 63251 | 1.69E-06 |
| *O. sinensis* | 32 | 30862 | F | 32 | 55763 | 3.63E-08 |
| *O. sinensis* | 32 | 30834 | F | 32 | 30862 | 3.63E-08 |
| *O. sinensis* | 32 | 18837 | F | 32 | 27033 | 5.07E-05 |
| *O. sinensis* | 32 | 17466 | F | 32 | 18837 | 5.07E-05 |
| *O. sinensis* | 32 | 16528 | F | 32 | 56984 | 3.78E-10 |
| *O. sinensis* | 32 | 56984 | F | 32 | 109737 | 1.69E-06 |
| *O. sinensis* | 32 | 27578 | F | 32 | 55887 | 5.07E-05 |
| *O. sinensis* | 32 | 108680 | F | 32 | 135167 | 3.63E-08 |
| *O. sinensis* | 32 | 108680 | F | 32 | 141207 | 3.63E-08 |
| *O. sinensis* | 32 | 108680 | F | 32 | 126128 | 3.63E-08 |
| *O. sinensis* | 32 | 108680 | F | 32 | 140356 | 3.63E-08 |
| *O. sinensis* | 32 | 5634 | F | 32 | 51919 | 5.07E-05 |
| *O. sinensis* | 32 | 16452 | F | 32 | 121878 | 5.07E-05 |
| *O. sinensis* | 32 | 34512 | F | 32 | 70125 | 5.07E-05 |
| *O. sinensis* | 32 | 34512 | F | 32 | 44862 | 5.07E-05 |
| *O. sinensis* | 32 | 58857 | F | 32 | 94531 | 3.63E-08 |
| *O. sinensis* | 32 | 30385 | F | 32 | 60610 | 5.07E-05 |
| *O. sinensis* | 32 | 71180 | F | 32 | 134107 | 1.69E-06 |
| *O. sinensis* | 32 | 4056 | F | 32 | 51937 | 1.69E-06 |
| *O. sinensis* | 32 | 25303 | F | 32 | 135146 | 5.07E-05 |
| *O. sinensis* | 32 | 18825 | F | 32 | 135146 | 5.07E-05 |
| *O. sinensis* | 32 | 18838 | F | 32 | 70375 | 5.07E-05 |
| *O. sinensis* | 32 | 141206 | F | 32 | 146587 | 5.07E-05 |
| *O. sinensis* | 32 | 126127 | F | 32 | 146587 | 5.07E-05 |
| *O. sinensis* | 32 | 140355 | F | 32 | 146587 | 5.07E-05 |
| *O. sinensis* | 32 | 126133 | F | 32 | 139988 | 5.07E-05 |
| *O. sinensis* | 32 | 27626 | F | 32 | 157145 | 1.69E-06 |
| *O. sinensis* | 32 | 29455 | F | 32 | 87560 | 1.69E-06 |
| *O. sinensis* | 32 | 40556 | F | 32 | 144631 | 3.63E-08 |
| *O. sinensis* | 32 | 109566 | F | 32 | 151910 | 5.07E-05 |
| *O. sinensis* | 32 | 25303 | F | 32 | 113507 | 3.63E-08 |
| *O. sinensis* | 32 | 25303 | F | 32 | 55866 | 3.63E-08 |
| *O. sinensis* | 32 | 25303 | F | 32 | 108659 | 5.07E-05 |
| *O. sinensis* | 32 | 18825 | F | 32 | 108659 | 5.07E-05 |
| *O. sinensis* | 32 | 13735 | F | 32 | 16476 | 5.07E-05 |
| *O. sinensis* | 32 | 70371 | F | 32 | 143369 | 5.07E-05 |
| *O. sinensis* | 32 | 24400 | F | 32 | 126946 | 1.69E-06 |
| *O. sinensis* | 32 | 55505 | P | 32 | 157071 | 3.63E-08 |
| *O. sinensis* | 32 | 150075 | P | 32 | 157071 | 3.63E-08 |
| *O. sinensis* | 32 | 16467 | P | 32 | 156166 | 5.07E-05 |
| *O. sinensis* | 32 | 128850 | P | 32 | 156166 | 5.07E-05 |
| *O. sinensis* | 32 | 24466 | P | 32 | 156149 | 5.07E-05 |
| *O. sinensis* | 32 | 16467 | P | 32 | 150664 | 5.07E-05 |
| *O. sinensis* | 32 | 128850 | P | 32 | 150664 | 5.07E-05 |
| *O. sinensis* | 32 | 24466 | P | 32 | 150647 | 5.07E-05 |
| *O. sinensis* | 32 | 113403 | P | 32 | 150625 | 5.07E-05 |
| *O. sinensis* | 32 | 16470 | P | 32 | 146593 | 5.07E-05 |
| *O. sinensis* | 32 | 128853 | P | 32 | 146593 | 5.07E-05 |
| *O. sinensis* | 32 | 111762 | P | 32 | 146593 | 5.07E-05 |
| *O. sinensis* | 32 | 92770 | P | 32 | 146489 | 1.69E-06 |
| *O. sinensis* | 32 | 144862 | P | 32 | 144862 | 3.78E-10 |
| *O. sinensis* | 32 | 108659 | P | 32 | 143369 | 3.78E-10 |
| *O. sinensis* | 32 | 25303 | P | 32 | 143369 | 5.07E-05 |
| *O. sinensis* | 32 | 26982 | P | 32 | 140362 | 3.63E-08 |
| *O. sinensis* | 32 | 27066 | P | 32 | 135269 | 1.69E-06 |
| *O. sinensis* | 32 | 128866 | P | 32 | 135159 | 5.07E-05 |
| *O. sinensis* | 32 | 111775 | P | 32 | 135159 | 5.07E-05 |
| *O. sinensis* | 32 | 13792 | P | 32 | 135126 | 5.07E-05 |
| *O. sinensis* | 32 | 17561 | P | 32 | 128850 | 1.69E-06 |
| *O. sinensis* | 32 | 87539 | P | 32 | 121988 | 5.07E-05 |
| *O. sinensis* | 32 | 113403 | P | 32 | 121892 | 1.69E-06 |
| *O. sinensis* | 32 | 109854 | P | 32 | 121491 | 5.07E-05 |
| *O. sinensis* | 32 | 17563 | P | 32 | 120909 | 1.69E-06 |
| *O. sinensis* | 32 | 27626 | P | 32 | 120866 | 3.63E-08 |
| *O. sinensis* | 32 | 25303 | P | 32 | 120795 | 1.69E-06 |
| *O. sinensis* | 32 | 113528 | P | 32 | 120774 | 3.78E-10 |
| *O. sinensis* | 32 | 70371 | P | 32 | 113507 | 5.07E-05 |
| *O. sinensis* | 32 | 28731 | P | 32 | 113495 | 1.69E-06 |
| *O. sinensis* | 32 | 24447 | P | 32 | 113403 | 5.07E-05 |
| *O. sinensis* | 32 | 109855 | P | 32 | 113371 | 3.63E-08 |
| *O. sinensis* | 32 | 108026 | P | 32 | 110812 | 3.63E-08 |
| *O. sinensis* | 32 | 27042 | P | 32 | 108647 | 3.78E-10 |
| *O. sinensis* | 32 | 17475 | P | 32 | 108647 | 3.78E-10 |
| *O. sinensis* | 32 | 14208 | P | 32 | 108033 | 5.07E-05 |
| *O. sinensis* | 32 | 13735 | P | 32 | 87577 | 5.07E-05 |
| *O. sinensis* | 32 | 55866 | P | 32 | 70371 | 5.07E-05 |
| *O. sinensis* | 32 | 55806 | P | 32 | 55806 | 1.69E-06 |
| *O. sinensis* | 32 | 25303 | P | 32 | 28719 | 1.69E-06 |
| *O. sinensis* | 32 | 16467 | P | 32 | 17561 | 1.69E-06 |
| *O. sinensis* | 32 | 55767 | R | 32 | 30862 | 3.63E-08 |
| *O. sinensis* | 32 | 80027 | R | 32 | 80034 | 5.07E-05 |
| *O. sinensis* | 32 | 155427 | R | 32 | 155427 | 1.69E-06 |
| *O. sinensis* | 32 | 155439 | R | 32 | 155444 | 5.07E-05 |
| *O. sinensis* | 32 | 121940 | R | 32 | 121539 | 1.69E-06 |
| *O. sinensis* | 32 | 121940 | R | 32 | 146551 | 1.69E-06 |
| *O. sinensis* | 32 | 30862 | C | 32 | 98108 | 3.63E-08 |
| *O. sinensis* | 32 | 30862 | C | 32 | 23491 | 5.07E-05 |
| *O. sinensis* | 31 | 140375 | F | 31 | 141228 | 1.84E-04 |
| *O. sinensis* | 31 | 18854 | F | 31 | 87603 | 6.33E-06 |
| *O. sinensis* | 31 | 113403 | F | 31 | 121586 | 1.84E-04 |
| *O. sinensis* | 31 | 87590 | F | 31 | 92759 | 1.84E-04 |
| *O. sinensis* | 31 | 92758 | F | 31 | 157100 | 6.33E-06 |
| *O. sinensis* | 31 | 70412 | F | 31 | 109022 | 1.51E-09 |
| *O. sinensis* | 31 | 113372 | F | 31 | 121491 | 6.33E-06 |
| *O. sinensis* | 31 | 113403 | F | 31 | 126623 | 6.33E-06 |
| *O. sinensis* | 31 | 17563 | F | 31 | 157099 | 6.33E-06 |
| *O. sinensis* | 31 | 60942 | F | 31 | 127986 | 1.84E-04 |
| *O. sinensis* | 31 | 94226 | F | 31 | 146594 | 1.84E-04 |
| *O. sinensis* | 31 | 126134 | F | 31 | 135356 | 1.84E-04 |
| *O. sinensis* | 31 | 13790 | F | 31 | 27048 | 1.84E-04 |
| *O. sinensis* | 31 | 13790 | F | 31 | 17481 | 1.84E-04 |
| *O. sinensis* | 31 | 28735 | F | 31 | 121589 | 1.84E-04 |
| *O. sinensis* | 31 | 6888 | F | 31 | 9306 | 1.41E-07 |
| *O. sinensis* | 31 | 31085 | F | 31 | 128350 | 1.84E-04 |
| *O. sinensis* | 31 | 4365 | F | 31 | 137491 | 1.84E-04 |
| *O. sinensis* | 31 | 14392 | F | 31 | 121590 | 1.84E-04 |
| *O. sinensis* | 31 | 92539 | F | 31 | 123654 | 1.41E-07 |
| *O. sinensis* | 31 | 9306 | F | 31 | 129705 | 1.84E-04 |
| *O. sinensis* | 31 | 109823 | F | 31 | 121892 | 6.33E-06 |
| *O. sinensis* | 31 | 87528 | F | 31 | 121886 | 1.84E-04 |
| *O. sinensis* | 31 | 83280 | F | 31 | 83334 | 1.41E-07 |
| *O. sinensis* | 31 | 84239 | F | 31 | 113403 | 1.84E-04 |
| *O. sinensis* | 31 | 17567 | F | 31 | 150670 | 1.84E-04 |
| *O. sinensis* | 31 | 150666 | F | 31 | 157099 | 1.84E-04 |
| *O. sinensis* | 31 | 5495 | F | 31 | 48128 | 1.84E-04 |
| *O. sinensis* | 31 | 55763 | F | 31 | 55770 | 1.41E-07 |
| *O. sinensis* | 31 | 30834 | F | 31 | 55770 | 1.41E-07 |
| *O. sinensis* | 31 | 147560 | F | 31 | 147591 | 1.84E-04 |
| *O. sinensis* | 31 | 26099 | F | 31 | 147591 | 1.84E-04 |
| *O. sinensis* | 31 | 14151 | F | 31 | 147560 | 1.84E-04 |
| *O. sinensis* | 31 | 14151 | F | 31 | 26099 | 1.84E-04 |
| *O. sinensis* | 31 | 21173 | F | 31 | 142510 | 6.33E-06 |
| *O. sinensis* | 31 | 41350 | F | 31 | 144949 | 1.84E-04 |
| *O. sinensis* | 31 | 9306 | F | 31 | 60655 | 1.84E-04 |
| *O. sinensis* | 31 | 129673 | F | 31 | 144557 | 6.33E-06 |
| *O. sinensis* | 31 | 14211 | F | 31 | 28071 | 1.51E-09 |
| *O. sinensis* | 31 | 14322 | F | 31 | 135100 | 6.33E-06 |
| *O. sinensis* | 31 | 139986 | F | 31 | 146591 | 1.84E-04 |
| *O. sinensis* | 31 | 23533 | F | 31 | 126694 | 6.33E-06 |
| *O. sinensis* | 31 | 30479 | F | 31 | 39092 | 1.84E-04 |
| *O. sinensis* | 31 | 39092 | F | 31 | 42149 | 1.84E-04 |
| *O. sinensis* | 31 | 109809 | F | 31 | 157051 | 1.84E-04 |
| *O. sinensis* | 31 | 71563 | F | 31 | 71581 | 6.33E-06 |
| *O. sinensis* | 31 | 25304 | F | 31 | 143361 | 1.84E-04 |
| *O. sinensis* | 31 | 27034 | F | 31 | 70375 | 1.84E-04 |
| *O. sinensis* | 31 | 17467 | F | 31 | 70375 | 1.84E-04 |
| *O. sinensis* | 31 | 27566 | F | 31 | 29461 | 1.84E-04 |
| *O. sinensis* | 31 | 56310 | F | 31 | 118449 | 1.84E-04 |
| *O. sinensis* | 31 | 25304 | F | 31 | 109717 | 6.33E-06 |
| *O. sinensis* | 31 | 18826 | F | 31 | 109717 | 6.33E-06 |
| *O. sinensis* | 31 | 109717 | F | 31 | 120787 | 6.33E-06 |
| *O. sinensis* | 31 | 109717 | F | 31 | 113508 | 1.84E-04 |
| *O. sinensis* | 31 | 55867 | F | 31 | 109717 | 1.84E-04 |
| *O. sinensis* | 31 | 25304 | F | 31 | 28711 | 6.33E-06 |
| *O. sinensis* | 31 | 27558 | F | 31 | 109717 | 1.84E-04 |
| *O. sinensis* | 31 | 111758 | F | 31 | 150048 | 1.41E-07 |
| *O. sinensis* | 31 | 55478 | F | 31 | 111758 | 1.41E-07 |
| *O. sinensis* | 31 | 55687 | F | 31 | 111758 | 1.41E-07 |
| *O. sinensis* | 31 | 9193 | F | 31 | 129591 | 1.84E-04 |
| *O. sinensis* | 31 | 6787 | F | 31 | 30328 | 1.84E-04 |
| *O. sinensis* | 31 | 87521 | F | 31 | 128836 | 6.33E-06 |
| *O. sinensis* | 31 | 108931 | F | 31 | 156369 | 6.33E-06 |
| *O. sinensis* | 31 | 121536 | F | 31 | 121541 | 1.51E-09 |
| *O. sinensis* | 31 | 121536 | F | 31 | 146553 | 1.51E-09 |
| *O. sinensis* | 31 | 121541 | F | 31 | 146548 | 1.51E-09 |
| *O. sinensis* | 31 | 146548 | F | 31 | 146553 | 1.51E-09 |
| *O. sinensis* | 31 | 9351 | F | 31 | 60698 | 1.84E-04 |
| *O. sinensis* | 31 | 9351 | F | 31 | 44960 | 6.33E-06 |
| *O. sinensis* | 31 | 9351 | F | 31 | 70222 | 6.33E-06 |
| *O. sinensis* | 31 | 30480 | F | 31 | 60698 | 1.84E-04 |
| *O. sinensis* | 31 | 40556 | F | 31 | 129756 | 6.33E-06 |
| *O. sinensis* | 31 | 40556 | F | 31 | 44960 | 1.84E-04 |
| *O. sinensis* | 31 | 40556 | F | 31 | 70222 | 1.84E-04 |
| *O. sinensis* | 31 | 70350 | F | 31 | 121902 | 1.84E-04 |
| *O. sinensis* | 31 | 17532 | F | 31 | 70350 | 1.84E-04 |
| *O. sinensis* | 31 | 27558 | F | 31 | 108660 | 1.84E-04 |
| *O. sinensis* | 31 | 17532 | F | 31 | 121902 | 6.33E-06 |
| *O. sinensis* | 31 | 24447 | F | 31 | 109823 | 1.84E-04 |
| *O. sinensis* | 31 | 109823 | F | 31 | 150625 | 1.84E-04 |
| *O. sinensis* | 31 | 24457 | F | 31 | 121902 | 1.84E-04 |
| *O. sinensis* | 31 | 121902 | F | 31 | 150635 | 1.84E-04 |
| *O. sinensis* | 31 | 121902 | F | 31 | 156137 | 1.84E-04 |
| *O. sinensis* | 31 | 146513 | F | 31 | 151482 | 1.84E-04 |
| *O. sinensis* | 31 | 17532 | F | 31 | 146513 | 1.84E-04 |
| *O. sinensis* | 31 | 13735 | F | 31 | 111768 | 1.84E-04 |
| *O. sinensis* | 31 | 13735 | F | 31 | 128859 | 1.84E-04 |
| *O. sinensis* | 31 | 121536 | F | 31 | 121941 | 6.33E-06 |
| *O. sinensis* | 31 | 121941 | F | 31 | 146548 | 6.33E-06 |
| *O. sinensis* | 31 | 28188 | F | 31 | 70371 | 1.84E-04 |
| *O. sinensis* | 31 | 70371 | F | 31 | 157242 | 1.84E-04 |
| *O. sinensis* | 31 | 109722 | F | 31 | 111785 | 1.84E-04 |
| *O. sinensis* | 31 | 87560 | F | 31 | 113510 | 1.84E-04 |
| *O. sinensis* | 31 | 55869 | F | 31 | 87560 | 1.84E-04 |
| *O. sinensis* | 31 | 87563 | F | 31 | 109722 | 6.33E-06 |
| *O. sinensis* | 31 | 45093 | F | 31 | 70438 | 6.33E-06 |
| *O. sinensis* | 31 | 111758 | F | 31 | 120910 | 1.41E-07 |
| *O. sinensis* | 31 | 27538 | F | 31 | 150041 | 1.84E-04 |
| *O. sinensis* | 31 | 27538 | F | 31 | 55471 | 1.84E-04 |
| *O. sinensis* | 31 | 27542 | F | 31 | 113370 | 1.84E-04 |
| *O. sinensis* | 31 | 110053 | F | 31 | 126947 | 6.33E-06 |
| *O. sinensis* | 31 | 126947 | F | 31 | 150579 | 6.33E-06 |
| *O. sinensis* | 31 | 126947 | F | 31 | 156078 | 6.33E-06 |
| *O. sinensis* | 31 | 151472 | F | 31 | 157068 | 1.41E-07 |
| *O. sinensis* | 31 | 146503 | F | 31 | 157068 | 1.84E-04 |
| *O. sinensis* | 31 | 156124 | F | 31 | 157065 | 1.84E-04 |
| *O. sinensis* | 31 | 135147 | P | 31 | 157242 | 1.84E-04 |
| *O. sinensis* | 31 | 13790 | P | 31 | 157216 | 1.84E-04 |
| *O. sinensis* | 31 | 17483 | P | 31 | 157214 | 6.33E-06 |
| *O. sinensis* | 31 | 27050 | P | 31 | 157214 | 6.33E-06 |
| *O. sinensis* | 31 | 121492 | P | 31 | 157099 | 1.41E-07 |
| *O. sinensis* | 31 | 111758 | P | 31 | 157099 | 1.41E-07 |
| *O. sinensis* | 31 | 126624 | P | 31 | 157068 | 1.41E-07 |
| *O. sinensis* | 31 | 86177 | P | 31 | 156375 | 1.84E-04 |
| *O. sinensis* | 31 | 111760 | P | 31 | 156166 | 1.84E-04 |
| *O. sinensis* | 31 | 121593 | P | 31 | 156121 | 1.84E-04 |
| *O. sinensis* | 31 | 152148 | P | 31 | 155461 | 1.84E-04 |
| *O. sinensis* | 31 | 30058 | P | 31 | 155234 | 1.41E-07 |
| *O. sinensis* | 31 | 113403 | P | 31 | 151473 | 6.33E-06 |
| *O. sinensis* | 31 | 28737 | P | 31 | 151468 | 1.41E-07 |
| *O. sinensis* | 31 | 120910 | P | 31 | 150666 | 1.84E-04 |
| *O. sinensis* | 31 | 111760 | P | 31 | 150664 | 1.84E-04 |
| *O. sinensis* | 31 | 120941 | P | 31 | 150625 | 1.84E-04 |
| *O. sinensis* | 31 | 55718 | P | 31 | 150625 | 1.84E-04 |
| *O. sinensis* | 31 | 121892 | P | 31 | 150079 | 1.84E-04 |
| *O. sinensis* | 31 | 55895 | P | 31 | 150041 | 6.33E-06 |
| *O. sinensis* | 31 | 21253 | P | 31 | 148737 | 1.84E-04 |
| *O. sinensis* | 31 | 108091 | P | 31 | 147560 | 1.84E-04 |
| *O. sinensis* | 31 | 135168 | P | 31 | 146513 | 1.84E-04 |
| *O. sinensis* | 31 | 128859 | P | 31 | 141207 | 6.33E-06 |
| *O. sinensis* | 31 | 111768 | P | 31 | 141207 | 6.33E-06 |
| *O. sinensis* | 31 | 128859 | P | 31 | 140356 | 6.33E-06 |
| *O. sinensis* | 31 | 111768 | P | 31 | 140356 | 6.33E-06 |
| *O. sinensis* | 31 | 86177 | P | 31 | 135277 | 1.84E-04 |
| *O. sinensis* | 31 | 28188 | P | 31 | 135147 | 1.84E-04 |
| *O. sinensis* | 31 | 28731 | P | 31 | 135135 | 6.33E-06 |
| *O. sinensis* | 31 | 126128 | P | 31 | 128859 | 6.33E-06 |
| *O. sinensis* | 31 | 92756 | P | 31 | 128850 | 1.84E-04 |
| *O. sinensis* | 31 | 17416 | P | 31 | 126134 | 1.84E-04 |
| *O. sinensis* | 31 | 111768 | P | 31 | 126128 | 6.33E-06 |
| *O. sinensis* | 31 | 109742 | P | 31 | 126078 | 1.51E-09 |
| *O. sinensis* | 31 | 55718 | P | 31 | 121892 | 6.33E-06 |
| *O. sinensis* | 31 | 120941 | P | 31 | 121892 | 6.33E-06 |
| *O. sinensis* | 31 | 55509 | P | 31 | 121892 | 1.84E-04 |
| *O. sinensis* | 31 | 24447 | P | 31 | 120941 | 1.84E-04 |
| *O. sinensis* | 31 | 87588 | P | 31 | 120910 | 1.84E-04 |
| *O. sinensis* | 31 | 94230 | P | 31 | 120910 | 6.33E-06 |
| *O. sinensis* | 31 | 29461 | P | 31 | 120787 | 1.84E-04 |
| *O. sinensis* | 31 | 117223 | P | 31 | 117223 | 1.41E-07 |
| *O. sinensis* | 31 | 108660 | P | 31 | 113516 | 1.84E-04 |
| *O. sinensis* | 31 | 109717 | P | 31 | 113516 | 1.84E-04 |
| *O. sinensis* | 31 | 17523 | P | 31 | 113403 | 1.84E-04 |
| *O. sinensis* | 31 | 17564 | P | 31 | 113372 | 1.84E-04 |
| *O. sinensis* | 31 | 17561 | P | 31 | 111760 | 6.33E-06 |
| *O. sinensis* | 31 | 109854 | P | 31 | 111758 | 1.41E-07 |
| *O. sinensis* | 31 | 24285 | P | 31 | 110740 | 1.84E-04 |
| *O. sinensis* | 31 | 27566 | P | 31 | 109717 | 6.33E-06 |
| *O. sinensis* | 31 | 55875 | P | 31 | 109717 | 1.84E-04 |
| *O. sinensis* | 31 | 92768 | P | 31 | 108944 | 1.84E-04 |
| *O. sinensis* | 31 | 27531 | P | 31 | 108705 | 1.84E-04 |
| *O. sinensis* | 31 | 16476 | P | 31 | 108680 | 6.33E-06 |
| *O. sinensis* | 31 | 87562 | P | 31 | 108664 | 6.33E-06 |
| *O. sinensis* | 31 | 55875 | P | 31 | 108660 | 1.84E-04 |
| *O. sinensis* | 31 | 26099 | P | 31 | 108091 | 1.84E-04 |
| *O. sinensis* | 31 | 30863 | P | 31 | 98154 | 1.41E-07 |
| *O. sinensis* | 31 | 16465 | P | 31 | 92758 | 1.84E-04 |
| *O. sinensis* | 31 | 29456 | P | 31 | 87563 | 1.84E-04 |
| *O. sinensis* | 31 | 28711 | P | 31 | 70371 | 1.84E-04 |
| *O. sinensis* | 31 | 55471 | P | 31 | 55895 | 6.33E-06 |
| *O. sinensis* | 31 | 28731 | P | 31 | 55855 | 1.41E-07 |
| *O. sinensis* | 31 | 13790 | P | 31 | 55849 | 6.33E-06 |
| *O. sinensis* | 31 | 24447 | P | 31 | 55718 | 1.84E-04 |
| *O. sinensis* | 31 | 23495 | P | 31 | 30863 | 1.84E-04 |
| *O. sinensis* | 31 | 25304 | P | 31 | 29461 | 1.84E-04 |
| *O. sinensis* | 31 | 18826 | P | 31 | 29461 | 1.84E-04 |
| *O. sinensis* | 31 | 13790 | P | 31 | 28162 | 1.84E-04 |
| *O. sinensis* | 31 | 17483 | P | 31 | 28160 | 1.41E-07 |
| *O. sinensis* | 31 | 27050 | P | 31 | 28160 | 1.41E-07 |
| *O. sinensis* | 31 | 25301 | P | 31 | 27033 | 6.33E-06 |
| *O. sinensis* | 31 | 18823 | P | 31 | 27033 | 6.33E-06 |
| *O. sinensis* | 31 | 17466 | P | 31 | 25301 | 6.33E-06 |
| *O. sinensis* | 31 | 14120 | P | 31 | 23043 | 1.84E-04 |
| *O. sinensis* | 31 | 14089 | P | 31 | 23043 | 1.84E-04 |
| *O. sinensis* | 31 | 17466 | P | 31 | 18823 | 6.33E-06 |
| *O. sinensis* | 31 | 13727 | P | 31 | 17561 | 1.84E-04 |
| *O. sinensis* | 31 | 55157 | R | 31 | 55161 | 1.84E-04 |
| *O. sinensis* | 31 | 98156 | R | 31 | 98156 | 6.33E-06 |
| *O. sinensis* | 31 | 55768 | R | 31 | 55770 | 1.41E-07 |
| *O. sinensis* | 31 | 155419 | R | 31 | 155421 | 1.41E-07 |
| *O. sinensis* | 31 | 55804 | C | 31 | 55807 | 6.33E-06 |
| *O. sinensis* | 31 | 55770 | C | 31 | 98108 | 1.41E-07 |
| *O. sinensis* | 31 | 55770 | C | 31 | 23491 | 1.84E-04 |
| *O. sinensis* | 30 | 126153 | F | 30 | 140380 | 6.64E-04 |
| *O. sinensis* | 30 | 94254 | F | 30 | 135375 | 2.37E-05 |
| *O. sinensis* | 30 | 41127 | F | 30 | 112488 | 6.64E-04 |
| *O. sinensis* | 30 | 108688 | F | 30 | 141215 | 6.64E-04 |
| *O. sinensis* | 30 | 56265 | F | 30 | 126133 | 6.64E-04 |
| *O. sinensis* | 30 | 56266 | F | 30 | 140362 | 6.64E-04 |
| *O. sinensis* | 30 | 141213 | F | 30 | 156454 | 6.64E-04 |
| *O. sinensis* | 30 | 140364 | F | 30 | 156456 | 5.45E-07 |
| *O. sinensis* | 30 | 17561 | F | 30 | 139991 | 2.37E-05 |
| *O. sinensis* | 30 | 88681 | F | 30 | 112931 | 2.37E-05 |
| *O. sinensis* | 30 | 121587 | F | 30 | 150079 | 2.37E-05 |
| *O. sinensis* | 30 | 55509 | F | 30 | 121587 | 2.37E-05 |
| *O. sinensis* | 30 | 28737 | F | 30 | 126628 | 5.45E-07 |
| *O. sinensis* | 30 | 120941 | F | 30 | 121587 | 6.64E-04 |
| *O. sinensis* | 30 | 55718 | F | 30 | 121587 | 6.64E-04 |
| *O. sinensis* | 30 | 17564 | F | 30 | 92758 | 6.64E-04 |
| *O. sinensis* | 30 | 55891 | F | 30 | 157102 | 6.64E-04 |
| *O. sinensis* | 30 | 113532 | F | 30 | 157102 | 6.64E-04 |
| *O. sinensis* | 30 | 55802 | F | 30 | 55808 | 2.37E-05 |
| *O. sinensis* | 30 | 28773 | F | 30 | 109022 | 6.64E-04 |
| *O. sinensis* | 30 | 109021 | F | 30 | 128942 | 6.64E-04 |
| *O. sinensis* | 30 | 44339 | F | 30 | 129171 | 6.64E-04 |
| *O. sinensis* | 30 | 8421 | F | 30 | 8470 | 6.64E-04 |
| *O. sinensis* | 30 | 21255 | F | 30 | 126632 | 6.64E-04 |
| *O. sinensis* | 30 | 127399 | F | 30 | 137490 | 5.45E-07 |
| *O. sinensis* | 30 | 56077 | F | 30 | 78461 | 2.37E-05 |
| *O. sinensis* | 30 | 43995 | F | 30 | 81144 | 6.64E-04 |
| *O. sinensis* | 30 | 44916 | F | 30 | 129711 | 2.37E-05 |
| *O. sinensis* | 30 | 70178 | F | 30 | 129711 | 2.37E-05 |
| *O. sinensis* | 30 | 60660 | F | 30 | 144591 | 2.37E-05 |
| *O. sinensis* | 30 | 60661 | F | 30 | 129711 | 6.64E-04 |
| *O. sinensis* | 30 | 55688 | F | 30 | 128850 | 2.37E-05 |
| *O. sinensis* | 30 | 139993 | F | 30 | 157099 | 6.64E-04 |
| *O. sinensis* | 30 | 137965 | F | 30 | 145740 | 6.64E-04 |
| *O. sinensis* | 30 | 30478 | F | 30 | 42148 | 6.64E-04 |
| *O. sinensis* | 30 | 9349 | F | 30 | 42148 | 6.64E-04 |
| *O. sinensis* | 30 | 94226 | F | 30 | 141213 | 6.64E-04 |
| *O. sinensis* | 30 | 135355 | F | 30 | 141212 | 6.64E-04 |
| *O. sinensis* | 30 | 141213 | F | 30 | 150208 | 6.64E-04 |
| *O. sinensis* | 30 | 140362 | F | 30 | 150208 | 6.64E-04 |
| *O. sinensis* | 30 | 121588 | F | 30 | 143384 | 6.64E-04 |
| *O. sinensis* | 30 | 27046 | F | 30 | 121589 | 6.64E-04 |
| *O. sinensis* | 30 | 17479 | F | 30 | 121589 | 6.64E-04 |
| *O. sinensis* | 30 | 92759 | F | 30 | 113531 | 6.64E-04 |
| *O. sinensis* | 30 | 94232 | F | 30 | 113531 | 6.64E-04 |
| *O. sinensis* | 30 | 70497 | F | 30 | 129855 | 6.64E-04 |
| *O. sinensis* | 30 | 35370 | F | 30 | 112797 | 6.64E-04 |
| *O. sinensis* | 30 | 4361 | F | 30 | 127396 | 6.64E-04 |
| *O. sinensis* | 30 | 56266 | F | 30 | 108686 | 6.64E-04 |
| *O. sinensis* | 30 | 23796 | F | 30 | 121594 | 6.64E-04 |
| *O. sinensis* | 30 | 35142 | F | 30 | 51123 | 6.64E-04 |
| *O. sinensis* | 30 | 14587 | F | 30 | 18092 | 2.37E-05 |
| *O. sinensis* | 30 | 93688 | F | 30 | 142173 | 6.64E-04 |
| *O. sinensis* | 30 | 16085 | F | 30 | 107969 | 2.37E-05 |
| *O. sinensis* | 30 | 37656 | F | 30 | 76783 | 6.64E-04 |
| *O. sinensis* | 30 | 70351 | F | 30 | 121502 | 6.64E-04 |
| *O. sinensis* | 30 | 16479 | F | 30 | 70354 | 6.64E-04 |
| *O. sinensis* | 30 | 109824 | F | 30 | 151473 | 2.37E-05 |
| *O. sinensis* | 30 | 16468 | F | 30 | 23739 | 6.64E-04 |
| *O. sinensis* | 30 | 126020 | F | 30 | 157169 | 6.64E-04 |
| *O. sinensis* | 30 | 17244 | F | 30 | 122819 | 2.37E-05 |
| *O. sinensis* | 30 | 26166 | F | 30 | 122819 | 6.64E-04 |
| *O. sinensis* | 30 | 122819 | F | 30 | 147625 | 6.64E-04 |
| *O. sinensis* | 30 | 13789 | F | 30 | 121590 | 6.64E-04 |
| *O. sinensis* | 30 | 45860 | F | 30 | 59639 | 6.64E-04 |
| *O. sinensis* | 30 | 120941 | F | 30 | 126624 | 2.37E-05 |
| *O. sinensis* | 30 | 55718 | F | 30 | 126624 | 2.37E-05 |
| *O. sinensis* | 30 | 126624 | F | 30 | 150079 | 6.64E-04 |
| *O. sinensis* | 30 | 55509 | F | 30 | 126624 | 6.64E-04 |
| *O. sinensis* | 30 | 84240 | F | 30 | 120941 | 6.64E-04 |
| *O. sinensis* | 30 | 55718 | F | 30 | 84240 | 6.64E-04 |
| *O. sinensis* | 30 | 139991 | F | 30 | 150664 | 6.64E-04 |
| *O. sinensis* | 30 | 139991 | F | 30 | 156166 | 6.64E-04 |
| *O. sinensis* | 30 | 156168 | F | 30 | 157099 | 6.64E-04 |
| *O. sinensis* | 30 | 122526 | F | 30 | 122592 | 6.64E-04 |
| *O. sinensis* | 30 | 26973 | F | 30 | 146489 | 6.64E-04 |
| *O. sinensis* | 30 | 55784 | F | 30 | 113449 | 5.45E-07 |
| *O. sinensis* | 30 | 14630 | F | 30 | 18126 | 2.37E-05 |
| *O. sinensis* | 30 | 28154 | F | 30 | 29418 | 5.45E-07 |
| *O. sinensis* | 30 | 29418 | F | 30 | 157208 | 2.37E-05 |
| *O. sinensis* | 30 | 87576 | F | 30 | 141205 | 6.64E-04 |
| *O. sinensis* | 30 | 87576 | F | 30 | 126126 | 6.64E-04 |
| *O. sinensis* | 30 | 87576 | F | 30 | 140354 | 6.64E-04 |
| *O. sinensis* | 30 | 123445 | F | 30 | 147923 | 6.64E-04 |
| *O. sinensis* | 30 | 108933 | F | 30 | 150127 | 5.45E-07 |
| *O. sinensis* | 30 | 108932 | F | 30 | 135272 | 6.64E-04 |
| *O. sinensis* | 30 | 150129 | F | 30 | 156373 | 6.64E-04 |
| *O. sinensis* | 30 | 83029 | F | 30 | 129220 | 5.45E-07 |
| *O. sinensis* | 30 | 85378 | F | 30 | 91736 | 6.64E-04 |
| *O. sinensis* | 30 | 98108 | F | 30 | 98157 | 5.45E-07 |
| *O. sinensis* | 30 | 23491 | F | 30 | 98157 | 6.64E-04 |
| *O. sinensis* | 30 | 23498 | F | 30 | 98108 | 6.64E-04 |
| *O. sinensis* | 30 | 67711 | F | 30 | 67718 | 5.45E-07 |
| *O. sinensis* | 30 | 70366 | F | 30 | 87561 | 6.64E-04 |
| *O. sinensis* | 30 | 126058 | F | 30 | 141139 | 6.64E-04 |
| *O. sinensis* | 30 | 126058 | F | 30 | 140288 | 6.64E-04 |
| *O. sinensis* | 30 | 24377 | F | 30 | 150556 | 6.05E-09 |
| *O. sinensis* | 30 | 126922 | F | 30 | 150556 | 5.45E-07 |
| *O. sinensis* | 30 | 126922 | F | 30 | 156055 | 5.45E-07 |
| *O. sinensis* | 30 | 3585 | F | 30 | 138823 | 2.37E-05 |
| *O. sinensis* | 30 | 35283 | F | 30 | 81332 | 6.64E-04 |
| *O. sinensis* | 30 | 39092 | F | 30 | 60697 | 2.37E-05 |
| *O. sinensis* | 30 | 9350 | F | 30 | 39092 | 6.64E-04 |
| *O. sinensis* | 30 | 39088 | F | 30 | 129751 | 6.64E-04 |
| *O. sinensis* | 30 | 34622 | F | 30 | 39093 | 6.64E-04 |
| *O. sinensis* | 30 | 39139 | F | 30 | 42202 | 6.64E-04 |
| *O. sinensis* | 30 | 56109 | F | 30 | 78493 | 6.64E-04 |
| *O. sinensis* | 30 | 5747 | F | 30 | 127164 | 6.64E-04 |
| *O. sinensis* | 30 | 56946 | F | 30 | 86133 | 6.64E-04 |
| *O. sinensis* | 30 | 27004 | F | 30 | 86133 | 6.64E-04 |
| *O. sinensis* | 30 | 17437 | F | 30 | 86133 | 6.64E-04 |
| *O. sinensis* | 30 | 18127 | F | 30 | 76785 | 2.37E-05 |
| *O. sinensis* | 30 | 24529 | F | 30 | 110115 | 2.37E-05 |
| *O. sinensis* | 30 | 87590 | F | 30 | 157101 | 2.37E-05 |
| *O. sinensis* | 30 | 87563 | F | 30 | 143366 | 6.64E-04 |
| *O. sinensis* | 30 | 109722 | F | 30 | 135152 | 6.64E-04 |
| *O. sinensis* | 30 | 27563 | F | 30 | 28185 | 6.64E-04 |
| *O. sinensis* | 30 | 27563 | F | 30 | 157239 | 6.64E-04 |
| *O. sinensis* | 30 | 27562 | F | 30 | 143365 | 6.64E-04 |
| *O. sinensis* | 30 | 139988 | F | 30 | 141212 | 6.64E-04 |
| *O. sinensis* | 30 | 135272 | F | 30 | 150126 | 5.45E-07 |
| *O. sinensis* | 30 | 29451 | F | 30 | 135307 | 6.64E-04 |
| *O. sinensis* | 30 | 29451 | F | 30 | 156405 | 6.64E-04 |
| *O. sinensis* | 30 | 108665 | F | 30 | 109722 | 6.64E-04 |
| *O. sinensis* | 30 | 111758 | F | 30 | 113373 | 6.05E-09 |
| *O. sinensis* | 30 | 23737 | F | 30 | 150048 | 6.64E-04 |
| *O. sinensis* | 30 | 23737 | F | 30 | 55478 | 6.64E-04 |
| *O. sinensis* | 30 | 23737 | F | 30 | 113373 | 2.37E-05 |
| *O. sinensis* | 30 | 13725 | F | 30 | 113373 | 6.64E-04 |
| *O. sinensis* | 30 | 27543 | F | 30 | 120908 | 2.37E-05 |
| *O. sinensis* | 30 | 120772 | F | 30 | 120908 | 6.64E-04 |
| *O. sinensis* | 30 | 23737 | F | 30 | 55687 | 6.64E-04 |
| *O. sinensis* | 30 | 5491 | F | 30 | 51760 | 6.64E-04 |
| *O. sinensis* | 30 | 84928 | F | 30 | 86617 | 6.64E-04 |
| *O. sinensis* | 30 | 87556 | F | 30 | 135307 | 5.45E-07 |
| *O. sinensis* | 30 | 87556 | F | 30 | 156405 | 5.45E-07 |
| *O. sinensis* | 30 | 87556 | F | 30 | 94177 | 2.37E-05 |
| *O. sinensis* | 30 | 30436 | F | 30 | 34566 | 5.45E-07 |
| *O. sinensis* | 30 | 121532 | F | 30 | 121942 | 2.37E-05 |
| *O. sinensis* | 30 | 121942 | F | 30 | 146544 | 2.37E-05 |
| *O. sinensis* | 30 | 60693 | F | 30 | 144626 | 6.64E-04 |
| *O. sinensis* | 30 | 9384 | F | 30 | 70266 | 6.64E-04 |
| *O. sinensis* | 30 | 9384 | F | 30 | 45004 | 6.64E-04 |
| *O. sinensis* | 30 | 39967 | F | 30 | 112920 | 6.64E-04 |
| *O. sinensis* | 30 | 91386 | F | 30 | 143267 | 6.64E-04 |
| *O. sinensis* | 30 | 71189 | F | 30 | 134116 | 6.64E-04 |
| *O. sinensis* | 30 | 7062 | F | 30 | 34706 | 5.45E-07 |
| *O. sinensis* | 30 | 109837 | F | 30 | 113386 | 2.37E-05 |
| *O. sinensis* | 30 | 113386 | F | 30 | 157082 | 2.37E-05 |
| *O. sinensis* | 30 | 109837 | F | 30 | 120923 | 6.64E-04 |
| *O. sinensis* | 30 | 55700 | F | 30 | 109837 | 6.64E-04 |
| *O. sinensis* | 30 | 120923 | F | 30 | 157082 | 6.64E-04 |
| *O. sinensis* | 30 | 55700 | F | 30 | 157082 | 6.64E-04 |
| *O. sinensis* | 30 | 17523 | F | 30 | 109824 | 6.64E-04 |
| *O. sinensis* | 30 | 24445 | F | 30 | 87532 | 6.64E-04 |
| *O. sinensis* | 30 | 87532 | F | 30 | 150623 | 6.64E-04 |
| *O. sinensis* | 30 | 13735 | F | 30 | 121502 | 6.64E-04 |
| *O. sinensis* | 30 | 34695 | F | 30 | 60766 | 6.64E-04 |
| *O. sinensis* | 30 | 121937 | F | 30 | 121942 | 2.37E-05 |
| *O. sinensis* | 30 | 34621 | F | 30 | 60697 | 2.37E-05 |
| *O. sinensis* | 30 | 40551 | F | 30 | 60693 | 6.64E-04 |
| *O. sinensis* | 30 | 7050 | F | 30 | 39159 | 6.64E-04 |
| *O. sinensis* | 30 | 56217 | F | 30 | 87556 | 6.05E-09 |
| *O. sinensis* | 30 | 155414 | F | 30 | 155429 | 6.64E-04 |
| *O. sinensis* | 30 | 110202 | F | 30 | 126948 | 6.64E-04 |
| *O. sinensis* | 30 | 113373 | F | 30 | 128849 | 2.37E-05 |
| *O. sinensis* | 30 | 128850 | F | 30 | 150049 | 2.37E-05 |
| *O. sinensis* | 30 | 55479 | F | 30 | 128850 | 2.37E-05 |
| *O. sinensis* | 30 | 23737 | F | 30 | 120910 | 6.64E-04 |
| *O. sinensis* | 30 | 23739 | F | 30 | 128851 | 6.64E-04 |
| *O. sinensis* | 30 | 120768 | F | 30 | 150042 | 6.64E-04 |
| *O. sinensis* | 30 | 55472 | F | 30 | 120768 | 6.64E-04 |
| *O. sinensis* | 30 | 113367 | F | 30 | 120768 | 6.64E-04 |
| *O. sinensis* | 30 | 120911 | F | 30 | 128850 | 6.05E-09 |
| *O. sinensis* | 30 | 56203 | F | 30 | 141158 | 5.45E-07 |
| *O. sinensis* | 30 | 56203 | F | 30 | 140307 | 5.45E-07 |
| *O. sinensis* | 30 | 135135 | F | 30 | 157070 | 6.64E-04 |
| *O. sinensis* | 30 | 28168 | F | 30 | 157070 | 6.64E-04 |
| *O. sinensis* | 30 | 157070 | F | 30 | 157222 | 6.64E-04 |
| *O. sinensis* | 30 | 56203 | F | 30 | 126079 | 6.05E-09 |
| *O. sinensis* | 30 | 24448 | F | 30 | 157069 | 2.37E-05 |
| *O. sinensis* | 30 | 150626 | F | 30 | 157069 | 2.37E-05 |
| *O. sinensis* | 30 | 121893 | F | 30 | 157069 | 5.45E-07 |
| *O. sinensis* | 30 | 16467 | F | 30 | 120911 | 2.37E-05 |
| *O. sinensis* | 30 | 126079 | F | 30 | 135293 | 6.64E-04 |
| *O. sinensis* | 30 | 126079 | F | 30 | 156391 | 6.64E-04 |
| *O. sinensis* | 30 | 109764 | P | 30 | 157203 | 6.64E-04 |
| *O. sinensis* | 30 | 55806 | P | 30 | 157176 | 2.37E-05 |
| *O. sinensis* | 30 | 151462 | P | 30 | 157110 | 6.64E-04 |
| *O. sinensis* | 30 | 128850 | P | 30 | 157099 | 6.05E-09 |
| *O. sinensis* | 30 | 16467 | P | 30 | 157099 | 2.37E-05 |
| *O. sinensis* | 30 | 23737 | P | 30 | 157100 | 6.64E-04 |
| *O. sinensis* | 30 | 157082 | P | 30 | 157087 | 6.64E-04 |
| *O. sinensis* | 30 | 109837 | P | 30 | 157087 | 6.64E-04 |
| *O. sinensis* | 30 | 113404 | P | 30 | 157069 | 5.45E-07 |
| *O. sinensis* | 30 | 120911 | P | 30 | 156168 | 6.64E-04 |
| *O. sinensis* | 30 | 55718 | P | 30 | 151473 | 2.37E-05 |
| *O. sinensis* | 30 | 120941 | P | 30 | 151473 | 2.37E-05 |
| *O. sinensis* | 30 | 55509 | P | 30 | 151473 | 6.64E-04 |
| *O. sinensis* | 30 | 150079 | P | 30 | 151473 | 6.64E-04 |
| *O. sinensis* | 30 | 86133 | P | 30 | 150188 | 6.64E-04 |
| *O. sinensis* | 30 | 128882 | P | 30 | 150159 | 6.64E-04 |
| *O. sinensis* | 30 | 86178 | P | 30 | 150131 | 6.64E-04 |
| *O. sinensis* | 30 | 57005 | P | 30 | 150129 | 6.64E-04 |
| *O. sinensis* | 30 | 113386 | P | 30 | 150066 | 6.64E-04 |
| *O. sinensis* | 30 | 109859 | P | 30 | 150044 | 6.64E-04 |
| *O. sinensis* | 30 | 13668 | P | 30 | 150007 | 6.64E-04 |
| *O. sinensis* | 30 | 149141 | P | 30 | 149143 | 6.64E-04 |
| *O. sinensis* | 30 | 28735 | P | 30 | 146502 | 2.37E-05 |
| *O. sinensis* | 30 | 26984 | P | 30 | 141213 | 2.37E-05 |
| *O. sinensis* | 30 | 17417 | P | 30 | 141213 | 6.64E-04 |
| *O. sinensis* | 30 | 56926 | P | 30 | 141213 | 6.64E-04 |
| *O. sinensis* | 30 | 109741 | P | 30 | 141159 | 5.45E-07 |
| *O. sinensis* | 30 | 56926 | P | 30 | 140362 | 6.64E-04 |
| *O. sinensis* | 30 | 109741 | P | 30 | 140308 | 5.45E-07 |
| *O. sinensis* | 30 | 121495 | P | 30 | 139991 | 6.64E-04 |
| *O. sinensis* | 30 | 120911 | P | 30 | 139993 | 6.64E-04 |
| *O. sinensis* | 30 | 86133 | P | 30 | 135336 | 6.64E-04 |
| *O. sinensis* | 30 | 29456 | P | 30 | 135153 | 2.37E-05 |
| *O. sinensis* | 30 | 17483 | P | 30 | 135128 | 2.37E-05 |
| *O. sinensis* | 30 | 27050 | P | 30 | 135128 | 2.37E-05 |
| *O. sinensis* | 30 | 120815 | P | 30 | 135128 | 6.64E-04 |
| *O. sinensis* | 30 | 109022 | P | 30 | 135100 | 6.05E-09 |
| *O. sinensis* | 30 | 109722 | P | 30 | 128875 | 6.64E-04 |
| *O. sinensis* | 30 | 109853 | P | 30 | 128851 | 2.37E-05 |
| *O. sinensis* | 30 | 87590 | P | 30 | 128848 | 6.64E-04 |
| *O. sinensis* | 30 | 109824 | P | 30 | 126624 | 2.37E-05 |
| *O. sinensis* | 30 | 26984 | P | 30 | 126134 | 6.64E-04 |
| *O. sinensis* | 30 | 16532 | P | 30 | 126080 | 6.64E-04 |
| *O. sinensis* | 30 | 110136 | P | 30 | 123582 | 6.64E-04 |
| *O. sinensis* | 30 | 108089 | P | 30 | 122788 | 5.45E-07 |
| *O. sinensis* | 30 | 23041 | P | 30 | 122788 | 5.45E-07 |
| *O. sinensis* | 30 | 56394 | P | 30 | 122788 | 2.37E-05 |
| *O. sinensis* | 30 | 28735 | P | 30 | 121891 | 6.64E-04 |
| *O. sinensis* | 30 | 109824 | P | 30 | 121587 | 6.64E-04 |
| *O. sinensis* | 30 | 87594 | P | 30 | 121487 | 6.64E-04 |
| *O. sinensis* | 30 | 92758 | P | 30 | 121492 | 6.64E-04 |
| *O. sinensis* | 30 | 17523 | P | 30 | 120941 | 6.64E-04 |
| *O. sinensis* | 30 | 113386 | P | 30 | 120928 | 2.37E-05 |
| *O. sinensis* | 30 | 120923 | P | 30 | 120928 | 6.64E-04 |
| *O. sinensis* | 30 | 55700 | P | 30 | 120928 | 6.64E-04 |
| *O. sinensis* | 30 | 55891 | P | 30 | 120908 | 2.37E-05 |
| *O. sinensis* | 30 | 92758 | P | 30 | 120910 | 2.37E-05 |
| *O. sinensis* | 30 | 113534 | P | 30 | 120906 | 6.64E-04 |
| *O. sinensis* | 30 | 29418 | P | 30 | 120822 | 6.64E-04 |
| *O. sinensis* | 30 | 113386 | P | 30 | 113391 | 6.64E-04 |
| *O. sinensis* | 30 | 55491 | P | 30 | 113391 | 6.64E-04 |
| *O. sinensis* | 30 | 55700 | P | 30 | 113391 | 2.37E-05 |
| *O. sinensis* | 30 | 94231 | P | 30 | 113373 | 6.64E-04 |
| *O. sinensis* | 30 | 92758 | P | 30 | 113373 | 6.64E-04 |
| *O. sinensis* | 30 | 109722 | P | 30 | 111784 | 6.64E-04 |
| *O. sinensis* | 30 | 92756 | P | 30 | 111760 | 6.64E-04 |
| *O. sinensis* | 30 | 55636 | P | 30 | 109915 | 2.37E-05 |
| *O. sinensis* | 30 | 23737 | P | 30 | 109855 | 6.64E-04 |
| *O. sinensis* | 30 | 109837 | P | 30 | 109842 | 6.64E-04 |
| *O. sinensis* | 30 | 84240 | P | 30 | 109824 | 6.64E-04 |
| *O. sinensis* | 30 | 28148 | P | 30 | 109765 | 6.64E-04 |
| *O. sinensis* | 30 | 86078 | P | 30 | 109736 | 6.64E-04 |
| *O. sinensis* | 30 | 14322 | P | 30 | 109022 | 2.37E-05 |
| *O. sinensis* | 30 | 28741 | P | 30 | 108948 | 6.64E-04 |
| *O. sinensis* | 30 | 86178 | P | 30 | 108937 | 5.45E-07 |
| *O. sinensis* | 30 | 27060 | P | 30 | 108937 | 6.64E-04 |
| *O. sinensis* | 30 | 57003 | P | 30 | 108937 | 2.37E-05 |
| *O. sinensis* | 30 | 94182 | P | 30 | 108666 | 6.64E-04 |
| *O. sinensis* | 30 | 29456 | P | 30 | 108666 | 2.37E-05 |
| *O. sinensis* | 30 | 17257 | P | 30 | 108045 | 6.64E-04 |
| *O. sinensis* | 30 | 55769 | P | 30 | 98157 | 5.45E-07 |
| *O. sinensis* | 30 | 55762 | P | 30 | 98108 | 5.45E-07 |
| *O. sinensis* | 30 | 30833 | P | 30 | 98108 | 5.45E-07 |
| *O. sinensis* | 30 | 17398 | P | 30 | 94254 | 2.37E-05 |
| *O. sinensis* | 30 | 23737 | P | 30 | 94231 | 6.64E-04 |
| *O. sinensis* | 30 | 86161 | P | 30 | 94166 | 6.64E-04 |
| *O. sinensis* | 30 | 14346 | P | 30 | 92749 | 6.64E-04 |
| *O. sinensis* | 30 | 23747 | P | 30 | 92748 | 2.37E-05 |
| *O. sinensis* | 30 | 28716 | P | 30 | 87562 | 6.64E-04 |
| *O. sinensis* | 30 | 56975 | P | 30 | 87556 | 5.45E-07 |
| *O. sinensis* | 30 | 17520 | P | 30 | 84243 | 6.64E-04 |
| *O. sinensis* | 30 | 29451 | P | 30 | 56975 | 6.64E-04 |
| *O. sinensis* | 30 | 55804 | P | 30 | 55804 | 2.37E-05 |
| *O. sinensis* | 30 | 23496 | P | 30 | 55771 | 6.64E-04 |
| *O. sinensis* | 30 | 23490 | P | 30 | 55763 | 6.64E-04 |
| *O. sinensis* | 30 | 17523 | P | 30 | 55718 | 6.64E-04 |
| *O. sinensis* | 30 | 55700 | P | 30 | 55705 | 6.64E-04 |
| *O. sinensis* | 30 | 27645 | P | 30 | 55637 | 2.37E-05 |
| *O. sinensis* | 30 | 23490 | P | 30 | 30834 | 6.64E-04 |
| *O. sinensis* | 30 | 8640 | P | 30 | 8644 | 6.64E-04 |
| *O. sinensis* | 30 | 126021 | R | 30 | 126023 | 2.37E-05 |
| *O. sinensis* | 30 | 55807 | R | 30 | 55810 | 6.64E-04 |
| *O. sinensis* | 30 | 98107 | R | 30 | 23491 | 6.64E-04 |
| *O. sinensis* | 30 | 55762 | R | 30 | 55763 | 5.45E-07 |
| *O. sinensis* | 30 | 30833 | R | 30 | 55763 | 5.45E-07 |
| *O. sinensis* | 30 | 55762 | R | 30 | 30834 | 5.45E-07 |
| *O. sinensis* | 30 | 30833 | R | 30 | 30834 | 5.45E-07 |
| *O. sinensis* | 30 | 98107 | R | 30 | 98108 | 5.45E-07 |
| *O. sinensis* | 30 | 23490 | R | 30 | 98108 | 6.64E-04 |
| *O. sinensis* | 30 | 1602 | R | 30 | 1602 | 2.37E-05 |
| *O. sinensis* | 30 | 121535 | R | 30 | 121536 | 5.45E-07 |
| *O. sinensis* | 30 | 146547 | R | 30 | 121536 | 5.45E-07 |
| *O. sinensis* | 30 | 121535 | R | 30 | 146548 | 5.45E-07 |
| *O. sinensis* | 30 | 146547 | R | 30 | 146548 | 5.45E-07 |
| *O. sinensis* | 30 | 121536 | R | 30 | 121940 | 2.37E-05 |
| *O. sinensis* | 30 | 146548 | R | 30 | 121940 | 2.37E-05 |
| *O. sinensis* | 30 | 121942 | R | 30 | 121544 | 2.37E-05 |
| *O. sinensis* | 30 | 121942 | R | 30 | 146556 | 2.37E-05 |
| *O. sinensis* | 30 | 121941 | R | 30 | 121945 | 6.64E-04 |
| *O. sinensis* | 30 | 121542 | R | 30 | 121544 | 2.37E-05 |
| *O. sinensis* | 30 | 146554 | R | 30 | 121544 | 2.37E-05 |
| *O. sinensis* | 30 | 121542 | R | 30 | 146556 | 2.37E-05 |
| *O. sinensis* | 30 | 146554 | R | 30 | 146556 | 2.37E-05 |
| *O. sinensis* | 30 | 55763 | C | 30 | 98157 | 5.45E-07 |
| *O. sinensis* | 30 | 30834 | C | 30 | 98157 | 5.45E-07 |
| *O. sinensis* | 30 | 55763 | C | 30 | 23498 | 6.64E-04 |
| *O. sinensis* | 30 | 30834 | C | 30 | 23498 | 6.64E-04 |
| *O._xuefengensis* | 138 | 35328 | F | 138 | 37568 | 1.44E-74 |
| *O._xuefengensis* | 124 | 447 | F | 124 | 17782 | 3.23E-59 |
| *O._xuefengensis* | 66 | 40084 | F | 66 | 41773 | 6.18E-27 |
| *O._xuefengensis* | 57 | 49342 | P | 57 | 52466 | 1.44E-23 |
| *O._xuefengensis* | 53 | 40097 | F | 53 | 41786 | 3.42E-21 |
| *O._xuefengensis* | 51 | 53515 | F | 51 | 55523 | 3.44E-22 |
| *O._xuefengensis* | 50 | 70870 | F | 50 | 70933 | 1.52E-17 |
| *O._xuefengensis* | 50 | 52003 | P | 50 | 55524 | 2.06E-19 |
| *O._xuefengensis* | 50 | 52003 | P | 50 | 53516 | 2.06E-19 |
| *O._xuefengensis* | 50 | 17625 | P | 50 | 28056 | 7.28E-16 |
| *O._xuefengensis* | 49 | 49350 | F | 49 | 51916 | 2.74E-15 |
| *O._xuefengensis* | 49 | 49349 | F | 49 | 76445 | 5.82E-17 |
| *O._xuefengensis* | 49 | 52467 | P | 49 | 76445 | 2.74E-15 |
| *O._xuefengensis* | 48 | 10510 | F | 48 | 51915 | 1.03E-14 |
| *O._xuefengensis* | 48 | 51916 | F | 48 | 76446 | 1.03E-14 |
| *O._xuefengensis* | 48 | 24935 | F | 48 | 61333 | 1.03E-14 |
| *O._xuefengensis* | 48 | 17629 | F | 48 | 76441 | 2.23E-16 |
| *O._xuefengensis* | 47 | 43305 | F | 47 | 43389 | 3.85E-14 |
| *O._xuefengensis* | 47 | 10511 | F | 47 | 76446 | 3.85E-14 |
| *O._xuefengensis* | 47 | 10511 | F | 47 | 49350 | 3.85E-14 |
| *O._xuefengensis* | 46 | 28056 | P | 46 | 76441 | 1.44E-13 |
| *O._xuefengensis* | 46 | 28056 | P | 46 | 49345 | 1.44E-13 |
| *O._xuefengensis* | 44 | 17633 | F | 44 | 49349 | 4.80E-14 |
| *O._xuefengensis* | 44 | 17633 | P | 44 | 52472 | 2.01E-12 |
| *O._xuefengensis* | 43 | 10511 | F | 43 | 17634 | 7.51E-12 |
| *O._xuefengensis* | 42 | 61374 | F | 42 | 67361 | 6.99E-13 |
| *O._xuefengensis* | 42 | 28056 | F | 42 | 52474 | 2.79E-11 |
| *O._xuefengensis* | 42 | 27125 | P | 42 | 34330 | 2.79E-11 |
| *O._xuefengensis* | 41 | 43320 | F | 41 | 43404 | 1.04E-10 |
| *O._xuefengensis* | 41 | 17634 | F | 41 | 51916 | 2.66E-12 |
| *O._xuefengensis* | 41 | 51916 | P | 41 | 52474 | 1.04E-10 |
| *O._xuefengensis* | 41 | 28056 | P | 41 | 51916 | 2.66E-12 |
| *O._xuefengensis* | 41 | 10511 | P | 41 | 28056 | 1.04E-10 |
| *O._xuefengensis* | 40 | 531 | F | 40 | 17866 | 1.01E-11 |
| *O._xuefengensis* | 39 | 61384 | F | 39 | 67371 | 1.42E-09 |
| *O._xuefengensis* | 39 | 50901 | F | 39 | 56470 | 1.42E-09 |
| *O._xuefengensis* | 39 | 653 | F | 39 | 1731 | 3.85E-11 |
| *O._xuefengensis* | 38 | 9557 | P | 38 | 53784 | 5.26E-09 |
| *O._xuefengensis* | 37 | 51309 | F | 37 | 52251 | 1.94E-08 |
| *O._xuefengensis* | 37 | 43280 | F | 37 | 43411 | 1.94E-08 |
| *O._xuefengensis* | 37 | 50891 | F | 37 | 56460 | 1.94E-08 |
| *O._xuefengensis* | 37 | 39933 | F | 37 | 41629 | 1.94E-08 |
| *O._xuefengensis* | 37 | 49371 | P | 37 | 52457 | 1.94E-08 |
| *O._xuefengensis* | 36 | 14314 | F | 36 | 41716 | 2.09E-09 |
| *O._xuefengensis* | 36 | 52486 | P | 36 | 76439 | 7.12E-08 |
| *O._xuefengensis* | 36 | 55052 | P | 36 | 57541 | 7.12E-08 |
| *O._xuefengensis* | 36 | 53787 | P | 36 | 57028 | 2.09E-09 |
| *O._xuefengensis* | 35 | 24937 | F | 35 | 67322 | 7.91E-09 |
| *O._xuefengensis* | 35 | 40018 | F | 35 | 41714 | 2.61E-07 |
| *O._xuefengensis* | 35 | 24948 | F | 35 | 61346 | 7.91E-09 |
| *O._xuefengensis* | 35 | 17340 | P | 35 | 27159 | 7.91E-09 |
| *O._xuefengensis* | 34 | 543 | F | 34 | 17878 | 9.55E-07 |
| *O._xuefengensis* | 34 | 10504 | P | 34 | 52488 | 9.55E-07 |
| *O._xuefengensis* | 34 | 27148 | P | 34 | 34315 | 9.55E-07 |
| *O._xuefengensis* | 33 | 30012 | F | 33 | 44017 | 3.48E-06 |
| *O._xuefengensis* | 33 | 27572 | F | 33 | 53786 | 1.12E-07 |
| *O._xuefengensis* | 33 | 17375 | F | 33 | 34334 | 1.12E-07 |
| *O._xuefengensis* | 33 | 29343 | F | 33 | 35273 | 2.34E-09 |
| *O._xuefengensis* | 33 | 37678 | F | 33 | 38224 | 3.48E-06 |
| *O._xuefengensis* | 33 | 9560 | P | 33 | 27572 | 3.48E-06 |
| *O._xuefengensis* | 32 | 14315 | F | 32 | 40021 | 1.27E-05 |
| *O._xuefengensis* | 32 | 70668 | F | 32 | 70704 | 1.27E-05 |
| *O._xuefengensis* | 32 | 50912 | F | 32 | 56481 | 1.27E-05 |
| *O._xuefengensis* | 32 | 20506 | F | 32 | 34457 | 1.27E-05 |
| *O._xuefengensis* | 32 | 25108 | F | 32 | 67478 | 9.08E-09 |
| *O._xuefengensis* | 32 | 26927 | P | 32 | 26927 | 4.22E-07 |
| *O._xuefengensis* | 31 | 61335 | F | 31 | 67322 | 4.59E-05 |
| *O._xuefengensis* | 31 | 40180 | F | 31 | 41844 | 4.59E-05 |
| *O._xuefengensis* | 31 | 31984 | F | 31 | 61850 | 4.59E-05 |
| *O._xuefengensis* | 31 | 25060 | F | 31 | 61440 | 1.58E-06 |
| *O._xuefengensis* | 31 | 40190 | F | 31 | 41854 | 4.59E-05 |
| *O._xuefengensis* | 31 | 25116 | F | 31 | 61502 | 4.59E-05 |
| *O._xuefengensis* | 31 | 43280 | F | 31 | 43386 | 4.59E-05 |
| *O._xuefengensis* | 31 | 10501 | F | 31 | 76436 | 4.59E-05 |
| *O._xuefengensis* | 31 | 51434 | P | 31 | 53833 | 4.59E-05 |
| *O._xuefengensis* | 31 | 51938 | P | 31 | 52462 | 4.59E-05 |
| *O._xuefengensis* | 31 | 20504 | P | 31 | 29346 | 4.59E-05 |
| *O._xuefengensis* | 30 | 49372 | F | 30 | 51938 | 1.66E-04 |
| *O._xuefengensis* | 30 | 21032 | F | 30 | 40430 | 5.92E-06 |
| *O._xuefengensis* | 30 | 14434 | F | 30 | 56474 | 1.66E-04 |
| *O._xuefengensis* | 30 | 30136 | F | 30 | 44135 | 5.92E-06 |
| *O._xuefengensis* | 30 | 8139 | F | 30 | 51841 | 1.66E-04 |
| *O._xuefengensis* | 30 | 43292 | F | 30 | 43423 | 1.66E-04 |
| *O._xuefengensis* | 30 | 14473 | F | 30 | 40190 | 1.66E-04 |
| *O._xuefengensis* | 30 | 20805 | F | 30 | 41930 | 1.66E-04 |
| *O._xuefengensis* | 30 | 43284 | F | 30 | 43331 | 1.66E-04 |
| *O._xuefengensis* | 30 | 15595 | F | 30 | 16109 | 5.92E-06 |
| *O._xuefengensis* | 30 | 14397 | F | 30 | 40103 | 1.66E-04 |
| *O._xuefengensis* | 30 | 14397 | F | 30 | 41792 | 1.66E-04 |
| *O._xuefengensis* | 30 | 36804 | F | 30 | 53782 | 1.66E-04 |
| *O._xuefengensis* | 30 | 44178 | F | 30 | 67417 | 1.66E-04 |
| *O._xuefengensis* | 30 | 25061 | F | 30 | 67422 | 1.66E-04 |
| *O._xuefengensis* | 30 | 43384 | F | 30 | 43409 | 1.66E-04 |
| *O._xuefengensis* | 30 | 39941 | F | 30 | 41637 | 1.66E-04 |
| *O._xuefengensis* | 30 | 75448 | P | 30 | 75448 | 5.92E-06 |
| *O._xuefengensis* | 30 | 27568 | P | 30 | 57039 | 1.66E-04 |
| *O._xuefengensis* | 30 | 42044 | P | 30 | 56427 | 1.66E-04 |
| O._xuefengensis | 30 | 14432 | P | 30 | 55777 | 1.66E-04 |
| O._xuefengensis | 30 | 20510 | P | 30 | 53782 | 1.36E-07 |
| O._xuefengensis | 30 | 15712 | P | 30 | 53625 | 5.92E-06 |
| O._xuefengensis | 30 | 20509 | P | 30 | 36805 | 1.66E-04 |
| O._xuefengensis | 30 | 17380 | P | 30 | 34336 | 5.92E-06 |
| O._xuefengensis | 30 | 17652 | P | 30 | 28049 | 1.66E-04 |
